# DeepPROTACs is a deep learning-based targeted degradation predictor for PROTACs

**DOI:** 10.1038/s41467-022-34807-3

**Published:** 2022-11-21

**Authors:** Fenglei Li, Qiaoyu Hu, Xianglei Zhang, Renhong Sun, Zhuanghua Liu, Sanan Wu, Siyuan Tian, Xinyue Ma, Zhizhuo Dai, Xiaobao Yang, Shenghua Gao, Fang Bai

**Affiliations:** 1 Shanghai Institute for Advanced Immunochemical Studies, ShanghaiTech University, 393 Middle Huaxia Road, Shanghai, 201210 China; 2 School of Information Science and Technology, ShanghaiTech University, 393 Middle Huaxia Road, Shanghai, 201210 China; 3 Gluetacs Therapeutics (Shanghai) Co., Ltd., 99 Haike Road, Zhangjiang Hi-Tech Park, Shanghai, 201210 China; 4 School of Life Science and Technology, ShanghaiTech University, 393 Middle Huaxia Road, Shanghai, 201210 China; 5 Shanghai Clinical Research and Trial Center, Shanghai, 201210 China

**Keywords:** Machine learning, Computational models, Drug discovery and development, Computational chemistry

## Abstract

The rational design of PROTACs is difficult due to their obscure structure-activity relationship. This study introduces a deep neural network model - DeepPROTACs to help design potent PROTACs molecules. It can predict the degradation capacity of a proposed PROTAC molecule based on structures of given target protein and E3 ligase. The experimental dataset is mainly collected from PROTAC-DB and appropriately labeled according to the *DC*_50_ and *Dmax* values. In the model of DeepPROTACs, the ligands as well as the ligand binding pockets are generated and represented with graphs and fed into Graph Convolutional Networks for feature extraction. While SMILES representations of linkers are fed into a Bidirectional Long Short-Term Memory layer to generate the features. Experiments show that DeepPROTACs model achieves 77.95% average prediction accuracy and 0.8470 area under receiver operating characteristic curve on the test set. DeepPROTACs is available online at a web server (https://bailab.siais.shanghaitech.edu.cn/services/deepprotacs/) and at github (https://github.com/fenglei104/DeepPROTACs).

## Abstract (web summary)

The rational design of PROTACs is difficult due to their obscure structure-activity relationship. Here the authors present a deep neural network model - DeepPROTACs - for predicting the degradation capacity of a proposed PROTAC molecule.

## Introduction

Traditional therapeutics rely on small-molecule inhibitors to implement occupancy-driven pharmacology as the mode of action (MOA). Despite the great success, this MOA suffers from several limitations such as inability to deal with undruggable targets^1–3^, off-target toxicity^4^, undesired side effects^5^, drug resistance^6,7^, and so on. As a result, monoclonal antibodies and RNA interference (RNAi) approaches start to complement small-molecule inhibitor paradigm^8,9^. Although antibodies possess high binding affinities to extracellular protein targets and RNAi abolish protein levels at low doses, their weaknesses limit their therapeutic applications. Antibodies are difficult to cross cell membranes and RNAi molecules own poor oral bioavailability and tissue distribution. Therefore, new modalities should combine the advantages of small-molecule, antibody, and RNAi methods and overcome their disadvantages.

**Fig. 1:**
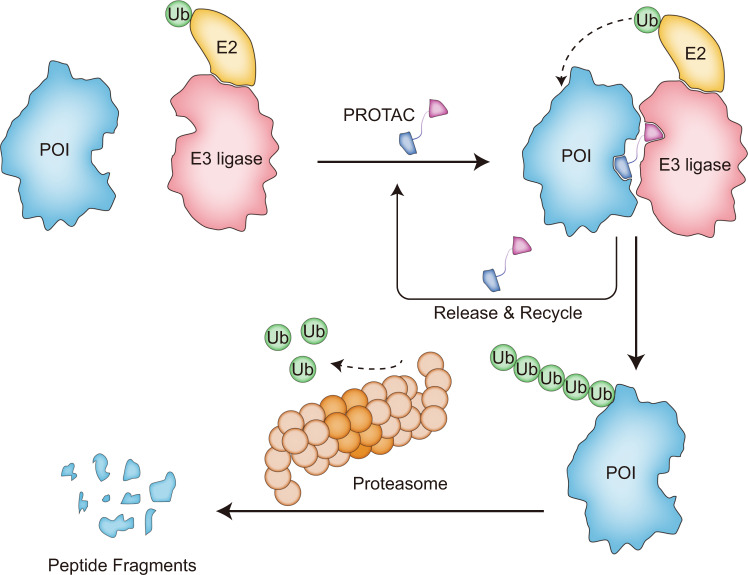
The degradation mechanism of POI (Protein of Interest) by PROTACs. The POI and E3 ligase are brought together by PROTACs, which facilitates the transfer of ubquitin. The POI labeled with ubquitin is recognized by proteasome and degraded into peptides.

PROteolysis TArgeting Chimeras (PROTACs) has become an appealing technology to utilize the event-driven MOA since its birth at 2001^10^. A PROTAC is a heterobifunctional molecule composed of a protein of interest (POI) ligand, a linker and an E3 ubiquitin ligase recruiting ligand. It promotes the formation of a ternary complex (POI-PROTAC-E3) by bringing the ubiquitination machinery to the proximity of POI, driving the transfer of ubiquitin from E2 enzyme to the exposed lysine on target protein. Subsequently, the polyubiquitination occurs and the ubiquitinated POI is recognized by 26 S proteasome and degraded into small peptide fragments or even amino acids (Fig. 1)^11–14^. Since 26 S proteasome belongs to the ubiquitin-proteasome system (UPS) in eukaryotic cells, PROTACs actually represents a chemical knockdown approach that hijacks the UPS to regulate the intracellular protein levels^15–17^. The concept of PROTAC was first proposed and proved by Crews and coworkers. They successfully induced the degradation of methionine aminopeptidase-2 (MetAp-2) by recruiting a peptidic SCF-βTRCP E3 moiety^10^. The advancement of all small-molecule-based PROTACs was achieved in 2008. A well-known MDM2-p53 PPI inhibitor - Nutlin had been employed to couple with an androgen receptor (AR) ligand to build a PROTAC, which resulted in the degradation of AR in prostate tumor cells^18^. In 2012, a series of peptidomimetic von Hippel-Lindau (VHL) ligands that reserve the vital hydroxyproline residue were reported^19–21^. Comparing with previous peptidic counterparts, these ligands possessed similar binding affinities to VHL but more preferable physicochemical properties^22^. Concurrently, the immunomodulatory drugs (IMiDs), including thalidomide, pomalidomide, and lenalidomide, were indicated to bind the cereblon (CRBN) E3 ligase, laying the foundation for constructing new PROTACs that employ CRBN^23–25^.

As a novel and promising technique, PROTACs displays a variety of superior properties in comparison to current treatment methods. First, PROTACs is capable to modulate the undruggable targets that lack of a classical hydrophobic drug binding pocket or strongly bind with endogenous molecules^26–28^. Further, it can also tackle the proteins that function through protein-protein interactions^29^. Escaping the demand for blocking the catalytic activity or protein-protein interface, PROTACs can recruit a ligand that binds anywhere on the target protein with relatively low affinity. Second, PROTACs act catalytically because they are released from the ternary complex once the ubiquitination process is completed (Fig. 1). Due to this catalytical nature, PROTACs can play a role at low exposures, reducing the potential for off-target and other undesirable effects. Third, the accumulation of target proteins is frequently observed in inhibitor-based methods on account of the protein stabilization by drug binding and transcriptional upregulation of proteins^30,31^. This exerts adverse effects to the efficacy of inhibitor. However, the target accumulation can be avoided by employing PROTACs because they eliminate the whole proteins through proteasome. Additionally, this also indicates that PROTACs can modulate nonenzymatic/scaffolding functions and address the problem of drug resistance arose from the mutations surrounding the binding pocket^32–36^. Finally, improved selectivity among closely related proteins can be provided by applying PROTACs^17,37,38^. The active sites of homologous proteins are highly conserved, while the sequence and conformation outside the catalytic core maybe of great change. PROTACs can exploit this difference to degrade the specific targets as the ubiquitin transfer step depends on the relative location of exposed lysine and ubiquitin. It means that the conformation of ternary complex is of great significance for the development of potent PROTACs. The conformation is largely dependent on the PROTACs linker, which becomes one of the central tasks for PROTACs design. It is difficult to design a universal linker that is suitable for all cases due to the different structures of target proteins and E3 ligases.

Over the past few years, the production of enormous, high-quality data in biological systems has accelerated the applications of artificial intelligence (AI) approaches within the field of drug discovery and development^39–44^. Meanwhile, the wide availability of huge storage systems and graphical processing units (GPUs) also facilitates large-scale parallel computing, which can speed up the numerically intensive computations. In contrast to explicitly constructing physical models, AI, especially machine learning (ML) techniques, use mathematical algorithms to discover complex relationships between existing datasets and make accurate predictions on new samples^45,46^. Artificial neural networks (ANNs) are believed to be one of the most powerful ML frameworks. They imitate the neurons in brain structure. Owing to the nonlinear activation functions applied to each neuron unit and multi-layer structures, ANNs are capable of learning complicated relationships between inputs and outputs within the datasets^47–50^. As a modern reincarnation of ANNs, deep learning (DL) make use of deep and sophisticated structures to extract valuable features from massive amounts of training data^51^. On the basis of diverse network architectures, DL can be classified into several subclasses: multilayer perceptron (MLP), convolutional neural networks (CNNs), recurrent neural networks (RNNs), graph convolutional neural networks (GCNs), and so on. Among these models, RNNs have been widely utilized in sequential data analysis, for example, natural language processing, while CNNs have attained huge successes for regular Euclidean data, for example, images in computer vision field^52,53^. GCNs attempt to leverage the key ingredients of CNNs and learn features of a target node by taking the information of itself as well as its locally connected neighborhoods on the graph into consideration. GCNs have shown great success for feature extraction on non-Euclidean geometric data such as graphs and manifolds^54,55^.

Before the uptake of DL by the pharmaceutical industry, the success rate of drug development is very low^56^. It has to go through target validation, high-throughput screening, lead optimization, pre-clinical trials, and clinical trials, all of which are tremendous costly and time-consuming. For PROTACs, efficient molecules with appropriate linkers can only be obtained through laborious trial-and-error processes. Hence, it is reasonable to introduce DL technologies into pharmaceutical field for the purpose of lowering the overall costs and shortening the development cycle. ML or DL can be applied in nearly all stages of drug development. For small-molecule design and optimization, many computational models have been constructed to perform virtual screening (VS) using ML-based approaches^57,58^. Nonetheless, DL have been demonstrated to be much more effective than the other competing methods^59,60^. Increasing quantitative structure-activity relationship (QSAR) models have been built by DL techniques to correlate chemical structures of molecules with their physiochemical properties, biological activities, and ADMET properties^61–64^. Converting molecular structure into proper descriptor or feature vector is the top priority of almost all DL tasks. After that, an optimal mapping between input features and network responses can be achieved by an iterative training process.

As far as we know, there were no reports concerning the application of DL in the field of PROTACs due to the deficiency of experimental data. However, this situation has been changed recently. Continuous efforts of more than 20 years have accumulated plenty of high-quality data for PROTACs. On the other hand, the rapid development of DL also makes it more powerful in revealing the potential mappings between inputs and outputs. These provides a fantastic opportunity to integrate DL with PROTACs. Recently, Hou et al. published an online database of PROTACs (PROTAC-DB)^65^ that includes 2258 PROTACs, 275 warheads (small molecules that bind POI), 68 E3 ligands (small molecules that recruit E3 ligases) and 1099 linkers. In addition, we have also collected more data (375 PROTACs targeting 30 POIs) from other public sources. PROTAC-DB offers the binding affinities, degradation efficiencies and cellular activities for various PROTACs. Therefore, it is convenient for us to acquire labeled data from this database. In this study, we introduce DeepPROTACs, a deep neural network model that can efficiently predict the degradation efficacy of given PROTACs based on the structures of POI and E3 ligase. Our framework embeds different parts of a given POI-PROTAC-E3 ligase complex with separate neural network modules. The component embeddings are concatenated together before feeding into an MLP with two fully connected layers to get the final output. The average accuracy rate and AUROC (area under ROC) of this model on test set can be up to 77.95% and 0.8470, respectively. We further validated this model by using a batch of PROTACs that recruited VHL to destroy estrogen receptor (ER). Among these total 16 PROTACs, this model can successfully predict the degradation capacities of 11, thus attaining 68.75% prediction accuracy. For other recently reported PROTAC targets (EZH2, STAT3, eIF4E, and FLT-3), the accuracy rates change in the scope of 65% to 80%. All these results have demonstrated the capability of our DeepPROTACs model.

## Results and discussion

### The architecture of deepPROTACs

**Fig. 2:**
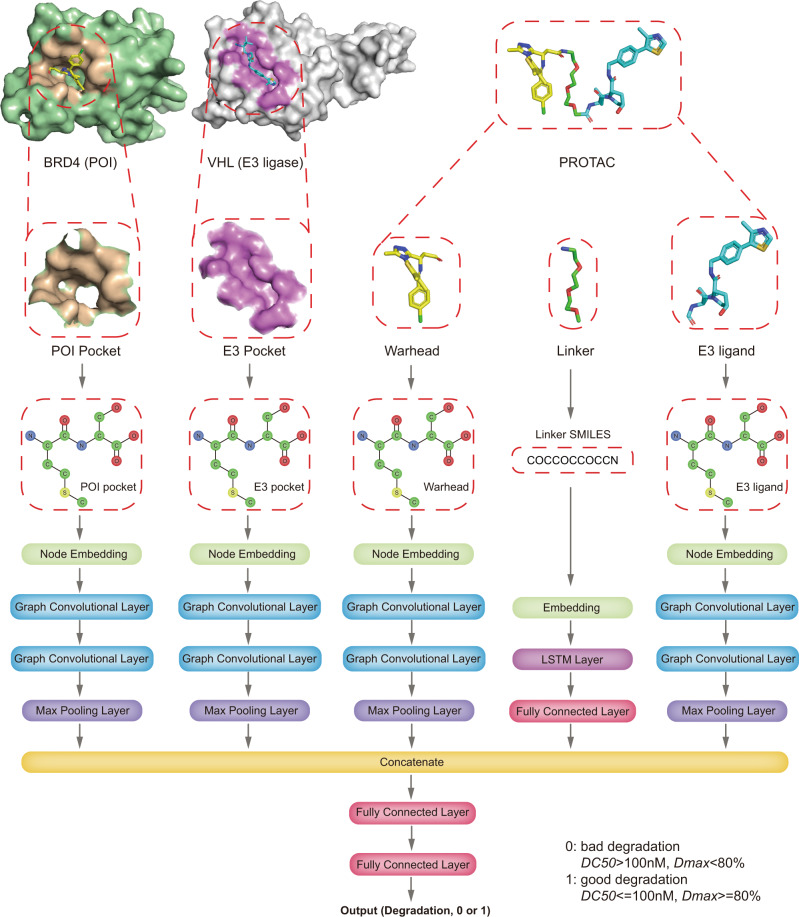
The network architecture of DeepPROTACs. The preprocess of POI, E3 ligase and PROTAC molecule: extraction of binding pocket from POI (BRD4) and E3 ligase (VHL) and conversion to graph representations, conversion of PROTAC molecule to graph representations and SMILES.

The network architecture of DeepPROTACs is illustrated in Fig. 2. We implemented the whole network with the PyTorch^66^ and PyTorch Geometric^67^ frameworks. In our experiments, all GCNs consist of two graph convolutional layers followed by a max pooling layer. The output pocket/ligand representation of each max pooling has a dimension of 64. Besides, the encoding of linker SMILES is fed into an embedding layer to obtain the distributed representation. This operation is much better than one-hot encoding because the latter treats all characters as independent entities without relationship to each other. Subsequently, the output of this embedding layer is fed in a Bidirectional LSTM layer with 64 nodes and a fully connected layer successively. The output representation of linker SMILES network module also has a dimension of 64. The result of this linker network is concatenated with the results of pocket/ligand networks before being fed into an MLP with two fully connected layers to obtain the final output. Leaky rectified linear unit (Leaky ReLU) is used as the activation function in this network. It is worth noting that the weights of GCNs for POI pockets and E3 pockets are shared, and the weights of GCNs for warheads and E3 ligands are also shared. Detailed output dimensions of each layer and hyperparameters utilized in the DeepPROTACs model are listed in Table 1. In order to compare our model with baseline methods, we have further trained several traditional ML models, including Support Vector Machine (SVM) and Random Forest (RF) by employing the auto cross-covariance (ACC) as the feature of protein. The ligand is represented by the molecular access system (MACCS) keys or Morgan fingerprints. ACC transforms a protein sequence into an 18 bits vector, while MACCS keys and Morgan fingerprints represent a small molecule with a 166 bits vector and a 1024 bits vector, respectively. The representation of a ternary complex is constructed by concatenating the features of the target protein, the E3 ligase, and the PROTAC molecule. SVM models are built in Scikit-learn package and set kernel as linear, regularization parameter C as 1; RF models are also built in Scikit-learn package and set number of estimators as 100, max depth as 5.

**Table 1:** The dimensions and parameters of DeepPROTACs model

DeepPROTACs model	Layer	Output dimension
Protein pockets/ligands	Node embedding	64
	Graph convolution layer 1	128
	Graph convolution layer 2	64
SMILES	Embedding	64
	Bidirectional LSTM	64
	Fully connected layer	64
MLP	Fully connected layer 1	64
	Fully connected layer 2	2
Parameters	Epoch	30
	Batch size	1
	Number of GCN layers	2
	Pocket size	5 Å
	Inclusion of bond type encoding	Yes
	Learning rate	0.0001
	Pooling layer	Max pooling
	Loss	Cross-entropy
	Optimizer	Adam

### The optimization of model parameters

Different model parameters have been tested to optimize the DeepPROTACs model (Table 2). In each experiment, the whole dataset was randomly split into the training set, validation set, and test set at a ratio of 8:1:1. Under the circumstance of batch size 1, the training for the model was finished at epoch 30. Eventually, this model achieves an average accuracy of 77.15% on the validation set. The other values of batch size (the corresponding epoch number is determined according to the loss of validation set) have also been examined and it turns out that these values result in performance reduction in varying degrees (Table 2). Two consecutive GCN layers are applied in the network architecture because the same setting was used in the original paper^68^. Furthermore, we have investigated the effect of GCN layer numbers and observed the best performance at layer number of two (Table 2). The effects of pocket size on the model are also investigated by gradually enlarging the pocket size. As listed in Tabel 2, the model using 5 Å pocket size performs the best. The other pocket sizes reduce the predicting power of the model at different levels. Although larger pocket size is more likely to cover the residues on contacting surface of POI and E3 ligase, it may also include many irrelevant residues inside the protein, leading to the addition of noise signal. Further, it is difficult to determine a uniform pocket size to cover the contacting surface because the structures of different POI/E3 ligase are distinct and the conformations of generated ternary complexes are also dependent on the structures of PROTACs. Instead, 5 Å size pocket only contains the first and second coordination shell residues around ligand, which are known in POI/warhead and E3/ligand structures. It should be preferable to determine the degradation result by utilizing the confirmed information and circumventing the uncertain factors. In addition, the inclusion of max pooling layer and bond type encoding improves the model’s performance (Table 2). Consequently, the final model is trained by the optimized parameters listed in Table 1 and by the Adam optimizer^69^ with the learning rate of 0.0001, β1 of 0.9, and β2 of 0.999. The objective is cross entropy for binary classification.

**Table 2:** The optimization of DeepPROTACs model hyperparameters on validation set

Hyperparameters	Values	Average accuracy	AUROC
Batch size	**1**	**77.15%**^a^	**0.8246**^a^
	8	75.85%^a^	0.8009^a^
	16	75.85%^a^	0.8183^a^
	32	76.32%^a^	0.8118^a^
	64	76.21%^a^	0.8212^a^
	128	76.09%^a^	0.8249^a^
	256	75.62%^a^	0.8246^a^
Number of GCN layers	one	75.38%^a^	0.7850^a^
	**two**	**77.15%**^a^	**0.8246**^a^
	three	76.68%^a^	0.8201^a^
Pocket size	**5 Å res around ligand**	**77.15%**^a^	**0.8246**^a^
	10 Å res around ligand	76.33%^a^	0.8114^a^
	15 Å res around ligand	73.73%^a^	0.8010^a^
	All protein	73.97%^a^	0.7780^a^
Pooling layer	**Max pooling**	**77.15%**^a^	**0.8246**^a^
	Mean pooling	72.44%^a^	0.7692^a^
	Sum pooling	72.20%^a^	0.7733^a^
Inclusion of bond type encoding	No	77.15%^a^	0.8246^a^
	**Yes**	**77.97%**^a^	**0.8278**^a^
Optimized hyperparameters	use	77.46%^b^	0.8531^b^

Note: ^a^ metrics value on validation set, ^b^ metrics value on test set; bold means optimal parameters.

**Fig. 3:**
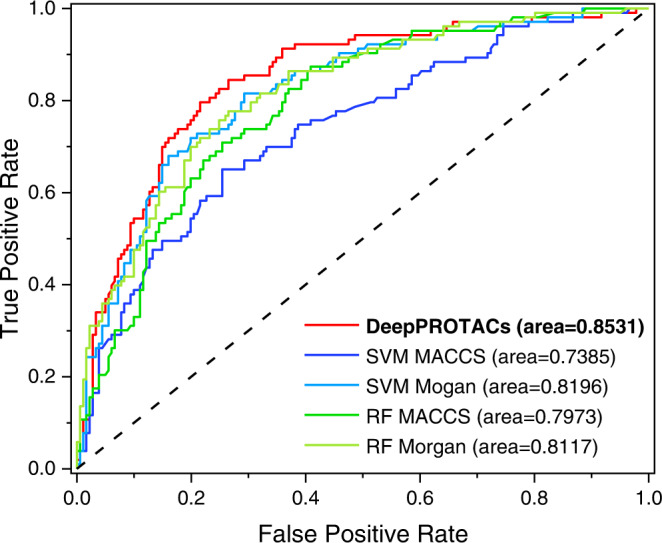
ROC curves of DeepPROTACs, SVM (Support Vector Machine), and RF (Random Forest) models. Source Data are provided as a Source Data file.

After three times of repeated training, the DeepPROTACs model achieves an average accuracy rate of 77.46% and 0.8531 AUROC on test set that is 10% of whole data (Table 3). In contrast to the metrics of SVM and RF models using different fingerprints (Table 3 and Fig. 3), this is an impressive achievement. The performance of SVM model with Morgan fingerprints has improved a lot when comparing with MACCS keys. However, it is still inferior to GCN model, especially, in terms of AUROC. With respect to RF model, the average accuracy approaches 70% and the AUROC is around 0.80, both of which are not better than those of SVM and GCN models. For the following experiments, the division of 8:2 (training:test) is adopted to sufficiently inspect the predicting capability of DeepPROTACs model on a larger test set. The results suggest that DeepPROTACs maintains high performance (77.95% accuracy and 0.8470 AUROC) on the test set, that is 20% of whole data. The true positive rate and precision rate are calculated to be 85.37% and 80.98% (Supplementary Table 1), respectively, elucidating the good sensitivity and precision of our model. For the purpose of comparison, we constructed two alternative models that treat the whole PROTAC molecule as graph and SMILES, respectively (Supplementary Figs. 1 and 2). Consequently, there are only three network modules in these two models before concatenation: POI pocket module, E3 pocket module, and PROTAC graph or SMILES module. The network module for graph or SMILES is the same as DeepPROTACs model. However, the predicting accuracies of these two models on the test set are computed to be 68.08% and 76.25%, respectively, which are lower than that of the DeepPROTACs model. Separate representations of PROTAC molecules in DeepPROTACs can not only decrease the sparseness of the adjacency matrix in graph representation but also reveal the hidden mappings between the linker and degradation efficacy. Thus, these two alternative models are discarded and the following experiments focus on the DeepPROTACs model.

**Table 3:** The evaluation results of DeepPROTACs, SVM, and RF models on test set

Model	Fingerprints	Average accuracy	AUROC
DeepPROTACs	-	77.46%	0.8531
SVM	MACCS	71.48%	0.7385
	Morgan	76.76%	0.8196
RF	MACCS	68.31%	0.7973
	Morgan	69.37%	0.8117

### Architecture validation and data balance

**Fig. 4:**
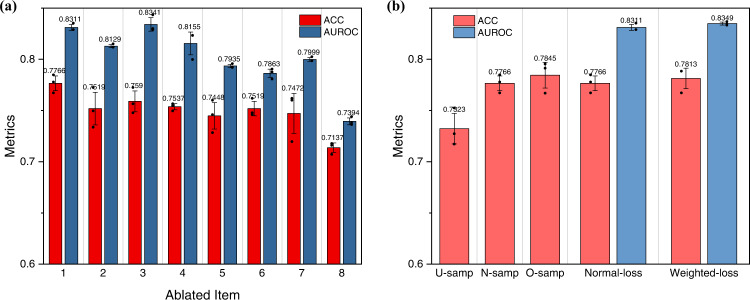
Validation of DeepPROTACs model and data balance inspection on N = 2832 biologically independent samples over 3 independent experiments. Data are presented as mean values ± SD. The statistical test used for data analysis is paired *t* test with two-sided and no adjustments were made for multiple comparisons. Source data are provided as a Source Data file. **a** Ablation experiments on DeepPROTACs model. Ablated item: 1 - none, 2 - ligase pocket, 3 - E3 ligand, 4 - POI pocket, 5 - warhead, 6 - linker, 7 - ligase pocket and E3 ligand, 8 - POI pocket and warhead. **b** Data balance experiments on DeepPROTACs model. Under-sampling (U-samp): deleting redundant inactive samples (the number of active and inactive samples is 988:988). Normal-sampling (N-samp): adopting original samples (the number of active and inactive samples is 988:1844). Over-sampling (O-samp): repeated sampling part of active samples (the number of active and inactive samples is 1844:1844). Weighted-loss: the weight of loss corresponding to the active samples was multiplied by 2, while the weight of loss corresponding to the inactive samples was unchanged.

Ablation experiments on DeepPROTACs model were carried out in order to validate the current network architecture. As illustrated in Fig. 4a, the elimination of ligase pocket, E3 ligand, POI pocket or warhead (ablated item: 2, 3, 4, 5) from current architecture indeed attenuates the performance of the GCN model. Deleting the linker input (ablated item: 6) also leads to inferior performance. Further, in contrast to the ablation of single item, the removal of double items such as ligase pocket/E3 ligand or POI pocket/warhead (ablated item: 7, 8) further reduces the predicting accuracy and AUROC. In brief, these experiments have demonstrated the indispensability of every part in current DeepPROTACs model. Moreover, the training process of DeepPROTACs model was repeated by using three different training/test splits. For each split, the whole dataset was randomly divided into a training set and a test set at a ratio of 8:2. And in each case of split, the model was trained for three times. The evaluation metrics of each split are quite similar, illustrating the robustness and reproducibility of our GCN model.

According to Supplementary Table 2, the activity distribution of PROTAC molecules varies substantially among different pairs of target proteins and E3 ligases. Consequently, it is almost impossible to achieve data balance within a single specific pair of target protein and E3 ligase. Instead, we attempt to investigate the influence of data balance on the whole dataset. The ratio of active and inactive samples in original dataset is 988:1844. Except for normal-sampling, we conduct under-sampling by deleting some redundant inactive samples and over-sampling by repeatedly sampling part of active samples. As a result, the ratio of active and inactive samples in under-sampling and over-sampling become 988:988 and 1844:1844, respectively. In each sampling method, we trained the model for three times and obtained an average predicting accuracy and AUROC. Our experimental results show that the over-sampling method performs the best, followed by the normal-sampling and the under-sampling methods (Fig. 4b). It seems to be reasonable because over-sampling takes full advantage of limited data, while under-sampling wastes some data resources. In addition, the weighted loss is also utilized as another method to ameliorate the impact of data imbalance. Specifically, the weight of loss corresponding to the active samples is multiplied by a factor of 2, while the weight of loss corresponding to the inactive samples is kept unaltered. Comparing with the case of the normal loss, both accuracy and AUROC in the weighted-loss case increase slightly (Fig. 4b). These results elucidate that data imbalance problem indeed exists in this study, while the efforts that attempt to solve this problem only improve the model’s performance marginally. Hence, considering the balance between performance and computational cost, we adopted the default settings (normal-sampling and normal-loss) for the training of the final DeepPROTACs model.

Except for the default active/inactive cutoff (100 nM in *DC*_*50*_ and 80% in *Dmax*), we have considered another one (1000 nM in *DC*_*50*_ and 70% in *Dmax*) to reduce the influence of artificial factors. The PROTAC molecule is only considered as active if both *DC*_*50*_ and *Dmax* satisfy the cutoff condition. We have retrained the DeepPROTACs model according to the new label. The average accuracy and AUROC are computed to be 76.42% and 0.8283, respectively, both of which are quite similar to the values obtained by using the original label. This illuminates the stability of our model regardless of the subjective selection of cutoff criterion. For the sake of simplicity, only the first label is utilized for the training of the final DeepPROTACs model.

### Evaluation of deepPROTACs

**Fig. 5:**
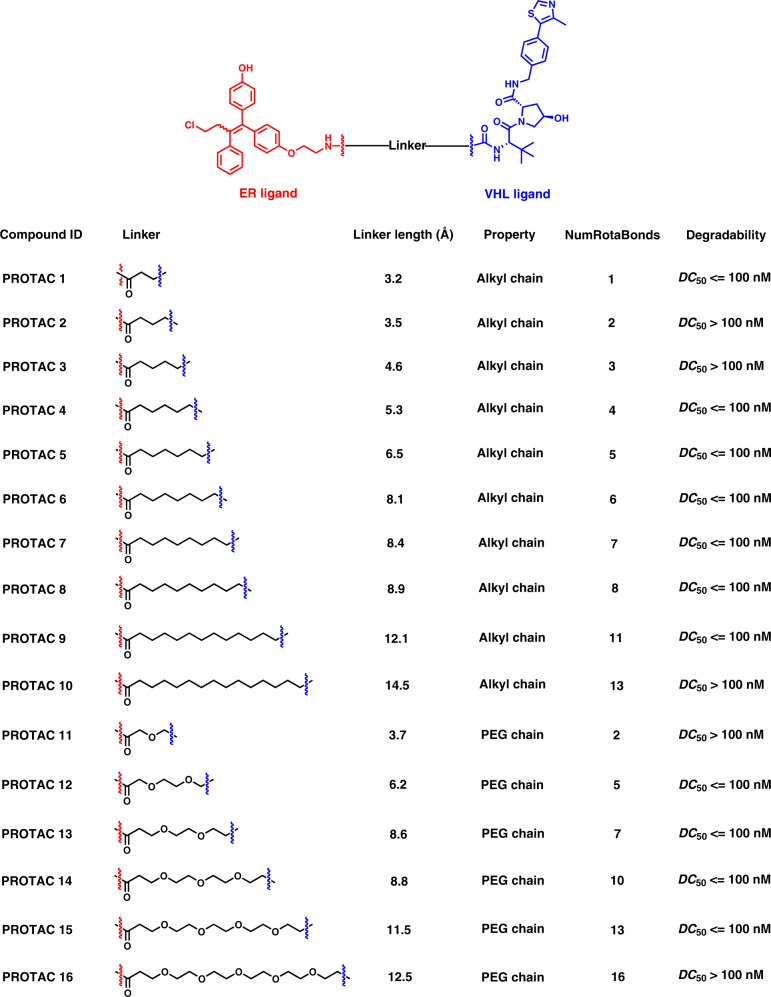
Chemical structures and properties of 16 PROTACs in our experimental dataset. PROTAC 1–10 are built with linkers of alkyl chain, while PROTAC 11–16 are built with linkers of PEG chain.

**Fig. 6:**
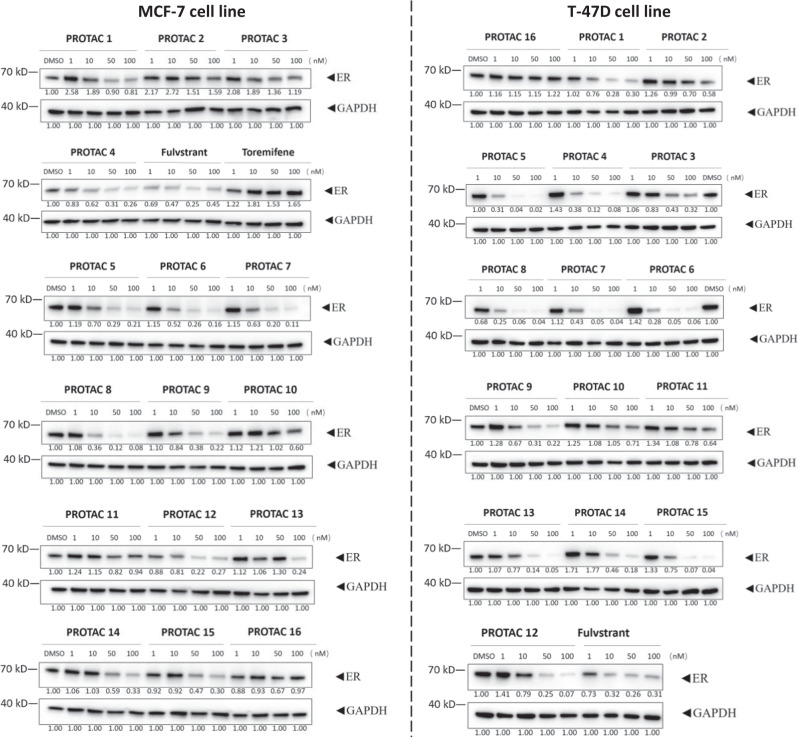
Western blotting analysis and densitometry quantifications of ER protein. The MCF-7 and T-47D breast cancer cell lines are treated with indicated compounds at 1, 10, 50 and 100 nM for 16 h. The densitometry quantifications are normalized by GAPDH using the Image J software. All western blot data are representative of at least two independent replicates.

To verify the predictive capability of DeepPROTACs, we constructed an experimental dataset containing 16 PROTACs (Fig. 5) that recruit VHL E3 ligase to degrade ER. As members of nuclear receptor family, ERs are transcription factors mediating gene expression and influencing the biological effects of estrogen. Degradation of the ER protein is beneficial to patients with ER positive (ER + ) breast cancer that occurs in around 80% of newly diagnosed breast cancer cases^70^. A selective ER modulator - toremifene is employed as the ligand of target protein and a peptidomimetic compound is applied as VHL ligand. Various linear alkyl chains and polyethylene glycol (PEG) chains with different lengths are selected as linkers. These PROTACs are evaluated for their ability to induce ER degradation in MCF-7 and T-47D breast cancer cell lines, with fulvstrant and toremifene used as the control. Western blotting data (Fig. 6 and Supplementary Table 3) show that 11 compounds (PROTAC 1, PROTAC 4 to 9, PROTAC 12 to 15) are very potential in inducing ER degradation at concentrations lower than 100 nM within 16 h treatment. Therefore, they are considered as good degraders. The other 5 PROTACs (PROTAC 2 to 3, PROTAC 10 to 11, PROTAC 16) are less effective or ineffective in degrading ER at indicated concentrations, meaning that they belong to bad degraders according to our standard of classification. The VHL/ER binding assay and Western blotting analysis of PROTAC 8 and its negative analogue PROTAC 8N (contains inactive isomer of VHL ligand) were also conducted in T-47D cell line. Calculated from thermodynamic data in Supplementary Fig. 3a, the *K*_d_ value of PROTAC 8 to VHL is determined to be 4.37 μM, while there is almost no binding between PROTAC 8N and VHL. The *K*_d_ values of PROTAC 8 and 8N to ER are measured to be 2.14 and 2.82 μM, respectively. In addition, ER is almost completely degraded by PROTAC 8 at indicated concentrations (100/1000 nM), while PROTAC 8N is not able to degrade ER (Supplementary Fig. 3b). These results confirm the binding of ER PROTACs to VHL ligase and ER protein, suggesting that the degradation of ER is indeed implemented through ubiquitin-proteasome pathway. The DeepPROTACs model successfully predicts the degradation labels of 11 compounds among 16 PROTACs (Supplementary Table 4), reaching 68.75% prediction accuracy rate.

The targets (EZH2, STAT3, eIF4E, and FLT-3) in Supplementary Table 5 were treated as novel targets to further inspect the generalization ability of our model. Specifically, for each target, the model was trained in the absence of this target and then utilized to predict the degradation of the test set and corresponding target. All predicting accuracies on test set are quite similar, fluctuating around the value of 77%. And the accuracies on specific targets vary within the range from 65% to 80%, illustrating that our model possesses good generalization capability. The selection of 5 Å pocket size maybe beneficial for this because it only provides the protein environments around ligand instead of the whole protein as input, alleviating the impact of different protein structures. The relative instability of model’s generalization performance on various targets may associate with the limited PROTACs data. In future, the access of larger amounts of data and the retraining of our model with these data may solve this problem.

### Computational Modeling: ER PROTACs

**Fig. 7:**
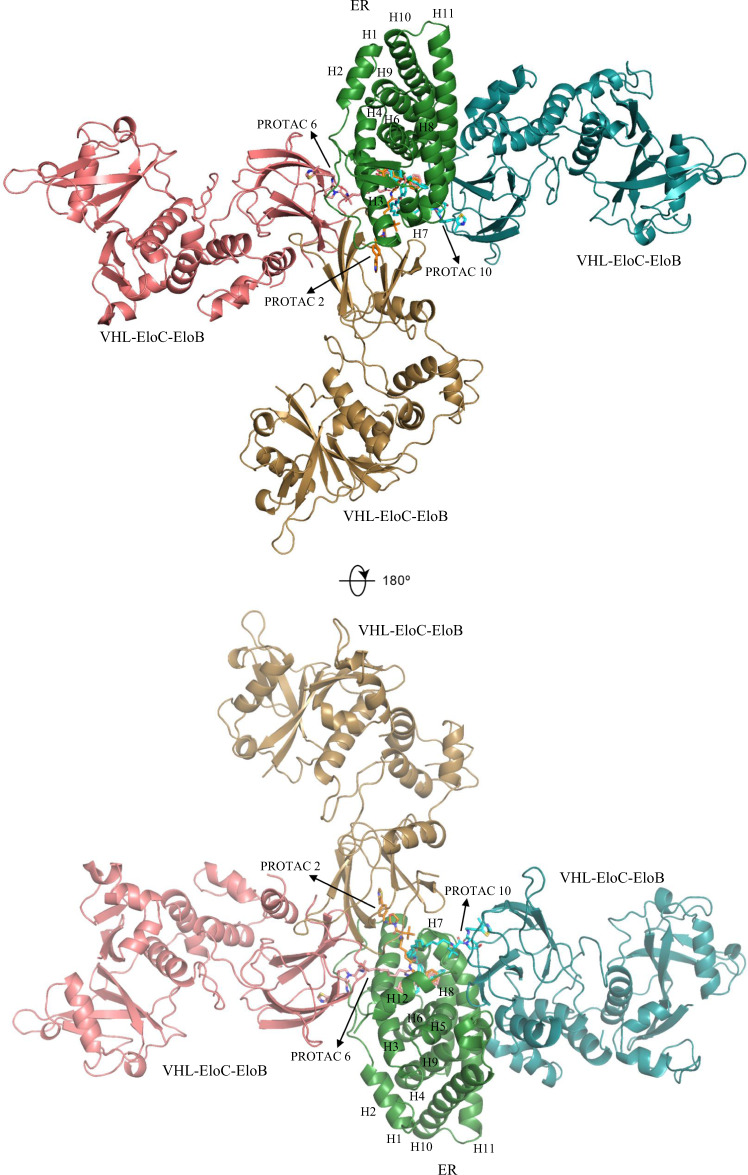
Computational models of ternary complexes constituted by ER, VHL-EloC-EloB and PROTACs (PROTAC 2, 6, and 10) from two views. All models are aligned based on the position of ER protein (green). The VHL-EloC-EloB and PROTAC molecule are marked with wheat, pink, and cyan color in complex induced by PROTAC 2, 6, and 10, respectively.

Compounds PROTAC 2, PROTAC 6, and PROTAC 10 have been confirmed to be bad, good, and bad degraders, respectively (Supplementary Table 4). Their linker lengths were calculated to be 3.5, 8.1, and 14.5 Å^71^, representing short, medium, and long alkyl linkers, respectively (Fig. 5). Hence, they were selected to build ternary complexes through PRosettaC and Molecular Dynamics (MD) simulations to correlate linker with degradation capability. The crystal structures of ER (PDB ID: 1ERR^72^ [https://www.rcsb.org/structure/1ERR]) and VHL-Eloc-ELoB (PDB ID: 5T35^73^ [https://www.rcsb.org/structure/5T35]) were obtained from Protein Data Bank (PDB)^74^. As displayed in Fig. 7, the most representative structures of these complexes were aligned based on the position of ER protein. ER or actually ER ligand-binding domain (LBD) is folded into a three-layered antiparallel α-helical sandwich, which contains a central core layer of three helices (H5/6, H9, and H10) that is clamped by two other layers of helices (H1-4; H7, H8, and H11)^72^. A sizable ligand-binding cavity is observed at the narrow end of the ‘wedge-shaped’ molecular scaffold created by this helical arrangement. The short linker in PROTAC 2 has only two rotatable bonds, leading to its poor flexibility. As a result, ER and VHL come together to sandwich PROTAC 2 between their respective binding pockets, burying otherwise solvent-exposed portions of two heads. In addition, the two heads recapitulate the binding modes of respective ligands individually in each binding site. Several favorable intermolecular interactions (ER-VHL: D332-R108, N348-Y112, D351-R69) are observed between ER and VHL to stabilize the ternary complex. As the chain grows, flexibility is added into the system to change the overall structural conformation. In the PROTAC 6 - induced complex, VHL is shifted to the left direction in comparison with previous complex, forming electrostatic contacts (ER-VHL: R352-Y98, H356-H110, E542-R69) mainly with H3/H12 helices of ER. The long linker in PROTAC 10 results in a parallel conformation of ternary complex, in which the interactions (ER-VHL: E380-Q96, R515-Q195, R434-E173) occur mostly between VHL and H8/H11 helices of ER. In this complex, the two heads are completely exposed to solvent, while the *tert*-butyl group of VHL ligand is trapped by a hydrophobic region comprised by V534, P535, Y537 of ER, and W88, F91 of VHL. Similar results are also observed for complexes built by PROTACs with PEG linkers. To assess the role of surface lysine residues in degradability, we constructed the whole CRLs structure by superimposing the ternary complexes with crystal structures of Cul2-Rbx1-EloBC-VHL (PDB ID: 5N4W^75^ [https://www.rcsb.org/structure/5N4W]) and NEDD8-CUL1-RBX1-UB~UBE2D2 (PDB ID: 6TTU^76^ [https://www.rcsb.org/structure/6TTU]). As shown in Supplementary Figs. 4–6, six surface-exposed lysine residues (K362, K467, K472, K481, K492, and K520) are accessible to E2-ubiquitin (E2-Ub) in PROTAC 6 - induced model at distances between 40–60 Å, while only 3 lysine residues are approachable in both PROTAC 2 - and PROTAC 10 - induced models at relatively longer distances of 45–55 and 50–80 Å, respectively. This indicates that different linkers contribute to distinct conformations of ternary complexes and thus the whole CRLs structures, altering the accessibility of surface lysine residues to Ub. Although the ubiquitination zone of CRLs is large and flexible in some degree, this may still exert a profound effect on degradation ability. Medium-length linkers are correlated with good degradability because they provide both flexibility and stability for the ternary complex. Even though ternary complex stability and protein cooperativity will inevitably influence the depletion efficiency of target protein, yet it is beyond our research scope to consider all these factors here because the main purpose of this study is to construct a high-throughput virtual screening tool for PROTACs. In future, detailed researches concerning PROTACs QSAR should consider both factors by utilizing advanced computational techniques such as umbrella sampling simulation and binding affinity calculation.

Overall, combining the 77.95% average accuracy rate and 0.8470 AUROC on test set with the 65% to 80% accuracy rate on ER experimental dataset and other novel targets, it is convincing to state that the DeepPROTACs model is able to predict the degradation ability of novel PROTACs providing the structures of POI, E3 ligase, and PROTACs. Furthermore, it’s also possible to improve the performance of our model if PROTACs data experience rapid growth in the coming years. And there is another possibility to increase the precision of targeted design by training a model towards a particular target protein if there is sufficient data on this target. Anyway, before immersing into the hard work of wet lab, a round of virtual screening by our model seems to be very essential. It can help reduce the efforts to design potent PROTACs for specific target proteins and may be beneficial for developing small molecules that can exploit novel E3 ligases. In order to enable worldwide access to this protocol, we have made it available through a web server (https://bailab.siais.shanghaitech.edu.cn/services/deepprotacs/) and github (https://github.com/fenglei104/DeepPROTACs).

## Discussion

In this study, based upon the data from PROTAC-DB and other public sources, we have trained a DL model - DeepPROTACs to address the difficulties in designing potent PROTAC molecules especially their linkers. The model can predict the ability of given PROTACs to induce the degradation of a specific POI by recruiting a specific E3 ligase. The structure-activity relationship of PROTACs remain elusive because the number of crystal structure of POI-PROTAC-E3 ternary complex is limited. Furthermore, it is very trivial and time-consuming to model all these complexes by current computational methods. Therefore, we circumvented the building of ternary complex and imported the structures of POI pocket, E3 pocket, and PROTACs into the network model separately. And we concatenated all output vectors before exporting the final results. In test set, the DeepPROTACs model is around 78% accurate in predicting the degradation label of PROTACs, while in our experimental dataset and novel targets the accuracy rate is fluctuating in the range from 65% to 80%. Given the warhead and E3 ligand, the linker between them can either be manually designed or generated through the linker generation method^77^. The generated PROTAC molecules can be virtually screened by our model before committing to synthesis and biological experiments. This can reduce both the cost and time of drug discovery. In addition, computational modeling of ternary complexes has been conducted on several selected PROTACs in experimental dataset to investigate the relationship between linker and degradation capability. The results illuminate that the linkers with appropriate medium lengths lead to better protein depletion probably owing to the accessibility of surface lysine residues to ubiquitin and the stability of ternary complex.

Although the model is thought to be successful, there is still room for improvement. Graph representation of ligands does not take the chirality of small molecules into consideration. Whereas in some cases, the chirality plays an important role in determining the degradation effect of PROTAC molecules^21^. Thus, atom coordinates of ligands and pockets can be included in the graph to investigate the effects of chirality. Further, although we have collected more data outside the PROTAC-DB to expand our dataset, 2832 labeled dataset is still believed to be a small dataset by AI specialists. It is rewarding to utilize a special semi-supervised learning approach that combines a small quantity of labeled data with large amount of unlabeled data during the training process to compensate the deficiency of annotated samples and improve the predicting accuracy for small datasets^78^. The unlabeled PROTACs can be created using the linker generation method mentioned above. By enlarging our dataset, the binary classification model can also be transformed to multi-class classification model or even regression model. This may further predict the range or even exact values of *DC*_50_ and *Dmax* of PROTACs. Anyway, our DeepPROTACs model is a successful attempt to integrate AI into the field of PROTACs. It will not only serve as important guidelines for the design of potent PROTACs, but also provide research paradigm for the combination of AI with drug discovery.

## Methods

### Data curation and labeling

Our criterion for labeling the data is *DC*_50_ (half maximal degradation concentration) and *Dmax* (maximal degradation): only compounds with *DC*_50_ lower than 100 nM and *Dmax* higher than 80% are labeled as good degraders, otherwise, they are tagged with bad degraders. After this operation, the total 2832 data were separated into 988 ‘good’ data and 1844 ‘bad’ data. For turning the hyperparameters, the entire dataset was randomly split into training, validation, and test set at a ratio of 8:1:1. After optimizing the hyperparameters, the whole dataset was randomly divided into a training set and test set at a ratio of 8:2 for the following experiments because a larger test set would decrease the standard deviation between different training trials. The total number of POI-E3 pairs reaches 218. The structures of POI and E3 ligases in complex with their corresponding inhibitors were obtained from Protein Data Bank (PDB)^74^. For ligands that were not available in the crystal structure of the protein, the binding poses were computationally predicted or aligned to the ligand with similar scaffolds. Then, the steepest descent minimization was performed using Yasara software^79^ to remove remaining clashes, followed by a simulated annealing minimization with atomic velocities scaled down by 0.9 every ten steps to reach a stable local minimum. The structures of PROTACs, as well as their physicochemical properties, including molecular weight, partition coefficient (logP), aqueous solubility (logS), heavy atom count, ring count, hydrogen bond acceptor/donor count, and topological polar surface area were computed by RDKit toolkit (https://www.rdkit.org) or provided by PROTAC-DB.

### Implementation details of network modules

In this section, we present each network module that embeds different components of the complex and discuss about the implementation details. In most existing literatures^80–82^, proteins were represented by sequence (FASTA) and fed into sequential-based models to learn distributed characteristics. However, in this work, instead of learning features from the string representation of the whole protein, we propose to learn features from the graph representation of extracted protein pocket using GCNs. There are several considerations for this switch: First, proteins are composed of atoms that are connected to each other through covalent bonds, thus belonging to graph structures naturally. Using GCNs to characterize the topological structure of proteins has biological significance. Second, residues that are distant from ligand binding pocket exhibit little effect on the construction of ternary complex. Focusing on the pocket can reduce the size of neural network input substantially. Third, graph representation could preserve the structural information of extracted pocket to some extent. Atoms are connected to multiple neighbors and this information is stored in an adjacency matrix. As displayed in Fig. 2, the pockets of both POI and E3 ligase (taking BRD4 and VHL as examples^73^) were extracted by selecting residues within 5.0 Å around the binding ligand using PyMol software (the PyMOL Molecular Graphics System, Version 2.0 Schrӧdinger, LLC). The extracted structures were then converted to Mol2 files^83,84^, which contain all the information necessary to reconstruct molecular topologies. Graph representations were built based on this information by utilizing the adjacency matrix. In these graphs, atoms were represented by nodes, with 0, 1, 2, 3, 4 signifying C, N, O, S, and other atom types, while covalent bonds were represented by edges. If there was a bond between two atoms, then the corresponding position on the adjacency matrix was labeled as 1. Otherwise, the corresponding position was labeled as 0. The POI ligands (warheads) as well as E3 ligands, were also transferred to graph representations. However, every node here had 10 possible atom types: C, N, O, S, F, Cl, Br, I, P, and other atoms, each of which was denoted by 0, 1, 2, 3, 4, 5, 6, 7, 8, 9, respectively. In addition, the edge types in both protein and ligand graphs included single bond, double bond, triple bond, aromatic bond, and amide bond, each of which was encoded by 1, 2, 3, 4, 5, respectively. The SMILES of PROTACs linkers were encoded according to an encoding table (Supplementary Table 6) derived from ZINC database^85^. We collected the SMILES of total 2,076,017 lead-like molecules that have standard reactivity and are in stock. The number of occurrences of each character in these SMILES was counted and summarized in Supplementary Table 6 as frequency. The 39 characters with the highest frequencies were encoded from 1 to 39, while the [PAD] token and the rest of characters were encoded as 0 and 40, respectively.

### Computational modeling methods

The computational modeling of ternary complexes that are composed of POI, PROTAC, and E3 ligase has been performed through PRosettaC^86^ and Molecular Dynamics (MD) simulations in a sequential manner. PRosettaC is a combined protocol for modeling a ternary complex induced by a given PROTAC. It alternates between sampling of the protein-protein interaction space and the PROTAC molecule conformational space to produce near-native predictions of ternary complexes. The clustered poses provided by this protocol were utilized as the starting points for MD simulations, which were performed using the GROMACS 2019 program^87^ and the AMBER14^88^ force field. The restrained electrostatic potential charges (RESP) of PROTAC molecules were calculated using Gaussian 09 program^89^ and then used to generate topology files by antechamber^90^, an in-built tool in AMBER^91^. Generalized AMBER force field (GAFF)^92^ was applied to parameterize the PROTACs. For each MD simulation, the starting structure of POI-PROTAC-E3 ternary complex was placed in a cubic box with dimensions that set the nearest distance of complex to each boundary surface to 20 Å. This box was saturated by TIP3P^93^ water molecules, some of which were substituted by sodium and chloride ions to neutralize the system and to simulate a physiological ion concentration of 154 mM. The system was then subjected to energy minimization with a steepest descent method until the maximum force was smaller than a tolerant value of 100 [kJ mol^−1^ nm^−1^]. *NVT* and *NPT* equilibrations were subsequently performed to equilibrate the system to a predefined temperature of 310 K and pressure of 1 bar. The result of these equilibrations produced the initial structure for MD simulation, which was run for 100 ns with *NPT* ensemble. The LINCS algorithm^94^ was utilized to constrain the bond lengths of peptide, and the SETTLE algorithm^95^ was employed to constrain the bond lengths and angles of water molecules. The long-range electrostatic interactions were computed by the particle mesh Ewald (PME) method^96^ with a cutoff value of 1.2 nm. The amino acid residues were set to their normal ionization states at pH 7.0 and the MD trajectory was generated with a time step of 2 fs. The most representative structure of ternary complex was derived from the cluster analysis of MD trajectory, in which the frame with smallest average RMSD from all other structures of the cluster was selected as the central structure to represent the cluster that has been constructed by grouping together the structurally similar frames (root-mean-square deviations - RMSD cutoff is set to 0.3 nm).

### General experimental methods

#### Chemical materials

All chemicals were obtained from commercial suppliers (Adamas and Alfa), and used without further purification, unless otherwise indicated. VHL-1 based acid and cis VHL-1 based acid were prepared according to the ref. 97. HPLC preparation was performed on SHIMADZU LC-20AP instrument with original column. All new compounds were characterized by 1H NMR and HRMS. 1H NMR spectra were recorded on Bruker AVANCE III 500 MHZ (operating at 500 MHz for 1H NMR), chemical shifts were reported in ppm relative to the residual CDCl3 (7.26 ppm 1H), DMSO-d6 (2.50 ppm 1H) or Methanol-d4 (3.31 ppm, 1H), and coupling constants (J) are given in Hz. Multiplicities of signals are described as follows: s --- singlet, d --- doublet, t --- triplet, m --- multiple. High Resolution Mass Spectra were recorded on AB Triple 4600 spectrometer with acetonitrile and water as solvent.

#### Cell lines and cell culture

The human breast cancer cell line MCF-7 was purchased from American Type Culture Collection (ATCC Number: HTB-22). T-47D cell line was purchased from Shanghai Cell Bank of the National Science Academy of China (Shanghai) (Catalog number: TChu 87). All these cells were cultured according to the provider’s instructions and maintained at 37 °C in a humidified atmosphere containing 5% CO2 in air. Cell lines were examined as mycoplasma free.

#### Western blotting

1.5 × 105 cells/ml were plated in 24-well plates and treated with DMSO or compounds at the indicated concentrations for 16 h. Cells were collected, washed with cold 1 x PBS, and lysed in 1x SDS buffer containing protease inhibitor cocktails (#539134, Merck). Protein in cell lysate was quantified by detergent-compatible Bradford assay kit (#23246, Thermo). Primary antibodies used in this study include ER antibody (#8644 S, Cell Signaling Technology, 1:1000 dilution in blocking buffer), Anti-Rabbit IgG, HRP-linked (#7074P2, Cell Signaling Technology, 1:5000 dilution in blocking buffer) and GAPDH antibody (#8884 S, Cell Signaling Technology, 1:5000 dilution in blocking buffer). The Millipose Immobilon Western Chemiluminescence Substrate was used for signal development. Blots were imaged in an Amersham Imager 600 (GE Healthcare).

#### Protein expression and purification

The cDNA fragment encoding ERα-LBD (aa 298–554) was subcloned into pET15b vector and transfect into E.coli BL21 (DE3) cells for protein expression. Cells were crushed and the supernatant was incubated with nickel affinity chromatography, further eluted with buffer of 20 mM Tris pH 8.0, 50 mM NaCl, 1 mM TCEP, 5% glycerol and 300 mM imidazole. Protein was concentrated and purified with gel filtration chromatography (Superdex200 HiLoad) in 20 mM Tris pH 8.0, 100 mM NaCl, 1 mM TCEP, 5% glycerol. For the expression of His-tagged VCB complex, the cDNA fragment encoding VHL (aa 54-213) was co-transformed with a pCDF Duet plasmid containing EloB (aa 1-104) and EloC (aa 1-112) into E.coli BL21 (DE3) cells for protein expression. After expression by IPTG induction, cells were collected and resuspended in 50 mM Tris pH 7.5, 500 mM NaCl, 5% glycerol, 10 mM imidazole and 1 mM TCEP. The lysate was incubated with Ni-NTA beads and eluted with lysis buffer containing 300 mM imidazole. The protein was further purified by Superdex200 with buffer of 25 mM Tris pH 7.6, 150 mM NaCl, 1 mM TCEP.

#### Isothermal titration calorimetry (ITC) measurements

All measurements were performed by PEAQ-ITC (Malvern) in buffer of 20 mM Tris pH 8.0, 100 mM NaCl, 1 mM TCEP, 100 mM β-cyclodextrin, 5% glycerol while stirring at 750 rpm. The tested compounds stock solution (10 mM) was diluted with the ITC buffer to a concentration ratio of 35 μM versus 350 μM protein for ERα, and 20 μM versus 200 μM protein for VCB before using a reverse titration mode. Titrations were performed using an initial injection of 0.4 μL followed by 14 identical injections of 2.5 μL with a duration of 5 seconds per injection and a spacing of 150 seconds between injections. Data were processed with PEAQ-ITC analysis software. Additional background experiments where buffer was titrated into protein solution revealed no significant shift in the baseline during the course of the measurements.

#### TMT-labeled mass spectrometry analysis

Approximately 107 T47D cells were treated with different compounds for 24 h. Cells were lysed using Lysis buffer (4%SDS, 100 mM Tris, PH 7.6) by sonication. Peptides were prepared using Filter Aided Sample Preparation (FASP) protocol. The methods of TMT-labeled peptides and later Data analysis was the same as the reference (J. Med. Chem. 2019, 62, 9281 − 9298). Data were duplicated and analyzed using the two-tailed Student *t* test. Significant changed proteins were labeled using different colors. Downregulated proteins were proteins with Fold change PROTAC 8 / PROTAC 8N < −0.8 and *p*-value <0.01.

### Reporting summary

Further information on research design is available in the Nature Portfolio Reporting Summary linked to this article.

## Supplementary information

Supplementary Information

Reporting Summary

## Data Availability

The PROTACs data used in this study are available in the public database of PROTAC-DB (http://cadd.zju.edu.cn/protacdb/)^65^. Gel source images for Fig. 6 and Supplementary Fig. 3b are included in Supplementary Figs. 7 and 8. The remaining data or questions that regarding this study are available from the corresponding author upon request (Fang Bai: baifang@shanghaitech.edu.cn). Source data are provided with this paper.

## Source data

Source Data

## Code availability

The source code of DeepPROTACs and associated data preparation scripts are available at github (https://github.com/fenglei104/DeepPROTACs)^98^. The final DeepPROTACs model is also provided.
