# Supplementary material for: DeepPROTACs is a deep learning-based targeted degradation predictor for PROTACs
Source: Nat Commun. 2022 Nov 21;13:7133. doi: 10.1038/s41467-022-34807-3 (PMC9681730; doi:10.1038/s41467-022-34807-3)
Supplement: Supplementary file 1 — Supplementary Information [file 41467_2022_34807_MOESM1_ESM.pdf]

Supplementary Information

DeepPROTACs - a deep learning-based targeted degradation predictor for PROTACs

Fenglei Li[a,b,1], Qiaoyu Hu[a,1], Xianglei Zhang [a,1], Renhong Sun [c,1], Zhuanghua Liu [b,1], Sanan Wu[a], Siyuan Tian[a,d], Xinyue Ma [a,b], Zhizhuo Dai[d], Xiaobao Yang[c,]*, Shenghua Gao[b,]*, and Fang Bai[a,b,d,e]*

[a]*Shanghai Institute for Advanced Immunochemical Studies, ShanghaiTech University, 393 Middle Huaxia Road, Shanghai 201210, China*

[b]*School of Information Science and Technology, ShanghaiTech University, 393 Middle Huaxia Road, Shanghai 201210, China*

[c]*Gluetacs Therapeutics (Shanghai) Co., Ltd., 99 Haike Road, Zhangjiang Hi-Tech Park, Shanghai 201210, China*

[d]*School of Life Science and Technology, ShanghaiTech University, 393 Middle Huaxia Road, Shanghai 201210, China*

[e]*Shanghai Clinical Research and Trial Center, Shanghai 201210, China.*

[1]*These authors contributed equally*

* To whom correspondence should be addressed to

Fang Bai: baifang@shanghaitech.edu.cn,

or Shenghua Gao: gaoshh@shanghaitech.edu.cn,

or Xiaobao Yang: yang.xiaobao@gluetacs.com.

Contents of Supporting Information

Contents: Page

Synthesis of toremifene derivative	S3-4
General procedure for the synthesis of ER PROTACs	S5
Characterization data of ER PROTACs	S6-16
Synthesis and characterization of PROTAC 8N	S17
Supplementary Tables	S18-S25
Supplementary Figures	S26-S55

1. Synthesis of toremifene derivative.

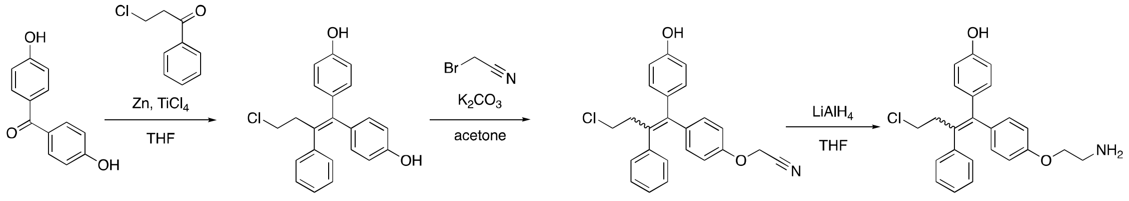

toremifene derivative

Step 1: The preparation of 4,4'-(4-chloro-2-phenylbut-1-ene-1,1-diyl)diphenol

A suspension of Zinc (6.5 g, 100 mmol) in THF was purged and refilled with N_2 for three times. Then $TiCl_4$ (9.5 g, 50 mmol) was added dropwise at 0°C. After the addition was completed, the resulting mixture was refluxed for 2 h, followed by the addition of the solution of bis(4-hydroxyphenyl)methanone (2.14 g, 10 mmol) and 3-chloro-1-phenylpropan-1-one (5.1 g, 30 mmol) in THF (80 mL). Then the mixture was refluxed in the dark for another 3 h. TLC showed the reaction was complete. The reaction mixture was cooled to room temperature, concentrated in vacuo to remove most of the solvent, and then quenched with sat. NH_4Cl aq. and extracted with ethyl acetate. The combined organic layers were washed with water and brine, dried over anhydrous Na_2SO_4, filtered and concentrated in vacuo. The residue was purified by flash column chromatography (eluent: with 0%-30% (v1: v2) ethyl acetate in hexane) to afford the desired product as a yellow solid (3.0 g, 86% yield). ^{1}H NMR (500 MHz, $CDCl_3$) δ 7.21 – 7.10 (m, 7H), 6.84 – 6.81 (m, 2H), 6.75 – 6.72 (m, 2H), 6.49 – 6.46 (m, 2H), 4.99 (s, 1H), 4.73 (s, 1H), 3.45 – 3.36 (m, 2H), 2.99 – 2.91 (m, 2H). HRMS (ESI) m/z: calcd for $C_{22}H_{20}ClO_2^+$ $[M + H]^+$, 351.1146; found, 351.1138.

Step 2: The preparation of 2-(4-(4-chloro-1-(4-hydroxyphenyl)-2-phenylbut-1-en-1-yl)phenoxy)acetonitrile

A solution of 4,4'-(4-chloro-2-phenylbut-1-ene-1,1-diyl)diphenol (1.5 g, 4.28 mmol), K_2CO_3 (592 mg, 4.28 mmol) and 2-bromoacetonitrile (257 mg, 2.14 mmol) in acetone was purged and refilled with N_2 for three times, then was refluxed for 3.5 h. TLC showed the reaction was complete. The reaction mixture was cooled to room temperature and

concentrated in vacuo. The residue was purified by flash column chromatography (eluent: DCM) to afford the desired product as a light yellow oil (782 mg, 94% yield). ^{1}H NMR (500 MHz, $CDCl_3$) δ 7.31 – 7.27 (m, 1H), 7.21-7.18 (m, 2H), 7.17 – 7.14 (m, 2H), 7.13-7.10 (m, 2H), 7.00 – 6.97 (m, 1H), 6.86-6.83 (m, 2H), 6.75 – 6.70 (m, 1H), 6.65 – 6.61 (m, 1H), 6.51 – 6.47 (m, 1H), 4.81 (s, 1H), 4.64 (s, 1H), 3.45 – 3.39 (m, 2H), 2.97-2.91 (m, 2H). HRMS (ESI) m/z: calcd for $C_{24}H_{21}ClNO_2^+$ [M + H]$^+$, 390.1255; found, 390.1263.

Step 3: The preparation of toremifene derivative 4-(1-(4-(2-aminoethoxy)phenyl)-4-chloro-2-phenylbut-1-en-1-yl)phenol

To a solution of 2-(4-(4-chloro-1-(4-hydroxyphenyl)-2-phenylbut-1-en-1-yl)phenoxy)acetonitrile (782 mg, 2 mmol) in THF (25 mL) cooled to 0°C was added $LiAlH_4$ (228 mg, 6 mmol) in portions. The resulting mixture was purged and refilled with N_2 for three times, then stirred at room temperature overnight. After the reaction was complete, the reaction mixture was quenched with sat. NH_4Cl aq. and concentrated in vacuo. The residue was dissolved with MeOH and filtered. The filtrate was concentrated and purified by reverse ISCO (eluent: with 10%-100% (v1: v2) acetonitrile in water (containing 0.05% HCl)) to afford the desired product as a light yellow solid (473 mg, 60% yield). ^{1}H NMR (500 MHz, DMSO-d_6) δ 9.68 – 9.17 (m, 1H), 8.12 (d, J = 41.4 Hz, 3H), 7.24 – 7.18 (m, 3H), 7.16 – 7.12 (m, 3H), 7.06 (d, J = 8.5 Hz, 1H), 7.00 (d, J = 8.7 Hz, 1H), 6.77 (t, J = 8.4 Hz, 2H), 6.65 (d, J = 8.8 Hz, 1H), 6.61 (d, J = 8.6 Hz, 1H), 6.42 (d, J = 8.6 Hz, 1H), 4.20 (t, J = 4.9 Hz, 1H), 4.03 (t, J = 4.9 Hz, 1H), 3.43 (t, J = 7.3 Hz, 2H), 3.23 (s, 1H), 3.12 (s, 1H), 2.93 – 2.83 (m, 2H). HRMS (ESI) m/z: calcd for $C_{24}H_{25}ClNO_2^+$ [M + H]$^+$, 394.1568; found, 394.1561.

2. General procedure for the synthesis of ER PROTACs.

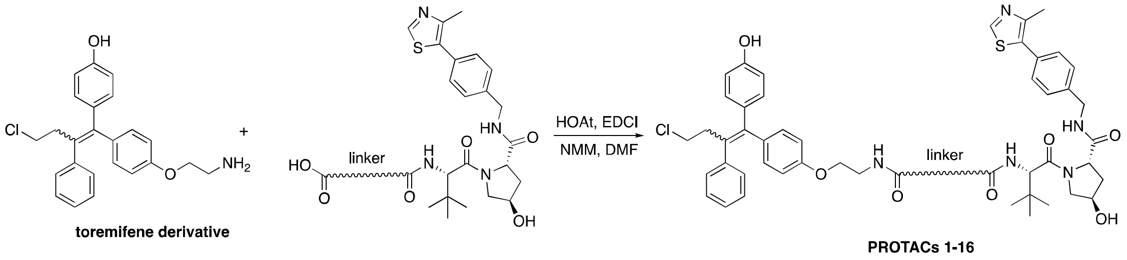

A mixture of toremifene derivative (1.0 eq.), VHL-1 based acid (1.0 eq.), HOAt (2.0 eq.), EDCI (2.0 eq.), NMM (5.0 equiv) and DMF was stirred at room temperature overnight. LC-MS showed the reaction was complete. The reaction mixture was filtered and purified by prep-HPLC (eluent: with 10%-100% (v1: v2) acetonitrile in water (containing 0.05% HCl)). The collections were concentrated in vacuo to remove most of the solvent and then lyophilized to afford the desired PROTAC.

3. Characterization data of ER PROTACs.

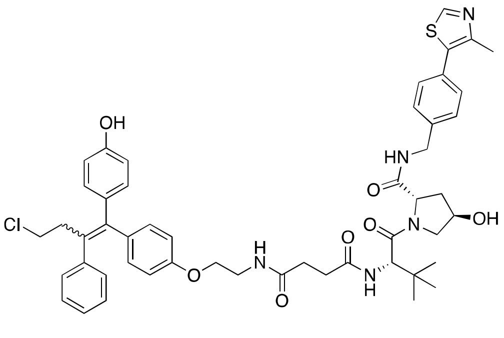

N1-(2-(4-(4-chloro-1-(4-hydroxyphenyl)-2-phenylbut-1-en-1-yl)phenoxy)ethyl)-N4-((S)-1-((2S,4R)-4-hydroxy-2-((4-(4-methylthiazol-5-yl)benzyl)carbamoyl)pyrro-lidin-1-yl)-3,3-dimethyl-1-oxobutan-2-yl)succinamide (PROTAC 1). (white solid, 6.8 mg, 30% yield) ^{1}H NMR (500 MHz, Methanol-d_4) δ 8.98 (s, 1H), 7.47 (d, J = 8.3 Hz, 2H), 7.44-7.41 (m, 2H), 7.23 – 7.06 (m, 7H), 6.95 (d, J = 8.7 Hz, 1H), 6.79-6.76 (m, 2H), 6.68 – 6.64 (m, 1H), 6.57 (d, J = 8.8 Hz, 1H), 6.43 – 6.38 (m, 1H), 4.62 – 4.44 (m, 4H), 4.35 (d, J = 15.5 Hz, 1H), 4.07 (t, J = 5.5 Hz, 1H), 3.90-3.85 (m, 2H), 3.79 – 3.72 (m, 1H), 3.60-3.56 (m, 1H), 3.48-3.45 (m, 1H), 3.42-3.39 (m, 2H), 2.97 – 2.88 (m, 2H), 2.65 – 2.42 (m, 7H), 2.22-2.19 (m, 1H), 2.12 – 2.02 (m, 1H), 1.02-0.99 (m, 9H). HRMS (ESI) m/z: calcd for $C_{50}H_{57}ClN_5O_7S^+$ [M + H]$^+$, 906.3662; found, 906.3672.

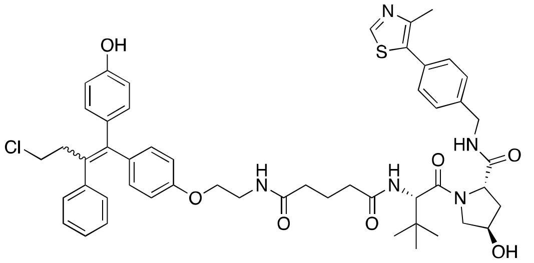

N1-(2-(4-(4-chloro-1-(4-hydroxyphenyl)-2-phenylbut-1-en-1-yl)phenoxy)ethyl)-N5-((S)-1-((2S,4R)-4-hydroxy-2-((4-(4-methylthiazol-5-yl)benzyl)carbamoyl)pyrro-lidin-1-yl)-3,3-dimethyl-1-oxobutan-2-yl)glutaramide (PROTAC 2). (white solid, 5.2 mg, 22% yield) ^{1}H NMR (500 MHz, Methanol-d_4) δ 9.47 (d, J = 5.4 Hz, 1H), 7.52 (d, J = 7.3 Hz,

2H), 7.48 – 7.44 (m, 2H), 7.20 – 7.06 (m, 7H), 6.94 (d, *J* = 8.7 Hz, 1H), 6.80 – 6.74 (m, 2H), 6.68 – 6.63 (m, 1H), 6.58 – 6.53 (m, 1H), 6.43 – 6.37 (m, 1H), 4.61 – 4.47 (m, 4H), 4.35 (dd, *J* = 14.9, 7.1 Hz, 1H), 4.08 (t, *J* = 5.5 Hz, 1H), 3.95 – 3.88 (m, 2H), 3.82 – 3.76 (m, 1H), 3.61-3.57 (m, 1H), 3.52 – 3.43 (m, 1H), 3.42-3.38 (m, 2H), 2.94 – 2.89 (m, 2H), 2.56 – 2.49 (m, 3H), 2.34 – 2.16 (m, 5H), 2.11 – 2.04 (m, 1H), 1.96 – 1.82 (m, 2H), 1.03-0.99 (m, 9H). HRMS (ESI) m/z: calcd for $C_{51}H_{59}ClN_5O_7S^+$ $[M + H]^+$, 920.3818; found, 920.3811.

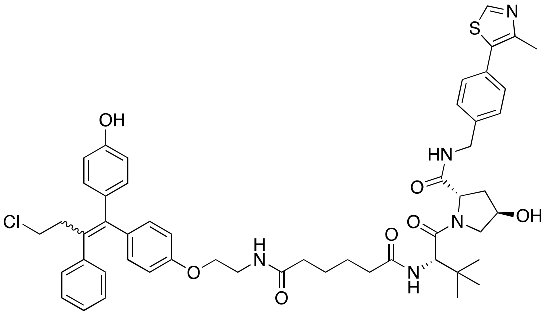

N1-(2-(4-(4-chloro-1-(4-hydroxyphenyl)-2-phenylbut-1-en-1-yl)phenoxy)ethyl)-N6-((S)-1-((2S,4R)-4-hydroxy-2-((4-(4-methylthiazol-5-yl)benzyl)carbamoyl)pyrro-lidin-1-yl)-3,3-dimethyl-1-oxobutan-2-yl)adipamide (PROTAC 3). (white solid, 7.5 mg, 32% yield) ^1H NMR (500 MHz, Methanol-*d*$_4$) δ 9.40 (d, *J* = 5.5 Hz, 1H), 7.54 – 7.49 (m, 2H), 7.46 (d, *J* = 8.3 Hz, 2H), 7.21 – 7.05 (m, 7H), 6.95 (d, *J* = 8.7 Hz, 1H), 6.77 (dd, *J* = 8.6, 6.6 Hz, 2H), 6.67-6.65 (m, 1H), 6.57 (d, *J* = 8.8 Hz, 1H), 6.41 (d, *J* = 8.6 Hz, 1H), 4.65 – 4.45 (m, 4H), 4.37 (d, *J* = 15.6 Hz, 1H), 4.07 (t, *J* = 5.4 Hz, 1H), 3.90 (t, *J* = 5.4 Hz, 2H), 3.82 – 3.72 (m, 1H), 3.58 (t, *J* = 5.5 Hz, 1H), 3.46 (dd, *J* = 10.8, 5.2 Hz, 1H), 3.40 (t, *J* = 7.5 Hz, 2H), 2.95-2.88 (m, 2H), 2.52 (d, *J* = 5.3 Hz, 3H), 2.37 – 2.14 (m, 5H), 2.12 – 2.02 (m, 1H), 1.61 (d, *J* = 20.4 Hz, 4H), 1.02-1.00 (m, 9H). HRMS (ESI) m/z: calcd for $C_{52}H_{61}ClN_5O_7S^+$ $[M + H]^+$, 934.3975; found, 934.3975.

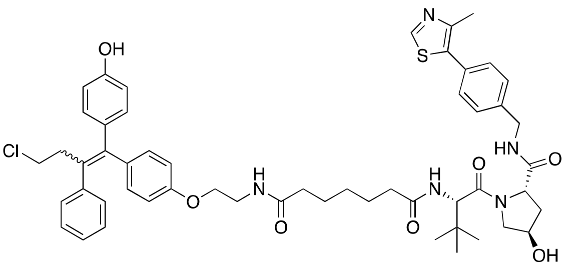

N1-(2-(4-(4-chloro-1-(4-hydroxyphenyl)-2-phenylbut-1-en-1-yl)phenoxy)ethyl)-N7-((S)-1-((2S,4R)-4-hydroxy-2-((4-(4-methylthiazol-5-yl)benzyl)carbamoyl)pyrro-lidin-1-yl)-3,3-dimethyl-1-oxobutan-2-yl)heptanediamide (PROTAC 4). (white solid, 8.1 mg, 34% yield) ^{1}H NMR (500 MHz, Methanol-d_4) δ 8.95 (s, 1H), 7.47 (d, J = 8.3 Hz, 2H), 7.43 – 7.39 (m, 2H), 7.21 – 7.06 (m, 7H), 6.95 (d, J = 8.7 Hz, 1H), 6.80 – 6.75 (m, 2H), 6.68 – 6.64 (m, 1H), 6.59 – 6.55 (m, 1H), 6.43 – 6.39 (m, 1H), 4.62 (d, J = 2.5 Hz, 1H), 4.58-4.49 (m, 3H), 4.35 (d, J = 15.5 Hz, 1H), 4.07 (t, J = 5.5 Hz, 1H), 3.93 – 3.87 (m, 2H), 3.81 – 3.76 (m, 1H), 3.60 – 3.55 (m, 1H), 3.46 (t, J = 5.6 Hz, 1H), 3.41-3.38 (m, 2H), 2.95-2.86 (m, 2H), 2.47 (s, 3H), 2.29-2.15 (m Hz, 5H), 2.11-2.03 (m, 1H), 1.68 – 1.54 (m, 4H), 1.38 – 1.32 (m, 2H), 1.02-1.00 (m, 9H). HRMS (ESI) m/z: calcd for $C_{53}H_{63}ClN_5O_7S^+$ [M + H]$^+$, 948.4131; found, 948.4137.

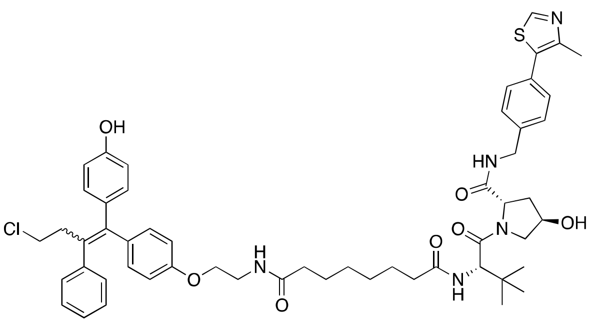

N1-(2-(4-(4-chloro-1-(4-hydroxyphenyl)-2-phenylbut-1-en-1-yl)phenoxy)ethyl)-N8-((S)-1-((2S,4R)-4-hydroxy-2-((4-(4-methylthiazol-5-yl)benzyl)carbamoyl)pyrro-lidin-1-yl)-3,3-dimethyl-1-oxobutan-2-yl)octanediamide (PROTAC 5). (white solid, 8.4 mg, 34% yield) ^{1}H NMR (500 MHz, Methanol-d_4) δ 9.20 (s, 1H), 7.49 (d, J = 8.1 Hz, 2H), 7.46-7.43 (m, 2H), 7.21 – 7.05 (m, 7H), 6.95-6.91 (m, 1H), 6.79-6.76 (m, 2H), 6.68 – 6.62

(m, 1H), 6.57-6.53 (m, 1H), 6.43 – 6.37 (m, 1H), 4.63 (s, 1H), 4.60 – 4.47 (m, 3H), 4.39 – 4.32 (m, 1H), 4.08-4.05 (m, 1H), 3.95 – 3.85 (m, 2H), 3.79 (dd, *J* = 11.0, 3.8 Hz, 1H), 3.58-3.54 (m, 1H), 3.46 (t, *J* = 5.4 Hz, 1H), 3.40 (t, *J* = 7.4 Hz, 2H), 2.95-2.88 (m, 2H), 2.50 (s, 3H), 2.30 – 2.13 (m, 5H), 2.10-2.03 (m, 1H), 1.61-1.57 (m, 4H), 1.36 – 1.27 (m, 4H), 1.03-1.00 (m, 9H). HRMS (ESI) m/z: calcd for $C_{54}H_{65}ClN_5O_7S^+$ $[M + H]^+$, 962.4288; found, 962.4280.

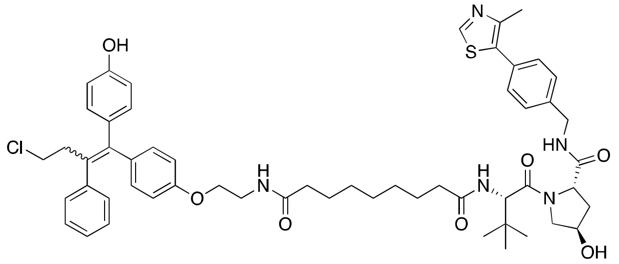

N1-(2-(4-(4-chloro-1-(4-hydroxyphenyl)-2-phenylbut-1-en-1-yl)phenoxy)ethyl)-N9-((S)-1-((2S,4R)-4-hydroxy-2-((4-(4-methylthiazol-5-yl)benzyl)carbamoyl)pyrro-lidin-1-yl)-3,3-dimethyl-1-oxobutan-2-yl)nonanediamide (PROTAC 6). (white solid, 9.7 mg, 40% yield) ^{1}H NMR (500 MHz, Methanol-d_4) δ 9.31 (s, 1H), 7.51 (d, *J* = 8.2 Hz, 2H), 7.48 – 7.43 (m, 2H), 7.21 – 7.06 (m, 7H), 6.96 – 6.91 (m, 1H), 6.80 – 6.74 (m, 2H), 6.69 – 6.64 (m, 1H), 6.58 – 6.53 (m, 1H), 6.43 – 6.38 (m, 1H), 4.63 (s, 1H), 4.61 – 4.46 (m, 3H), 4.37 (d, *J* = 15.6 Hz, 1H), 4.06 (t, *J* = 5.4 Hz, 1H), 3.92-3.88 (m, 2H), 3.80 (dd, *J* = 10.9, 3.8 Hz, 1H), 3.56 (t, *J* = 5.4 Hz, 1H), 3.46 (t, *J* = 5.4 Hz, 1H), 3.40 (t, *J* = 7.4 Hz, 2H), 2.95-2.89 (m, 2H), 2.52 (s, 3H), 2.32 – 2.12 (m, 5H), 2.10-2.02 (m, 1H), 1.67 – 1.51 (m, 4H), 1.37 – 1.23 (m, 6H), 1.03-1.00 (m, 9H). HRMS (ESI) m/z: calcd for $C_{55}H_{67}ClN_5O_7S^+$ $[M + H]^+$, 976.4444; found, 976.4441.

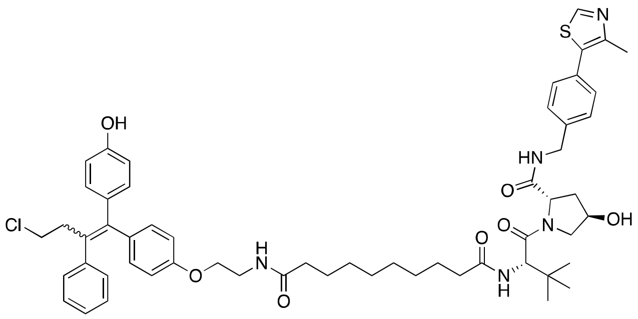

N1-(2-(4-(4-chloro-1-(4-hydroxyphenyl)-2-phenylbut-1-en-1-yl)phenoxy)ethyl)-N10-((S)-1-((2S,4R)-4-hydroxy-2-((4-(4-methylthiazol-5-yl)benzyl)carbamoyl)pyrro-lidin-1-yl)-3,3-dimethyl-1-oxobutan-2-yl)decanediamide (PROTAC 7). (white solid, 9.0 mg, 36% yield) ^{1}H NMR (500 MHz, Methanol-d_4) δ 9.23 (s, 1H), 7.50 (d, J = 8.3 Hz, 2H), 7.44 (d, J = 8.3 Hz, 2H), 7.22 – 7.06 (m, 7H), 6.96 – 6.91 (m, 1H), 6.82 – 6.74 (m, 2H), 6.68 – 6.63 (m, 1H), 6.58 – 6.53 (m, 1H), 6.44 – 6.37 (m, 1H), 4.63 (s, 1H), 4.60 – 4.47 (m, 3H), 4.36 (d, J = 15.6 Hz, 1H), 4.07 (t, J = 5.4 Hz, 1H), 3.89 (t, J = 9.4 Hz, 2H), 3.80 (dd, J = 11.0, 3.8 Hz, 1H), 3.57 (t, J = 5.4 Hz, 1H), 3.46 (t, J = 5.4 Hz, 1H), 3.40 (t, J = 7.4 Hz, 2H), 2.95-2.89 (m, 2H), 2.51 (s, 3H), 2.31 – 2.12 (m, 5H), 2.10-2.04 (m, 1H), 1.65 – 1.52 (m, 4H), 1.29-1.27 (m, 8H), 1.03-1.01 (m, 9H). HRMS (ESI) m/z: calcd for $C_{56}H_{69}ClN_5O_7S^+$ $[M + H]^+$, 990.4601; found, 990.4611.

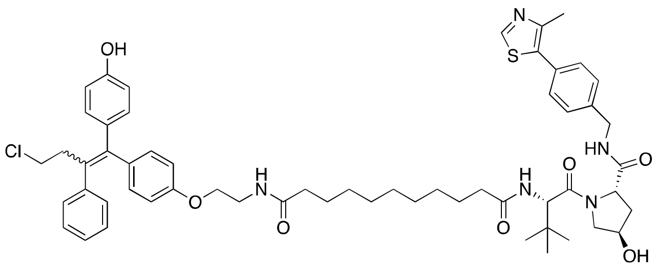

N1-(2-(4-(4-chloro-1-(4-hydroxyphenyl)-2-phenylbut-1-en-1-yl)phenoxy)ethyl)-N11-((S)-1-((2S,4R)-4-hydroxy-2-((4-(4-methylthiazol-5-yl)benzyl)carbamoyl)pyrro-lidin-1-yl)-3,3-dimethyl-1-oxobutan-2-yl)undecanediamide (PROTAC 8). (white solid, 9.6 mg, 38% yield) ^{1}H NMR (500 MHz, Methanol-d_4) δ 9.15 (s, 1H), 7.49 (d, J = 8.3 Hz, 2H), 7.46-7.43 (m, 2H), 7.22 – 7.05 (m, 7H), 6.94 (d, J = 8.7 Hz, 1H), 6.78 (dd, J = 9.6, 8.8 Hz,

2H), 6.66 (d, *J* = 8.7 Hz, 1H), 6.60 – 6.52 (m, 1H), 6.45 – 6.38 (m, 1H), 4.63 (s, 1H), 4.60 – 4.47 (m, 3H), 4.36 (d, *J* = 15.6 Hz, 1H), 4.07 (t, *J* = 5.4 Hz, 1H), 3.92-3.88 (m, 2H), 3.80 (dd, *J* = 10.5, 3.2 Hz, 1H), 3.58-3.55(m, 1H), 3.46 (t, *J* = 5.4 Hz, 1H), 3.40 (t, *J* = 7.4 Hz, 2H), 2.92 (dt, *J* = 11.9, 7.5 Hz, 2H), 2.50 (s, 3H), 2.32 – 2.12 (m, 5H), 2.10-2.05 (m, 1H), 1.59-1.57 (m, 4H), 1.28 (d, *J* = 9.2 Hz, 10H), 1.03-1.01 (m, 9H). HRMS (ESI) m/z: calcd for $C_{57}H_{71}ClN_5O_7S^+$ [M + H]$^+$, 1004.4757; found, 1004.4761.

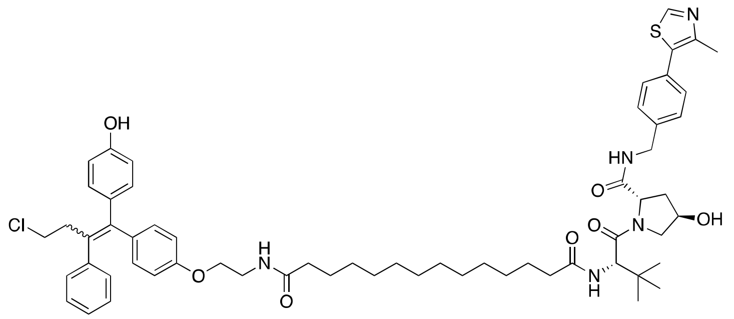

N1-(2-(4-(4-chloro-1-(4-hydroxyphenyl)-2-phenylbut-1-en-1-yl)phenoxy)ethyl)-N14-((S)-1-((2S,4R)-4-hydroxy-2-((4-(4-methylthiazol-5-yl)benzyl)carbamoyl)pyrro-lidin-1-yl)-3,3-dimethyl-1-oxobutan-2-yl)tetradecanediamide (PROTAC 9). (white solid, 10.2 mg, 38% yield) ^{1}H NMR (500 MHz, Methanol-*d*$_4$) δ 9.23 (s, 1H), 7.50 (d, *J* = 8.2 Hz, 2H), 7.47-7.44 (m, 2H), 7.22 – 7.15 (m, 3H), 7.15 – 7.08 (m, 4H), 6.97 – 6.91 (m, 1H), 6.81 – 6.74 (m, 2H), 6.66 (d, *J* = 8.7 Hz, 1H), 6.58-6.55 (m, 1H), 6.41 (d, *J* = 8.6 Hz, 1H), 4.63 (s, 1H), 4.61 – 4.46 (m, 3H), 4.36 (d, *J* = 15.6 Hz, 1H), 4.07 (t, *J* = 5.4 Hz, 1H), 3.92-3.89 (m, 2H), 3.80 (dd, *J* = 11.0, 3.8 Hz, 1H), 3.58-3.55 (m, 1H), 3.47 (t, *J* = 5.4 Hz, 1H), 3.40 (t, *J* = 7.4 Hz, 2H), 2.92 (dt, *J* = 11.9, 7.5 Hz, 2H), 2.51 (s, 3H), 2.31-2.22 (m, 5H), 2.21-2.03 (m, 1H), 1.63 – 1.54 (m, 4H), 1.28 (d, *J* = 13.1 Hz, 16H), 1.11 – 0.85 (m, 9H). HRMS (ESI) m/z: calcd for $C_{60}H_{77}ClN_5O_7S^+$ [M + H]$^+$, 1046.5227; found, 1046.5224.

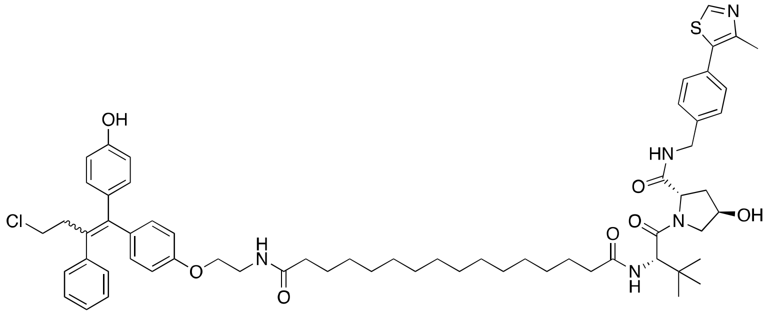

N1-(2-(4-(4-chloro-1-(4-hydroxyphenyl)-2-phenylbut-1-en-1-yl)phenoxy)ethyl)-N16-((S)-1-((2S,4R)-4-hydroxy-2-((4-(4-methylthiazol-5-yl)benzyl)carbamoyl)pyrro-lidin-1-yl)-3,3-dimethyl-1-oxobutan-2-yl)hexadecanediamide (PROTAC 10). (white solid, 12.1 mg, 44% yield) ^{1}H NMR (500 MHz, Methanol-*d*$_4$) δ 9.01 (s, 1H), 7.48 (d, *J* = 8.2 Hz, 2H), 7.45-7.42 (m, 2H), 7.23 – 7.07 (m, 7H), 6.94 (d, *J* = 8.7 Hz, 1H), 6.82 – 6.74 (m, 2H), 6.66 (d, *J* = 8.7 Hz, 1H), 6.56 (d, *J* = 8.8 Hz, 1H), 6.41 (d, *J* = 8.7 Hz, 1H), 4.63 (s, 1H), 4.58-4.50 (m, 3H), 4.36 (d, *J* = 15.5 Hz, 1H), 4.08 (t, *J* = 5.3 Hz, 1H), 3.92-3.89 (m, 2H), 3.80 (dd, *J* = 10.9, 3.9 Hz, 1H), 3.57 (t, *J* = 5.3 Hz, 1H), 3.48-3.45 (m, 1H), 3.40 (t, *J* = 7.4 Hz, 2H), 2.93 (dt, *J* = 11.9, 7.5 Hz, 2H), 2.49 (s, 3H), 2.32 – 2.13 (m, 5H), 2.11-2.03 (m, 1H), 1.60-1.56 (m, 4H), 1.27 (d, *J* = 14.9 Hz, 20H), 1.03-1.01 (m, 9H). HRMS (ESI) m/z: calcd for $C_{62}H_{81}ClN_5O_7S^+$ $[M + H]^+$, 1074.5540; found, 1074.5539.

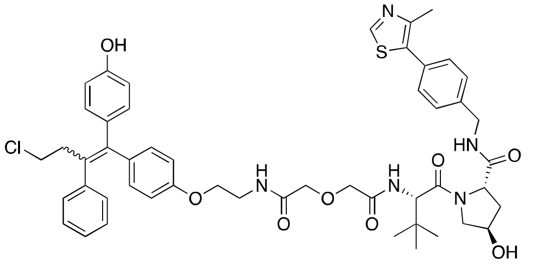

(2S,4R)-1-((S)-2-(2-(2-((2-(4-(4-chloro-1-(4-hydroxyphenyl)-2-phenylbut-1-en-1-yl)phenoxy)ethyl)amino)-2-oxoethoxy)acetamido)-3,3-dimethylbutanoyl)-4-hydroxy-N-(4-(4-methylthiazol-5-yl)benzyl)pyrrolidine-2-carboxamide (PROTAC 11). (white solid, 6.4 mg, 28% yield) ^{1}H NMR (500 MHz, Methanol-*d*$_4$) δ 9.13 – 9.04 (m, 1H), 7.49 (dd, *J* = 8.4, 1.6 Hz, 2H), 7.46 – 7.41 (m, 2H), 7.20 – 7.10 (m, 6H), 7.09 – 7.06 (m, 1H), 6.95 – 6.91 (m, 1H), 6.79 – 6.73 (m, 2H), 6.67 – 6.63 (m, 1H), 6.57 – 6.53 (m, 1H), 6.43 –

6.38 (m, 1H), 4.68 (d, J = 9.2 Hz, 1H), 4.61-4.54 (m, 2H), 4.52-4.48 (m, 1H), 4.32 (dd, J = 15.6, 1.7 Hz, 1H), 4.15 – 4.10 (m, 4H), 4.08-4.06 (m, 1H), 3.96 – 3.86 (m, 2H), 3.83-3.76 (m, 1H), 3.71 – 3.63 (m, 1H), 3.59-3.53 (m, 1H), 3.41-3.36 (m, 2H), 2.94 – 2.88 (m, 2H), 2.48 (d, J = 3.9 Hz, 3H), 2.27-2.19 (m, 1H), 2.13-2.04 (m, 1H), 1.04 – 0.99 (m, 9H). HRMS (ESI) m/z: calcd for $C_{50}H_{57}ClN_5O_8S^+$ $[M + H]^+$, 922.3611; found, 922.3601.

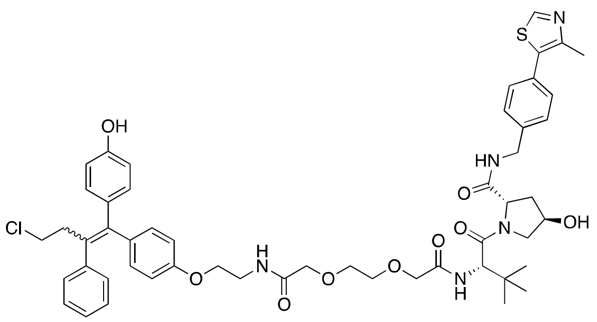

(2S,4R)-1-((S)-2-(tert-butyl)-14-(4-(4-chloro-1-(4-hydroxyphenyl)-2-phenylbut-1-en-1-yl)phenoxy)-4,11-dioxo-6,9-dioxa-3,12-diazatetradecanoyl)-4-hydroxy-N-(4-(4-methylthiazol-5-yl)benzyl)pyrrolidine-2-carboxamide (PROTAC 12). (white solid, 6.3 mg, 26% yield) ^{1}H NMR (500 MHz, Methanol-d_4) δ 9.29 (d, J = 10.0 Hz, 1H), 7.50 – 7.42 (m, 4H), 7.20 – 7.14 (m, 3H), 7.14 – 7.07 (m, 4H), 6.93-6.91 (m, 1H), 6.80 – 6.74 (m, 2H), 6.68 – 6.63 (m, 1H), 6.57-6.54 (m, 1H), 6.42 – 6.36 (m, 1H), 4.70 (d, J = 9.5 Hz, 1H), 4.62 – 4.53 (m, 1H), 4.52-4.46 (m, 2H), 4.41 – 4.33 (m, 1H), 4.10 – 3.99 (m, 5H), 3.94 – 3.85 (m, 2H), 3.81-3.76 (m, 1H), 3.74 – 3.64 (m, 5H), 3.58 – 3.47 (m, 1H), 3.39 (t, J = 7.4 Hz, 2H), 2.94 – 2.89 (m, 2H), 2.52-2.50 (m, 3H), 2.26-2.21 (m, 1H), 2.10-2.04 (m, 1H), 1.02-0.99 (m, 9H). HRMS (ESI) m/z: calcd for $C_{52}H_{61}ClN_5O_9S^+$ $[M + H]^+$, 966.3873; found, 966.3879.

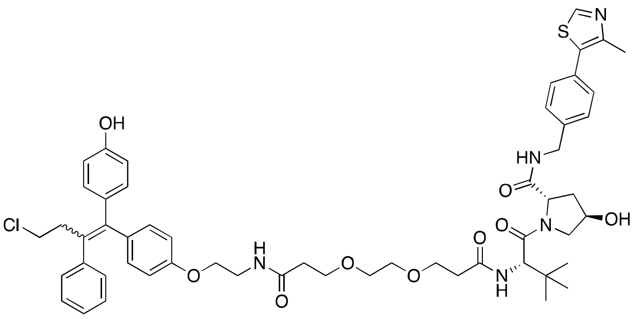

(2S,4R)-1-((S)-2-(tert-butyl)-16-(4-(4-chloro-1-(4-hydroxyphenyl)-2-phenylbut-1-en-1-yl)phenoxy)-4,13-dioxo-7,10-dioxa-3,14-diazahexadecanoyl)-4-hydroxy-N-(4-(4-methylthiazol-5-yl)benzyl)pyrrolidine-2-carboxamide (PROTAC 13). (white solid, 7.5 mg, 30% yield) ^{1}H NMR (500 MHz, Methanol-d_4) δ 9.31 (s, 1H), 7.57 – 7.42 (m, 4H), 7.20-7.15 (m, 3H), 7.15 – 7.07 (m, 4H), 6.97 – 6.91 (m, 1H), 6.81 – 6.74 (m, 2H), 6.69 – 6.64 (m, 1H), 6.56 (d, *J* = 8.8 Hz, 1H), 6.44 – 6.38 (m, 1H), 4.64 (d, *J* = 4.3 Hz, 1H), 4.60-4.49 (m, 3H), 4.42 – 4.32 (m, 1H), 4.07 (t, *J* = 5.4 Hz, 1H), 3.90-3.88 (m, 2H), 3.82 – 3.76 (m, 1H), 3.73 (t, *J* = 6.0 Hz, 1H), 3.70-3.63 (m, 3H), 3.61 – 3.51 (m, 5H), 3.49 (t, *J* = 5.2 Hz, 1H), 3.39 (t, *J* = 7.4 Hz, 2H), 2.91 (dt, *J* = 13.1, 7.4 Hz, 2H), 2.55 – 2.40 (m, 7H), 2.27 – 2.18 (m, 1H), 2.12 – 2.00 (m, 1H), 1.03-1.01 (m, 9H). HRMS (ESI) m/z: calcd for $C_{54}H_{65}ClN_5O_9S^+$ [M + H]$^+$, 994.4186; found, 994.4196.

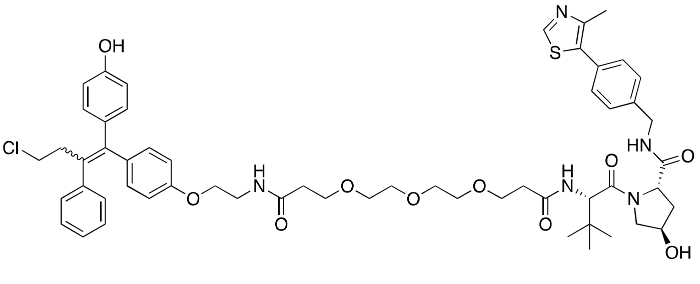

(2S,4R)-1-((S)-2-(tert-butyl)-19-(4-(4-chloro-1-(4-hydroxyphenyl)-2-phenylbut-1-en-1-yl)phenoxy)-4,16-dioxo-7,10,13-trioxa-3,17-diazanonadecanoyl)-4-hydroxy-N-(4-(4-methylthiazol-5-yl)benzyl)pyrrolidine-2-carboxamide (PROTAC 14). (white solid, 7.2 mg, 27% yield) ^{1}H NMR (500 MHz, Methanol-d_4) δ 9.17 (d, *J* = 1.8 Hz, 1H), 7.49 (d, *J* = 8.0 Hz, 2H), 7.46 – 7.41 (m, 2H), 7.22 – 7.15 (m, 3H), 7.15 – 7.07 (m, 4H), 6.97 – 6.92

(m, 1H), 6.81 – 6.74 (m, 2H), 6.69 – 6.64 (m, 1H), 6.58-6.56 (m, 1H), 6.44 – 6.38 (m, 1H), 4.64 (s, 1H), 4.58-4.49 (m, 3H), 4.39-4.34 (m, 1H), 4.08 (t, *J* = 5.4 Hz, 1H), 3.91-3.88 (m, 2H), 3.79 (dd, *J* = 11.0, 3.8 Hz, 1H), 3.75 – 3.66 (m, 4H), 3.60-3.53 (m, 9H), 3.48 (t, *J* = 5.4 Hz, 1H), 3.40 (t, *J* = 7.4 Hz, 2H), 2.92 (dt, *J* = 11.9, 7.4 Hz, 2H), 2.60 – 2.39 (m, 7H), 2.24-2.20 (m, 1H), 2.10-2.05 (m, 1H), 1.03-1.01 (m, 9H). HRMS (ESI) m/z: calcd for $C_{56}H_{69}ClN_5O_{10}S^+$ $[M + H]^+$, 1038.4448; found, 1038.4442.

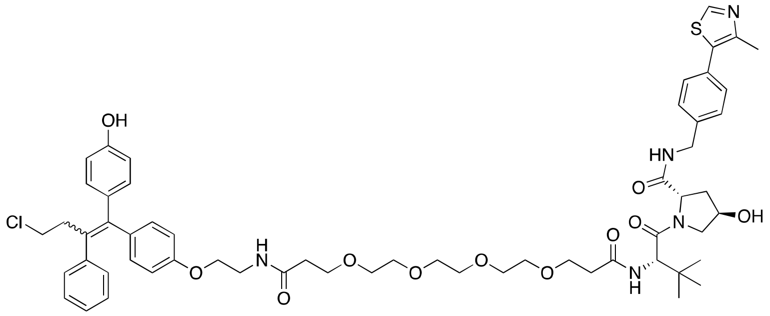

N1-(2-(4-(4-chloro-1-(4-hydroxyphenyl)-2-phenylbut-1-en-1-yl)phenoxy)ethyl)-N16-((S)-1-((2S,4R)-4-hydroxy-2-((4-(4-methylthiazol-5-yl)benzyl)carbamoyl)pyrro-lidin-1-yl)-3,3-dimethyl-1-oxobutan-2-yl)-4,7,10,13-tetraoxahexadecane-diamide (PROTAC 15). (white solid, 7.9 mg, 29% yield) ^{1}H NMR (500 MHz, Methanol-d_4) δ 9.23 (d, *J* = 1.3 Hz, 1H), 7.50 (d, *J* = 8.2 Hz, 2H), 7.44 (d, *J* = 8.1 Hz, 2H), 7.21 – 7.15 (m, 3H), 7.15 – 7.07 (m, 4H), 6.95 (d, *J* = 8.7 Hz, 1H), 6.81 – 6.75 (m, 2H), 6.69 – 6.64 (m, 1H), 6.57 (d, *J* = 8.8 Hz, 1H), 6.44 – 6.38 (m, 1H), 4.64 (s, 1H), 4.60 – 4.47 (m, 3H), 4.39 – 4.34 (m, 1H), 4.09-4.06 (m, 1H), 3.91-3.88 (m, 2H), 3.79 (dd, *J* = 11.0, 3.8 Hz, 1H), 3.74-3.67 (m Hz, 4H), 3.60-3.51 (m, 11H), 3.54 – 3.52 (m, 2H), 3.49 (t, *J* = 5.4 Hz, 1H), 3.40 (t, *J* = 7.4 Hz, 2H), 2.95 – 2.88 (m, 2H), 2.60 – 2.46 (m, 6H), 2.42 (t, *J* = 6.0 Hz, 1H), 2.24-2.20 (m, 1H), 2.10-2.03 (m, 1H), 1.03-1.01 (m, 9H). HRMS (ESI) m/z: calcd for $C_{58}H_{73}ClN_5O_{11}S^+$ $[M + H]^+$, 1082.4710; found, 1082.4706.

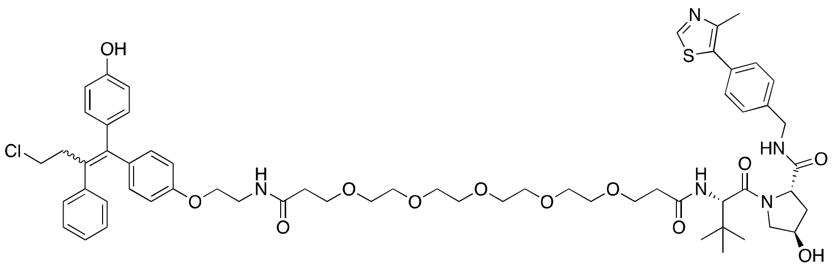

N1-(2-(4-(4-chloro-1-(4-hydroxyphenyl)-2-phenylbut-1-en-1-yl)phenoxy)ethyl)-N19-((S)-1-((2S,4R)-4-hydroxy-2-((4-(4-methylthiazol-5-yl)benzyl)carbamoyl)pyrro-lidin-1-yl)-3,3-dimethyl-1-oxobutan-2-yl)-4,7,10,13,16-pentaoxanonadecane-diamide (PROTAC 16). (white solid, 8.1 mg, 28% yield) ^{1}H NMR (500 MHz, Methanol-*d*$_4$) δ 9.63 (d, *J* = 1.1 Hz, 1H), 7.54 (d, *J* = 8.2 Hz, 2H), 7.48 (d, *J* = 8.2 Hz, 2H), 7.22 – 7.15 (m, 3H), 7.15 – 7.07 (m, 4H), 6.97 – 6.94 (m, 1H), 6.82 – 6.75 (m, 2H), 6.68 – 6.64 (m, 1H), 6.60 – 6.55 (m, 1H), 6.43 – 6.39 (m, 1H), 4.64 (s, 1H), 4.60 – 4.47 (m, 3H), 4.41 – 4.34 (m, 1H), 4.08 (t, *J* = 5.4 Hz, 1H), 3.92-3.88 (m, 2H), 3.83 – 3.77 (m, 1H), 3.75 – 3.68 (m, 4H), 3.62 – 3.56 (m, 13H), 3.55-3.54 (m, 4H), 3.49 (t, *J* = 5.4 Hz, 1H), 3.41-3.38 (m, 2H), 2.95 – 2.90 (m, 2H), 2.61 – 2.53 (m, 4H), 2.49-2.42 (m, 3H), 2.25-2.20 (m, 1H), 2.10-2.05 (m, 1H), 1.03-1.01 (m, 9H). HRMS (ESI) m/z: calcd for $C_{60}H_{77}ClN_5O_{12}S^+$ [M + H]$^+$, 1126.4972; found, 1126.4981.

4. Procedure for the synthesis and characterization data of negative control PROTAC 8N.

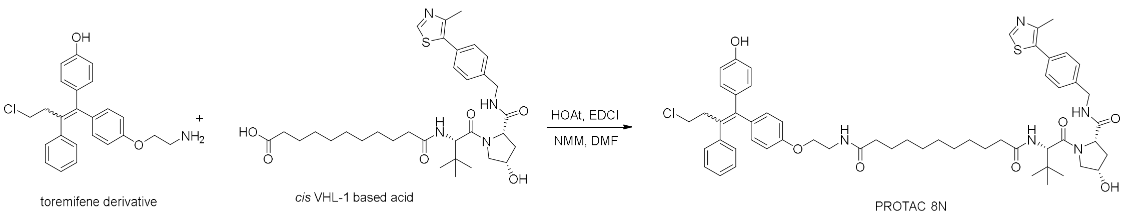

A mixture of toremifene derivative (1.0 eq.), *cis* VHL-1 based acid (1.0 eq.), HOAt (2.0 eq.), EDCI (2.0 eq.), NMM (5.0 eq.) and DMF was stirred at room temperature overnight. LC-MS showed the reaction was complete. The reaction mixture was filtered and purified by prep-HPLC (eluent: with 10%-100% (v1: v2) acetonitrile in water (containing 0.05% HCl)). The collections were concentrated in vacuo to remove most of the solvent and then lyophilized to afford N1-(2-(4-(4-chloro-1-(4-hydroxyphenyl)-2-phenylbut-1-en-1-yl)phenoxy)ethyl)-N11-((S)-1-((2S,4S)-4-hydroxy-2-((4-(4-methylthiazol-5-yl)benzyl)carbamoyl)pyrrolidin-1-yl)-3,3-dimethyl-1-oxobutan-2-yl)undecanediamide (PROTAC 8N). (white solid, 10.6 mg, 38% yield). ^{1}H NMR (500 MHz, DMSO-d_6) δ 9.63 – 9.08 (m, 1H), 8.98 (s, 1H), 8.70 – 8.56 (m, 1H), 8.10 – 7.90 (m, 1H), 7.84 (d, *J* = 8.8 Hz, 1H), 7.39 (q, *J* = 8.4 Hz, 4H), 7.22 – 7.10 (m, 6H), 7.05 (d, *J* = 8.5 Hz, 1H), 6.93 (t, *J* = 9.8 Hz, 1H), 6.80 – 6.67 (m, 2H), 6.64 – 6.55 (m, 2H), 6.45 – 6.34 (m, 1H), 4.52 – 4.39 (m, 2H), 4.36 (dd, *J* = 8.4, 6.2 Hz, 1H), 4.27-4.18 (m, 2H), 4.03 – 3.90 (m, 2H), 3.81 (t, *J* = 5.5 Hz, 1H), 3.31 (d, *J* = 5.5 Hz, 4H), 2.87 (dt, *J* = 10.3, 7.4 Hz, 2H), 2.44 (s, 3H), 2.35-2.29 (m, 1H), 2.23 (dt, *J* = 14.4, 7.4 Hz, 1H), 2.13 – 2.00 (m, 3H), 1.74 (dt, *J* = 12.3, 6.0 Hz, 1H), 1.45 (dd, *J* = 13.3, 6.6 Hz, 4H), 1.20 (d, *J* = 8.2 Hz, 11H), 0.92 (d, *J* = 21.5 Hz, 9H). HRMS (ESI) m/z: calcd for $C_{57}H_{71}ClN_5O_7S^+$ $[M + H]^+$, 1004.4757; found, 1004.4763.

Supplementary Table 1. The prediction results of DeepPROTACs model on test set.

test set	truth=1	truth=0	total
prediction=1	315	74	389
prediction=0	54	124	178
total	369	198	567
test set	truth=1	truth=0	total
prediction=1	55.56%	13.05%	68.61%
prediction=0	9.52%	21.87%	31.39%
total	65.08%	34.92%	100%

Supplementary Table 2. The distribution of active and inactive PROTAC molecules across various target proteins and E3 ligases.

Target_E3	Active	Inactive	Target_E3	Active	Inactive
ABL_CRBN	3	2	ALK_CRBN	10	43
ABL_IAP	1	1	ALK_VHL	12	10
ABL_VHL	1	2	AR_CRBN	16	42
ABL_cIAP1	2	10	AR_MDM2	0	1
AKT1_CRBN	7	10	AR_VHL	46	39
AKT1_VHL	3	8	AR_cIAP1	0	18
AKT2_CRBN	1	12	AURKA_CRBN	4	3
AKT2_VHL	2	16	AURKA_VHL	0	2
ALK-G1202R_CRBN	0	2	Alpha-tubulin_CRBN	0	13
ALK-G1202R_VHL	0	1	BCL-xL_CRBN	24	11
BCL-xL_VHL	9	18	BCR-ABL_VHL	7	28
BCL-xL_XIAP	6	2	BCR-ABL_cIAP1	4	16
BCL-xL_cIAP1	0	8	BRAF-D594N_VHL	3	0
BCL2_CRBN	0	11	BRAF-G466E_VHL	4	0
BCL6_CRBN	1	0	BRAF-G466V_VHL	2	0
BCR-ABL-E255K_CRBN	0	1	BRAF-G469A_VHL	2	1
BCR-ABL-H396R_CRBN	0	1	BRAF-K601E_VHL	1	3
BCR-ABL-T315I_CRBN	0	3	BRAF-V600E_CRBN	7	29
BCR-ABL-V468F_CRBN	0	1	BRAF-V600E_VHL	3	11
BCR-ABL_CRBN	13	24	BRAF-V600K_VHL	0	2
BRAF_CRBN	0	4	BRD4_MDM2	2	0
BRAF_VHL	0	4	BRD4_VHL	25	20
BRD2_CRBN	33	6	BRD4_cIAP1	1	1
BRD2_VHL	5	9	BRD7_DCAF15	0	2
BRD3-BD2_KEAP1	0	3	BRD7_VHL	3	28
BRD3_CRBN	31	8	BRD9_CRBN	9	5
BRD3_VHL	5	7	BRD9_DCAF15	0	2
BRD4-BD1_KEAP1	0	3	BRD9_VHL	8	26
BRD4-BD2_KEAP1	0	3	BRDT_VHL	1	0
BRD4_CRBN	51	22	BTK-C481S_CRBN	4	0
BTK_CRBN	39	55	CDK2_CRBN	14	23
BTK_MDM2	0	4	CDK4_CRBN	17	39
BTK_VHL	2	10	CDK4_MDM2	1	4

BTK_XIAP	0	1	CDK4_VHL	3	9
BTK_cIAP1	0	5	CDK4_cIAP1	2	5
Beta-tubulin_CRBN	0	13	CDK5_CRBN	1	1
CBP_CRBN	1	0	CDK6_CRBN	44	16
CDK12-C1039F_CRBN	2	0	CDK6_MDM2	1	4
CDK12_CRBN	3	5	CDK6_VHL	7	5
CDK1_CRBN	0	2	CDK6_XIAP	0	1
CDK6_cIAP1	2	5	Cdc20_CRBN	0	8
CDK7_CRBN	0	1	Cdc20_VHL	0	7
CDK8_CRBN	0	3	EED_VHL	2	6
CDK9_CRBN	17	88	EGFR-L858R-T790M-C797S_CRBN	0	1
CRABP-II_cIAP1	1	4	EGFR-L858R-T790M-L718Q_CRBN	0	1
CRABP-I_cIAP1	2	1	EGFR-L858R-T790M_CRBN	6	24
CRABP2_cIAP1	0	2	EGFR-L858R-T790M_MDM2	1	4
CRBN_CRBN	2	5	EGFR-L858R-T790M_VHL	3	14
CRBN_VHL	8	19	EGFR-L858R-T790M_cIAP1	0	1
CYP1B1_CRBN	1	3	EGFR-L858R_CRBN	1	0
EGFR-L858R_VHL	2	0	ERRalpha_CRBN	0	1
EGFR-e19d_CRBN	13	2	ERRalpha_VHL	7	4
EGFR-e19d_VHL	3	8	ER_CRBN	12	15
EGFR_CRBN	2	17	ER_IAP	0	2
EGFR_MDM2	0	5	ER_VHL	49	95
EGFR_VHL	4	13	ER_XIAP	4	2
EGFR_cIAP1	0	1	ER_cIAP1	6	18
EP300_CRBN	1	0	EZH2_CRBN	4	14
ERK1_CRBN	0	1	FAK_CRBN	32	0
ERK2_CRBN	0	1	FAK_VHL	12	1
FKBP12-F36V_CRBN	2	0	HDAC1_VHL	0	2
FKBP12-F36V_VHL	1	1	HDAC1_cIAP1	0	1
FKBP12_CRBN	4	6	HDAC2_CRBN	0	8
FLT-3_CRBN	2	7	HDAC2_VHL	0	2
FLT-3_VHL	1	0	HDAC3_CRBN	0	12
GCN5_CRBN	1	0	HDAC3_VHL	3	3
GSK-3beta_CRBN	1	15	HDAC6_CRBN	25	15
GSPT1_CRBN	7	3	HDAC6_VHL	5	7
H-PGDS_CRBN	7	0	HDAC6_cIAP1	0	1
HDAC1_CRBN	0	4	HDAC8_cIAP1	0	1
HMGCR_CRBN	1	39	IRAK4_XIAP	0	2
HPGDS_CRBN	1	0	JAK1_CRBN	0	8

HPGDS_cIAP1	0	1	JAK1_VHL	0	8
HPK1_CRBN	2	2	JAK1_XIAP	0	8
IDO1_CRBN	0	7	JAK2_CRBN	0	8
IGF-1R_CRBN	0	11	JAK2_VHL	0	8
IRAK3_CRBN	4	6	JAK2_XIAP	0	8
IRAK3_VHL	0	8	KRAS-G12C_CRBN	0	16
IRAK4_CRBN	15	19	KRAS-G12C_VHL	0	6
IRAK4_VHL	0	4	KRAS_CRBN	0	2
KRAS_VHL	0	4	NSD3_CRBN	0	2
MCL1_CRBN	0	17	NSD3_VHL	0	4
MDM2_CRBN	32	18	PARP1_CRBN	14	38
MDM2_VHL	0	3	PARP1_MDM2	0	2
MEK1_CRBN	2	40	PARP1_VHL	2	5
MEK1_VHL	12	22	PARP2_CRBN	1	3
MEK2_CRBN	4	19	PBRM1_VHL	1	2
MEK2_VHL	12	15	PCAF_CRBN	1	1
MerTK_CRBN	1	0	PD-L1_CRBN	0	28
NS3_CRBN	1	2	PDEdelta_CRBN	1	11
PDEdelta_VHL	1	0	SHP2_CRBN	6	10
PDL1_CRBN	0	19	SHP2_VHL	15	8
PI3Kalpha_CRBN	1	3	SIRT2_CRBN	0	3
PLK1_CRBN	2	0	SMARCA2_VHL	6	1
PRMT5_VHL	0	6	SMARCA4_VHL	6	1
RAR_cIAP1	1	0	STAT3_CRBN	4	19
RIPK2_CRBN	2	0	Src_CRBN	0	11
RIPK2_VHL	2	0	TBK1_CRBN	0	2
RIPK2_cIAP1	2	1	TRIM24_VHL	0	2
RPS6KA1_CRBN	1	1	TRKA_CRBN	19	0
TRKB_CRBN	9	1	c-Met_VHL	1	0
TRKC_CRBN	8	3	eIF4E_CRBN	6	15
TRKC_MDM2	0	2	eIF4E_VHL	2	1
VEGFR-2_VHL	0	5	p38alpha_CRBN	7	16
VHL_VHL	2	5	p38alpha_VHL	3	5
WDR5_CRBN	0	5	p38beta_CRBN	2	0
WDR5_VHL	0	15	p38delta_CRBN	1	0
Wee1_CRBN	4	0	p38delta_VHL	3	5
c-KIT_CRBN	0	15			
c-Met_CRBN	0	12			

Supplementary Table 3. Densitometry quantifications of Western blotting data in order (normalized by GAPDH using the Image J software and DMSO was used as negative control) for easy comparison between two cell lines.

Compound ID	concentration (nM, in MCF-7 cell)				concentration (nM, in T-47D cell)			
	1	10	50	100	1	10	50	100
PROTAC 1	2.58	1.89	0.90	0.81	1.02	0.76	0.28	0.30
PROTAC 2	2.17	2.72	1.51	1.59	1.26	0.99	0.70	0.58
PROTAC 3	2.08	1.89	1.36	1.19	1.06	0.83	0.43	0.32
PROTAC 4	0.83	0.62	0.31	0.26	1.43	0.38	0.12	0.08
PROTAC 5	1.19	0.70	0.29	0.21	1.00	0.31	0.04	0.02
PROTAC 6	1.15	0.52	0.26	0.16	1.42	0.28	0.05	0.06
PROTAC 7	1.15	0.63	0.20	0.11	1.12	0.43	0.05	0.04
PROTAC 8	1.08	0.36	0.12	0.08	0.68	0.25	0.06	0.04
PROTAC 9	1.10	0.84	0.38	0.22	1.28	0.67	0.31	0.22
PROTAC 10	1.12	1.21	1.02	0.60	1.25	1.08	1.05	0.71
PROTAC 11	1.24	1.15	0.82	0.94	1.34	1.08	0.78	0.64
PROTAC 12	0.88	0.81	0.22	0.27	1.41	0.79	0.25	0.07
PROTAC 13	1.12	1.06	1.30	0.24	1.07	0.77	0.14	0.05
PROTAC 14	1.06	1.03	0.59	0.33	1.71	1.77	0.46	0.18
PROTAC 15	0.92	0.92	0.47	0.30	1.33	0.75	0.07	0.04
PROTAC 16	0.88	0.93	0.67	0.97	1.16	1.15	1.15	1.22

Supplementary Table 4. The prediction results of DeepPROTACs model on our experimental dataset.

PROTAC ID	Expt.	Prediction
**PROTAC 1**	**1**	**1**
PROTAC 2	0	1
PROTAC 3	0	1
**PROTAC 4**	**1**	**1**
**PROTAC 5**	**1**	**1**
**PROTAC 6**	**1**	**1**
**PROTAC 7**	**1**	**1**
**PROTAC 8**	**1**	**1**
**PROTAC 9**	**1**	**1**
PROTAC 10	0	1
PROTAC 11	0	1
**PROTAC 12**	**1**	**1**
**PROTAC 13**	**1**	**1**
**PROTAC 14**	**1**	**1**
PROTAC 15	1	0
**PROTAC 16**	**0**	**0**

Note: bold means correct prediction.

Supplementary Table 5. The evaluation results of DeepPROTACs model on different targets.

Targets	EZH2	STAT3	eIF4E	FLT-3
Accuracy on Test set	76.67%	77.99%	79.77%	77.22%
Accuracy on Targets	77.78%	75.36%	65.28%	80.00%

Supplementary Table 6. The encoding table for SMILES.

character	frequency	code	character	frequency	code
'C'	16867032	1	'\\'	40666	21
'('	7201666	2	'3'	1203951	22
'='	3164974	3	'4'	149622	23
'O'	4806890	4	'l'	208445	24
')'	7201666	5	'F'	463503	25
'N'	3274399	6	'o'	263404	26
'['	2272539	7	'I'	9154	27
'@'	2787747	8	'B'	67994	28
'H'	1906099	9	'r'	62314	29
']'	2272539	10	'P'	2852	30
'1'	5943024	11	'5'	7974	31
'c'	15747890	12	'6'	1724	32
'n'	2818752	13	'i'	583	33
'/'	183604	14	'7'	616	34
'2'	3746324	15	'8'	310	35
'#'	85212	16	'9'	86	36
'S'	437668	17	'%'	42	37
's'	242192	18	'0'	24	38
'+'	100208	19	'p'	2	39
'-'	340633	20			

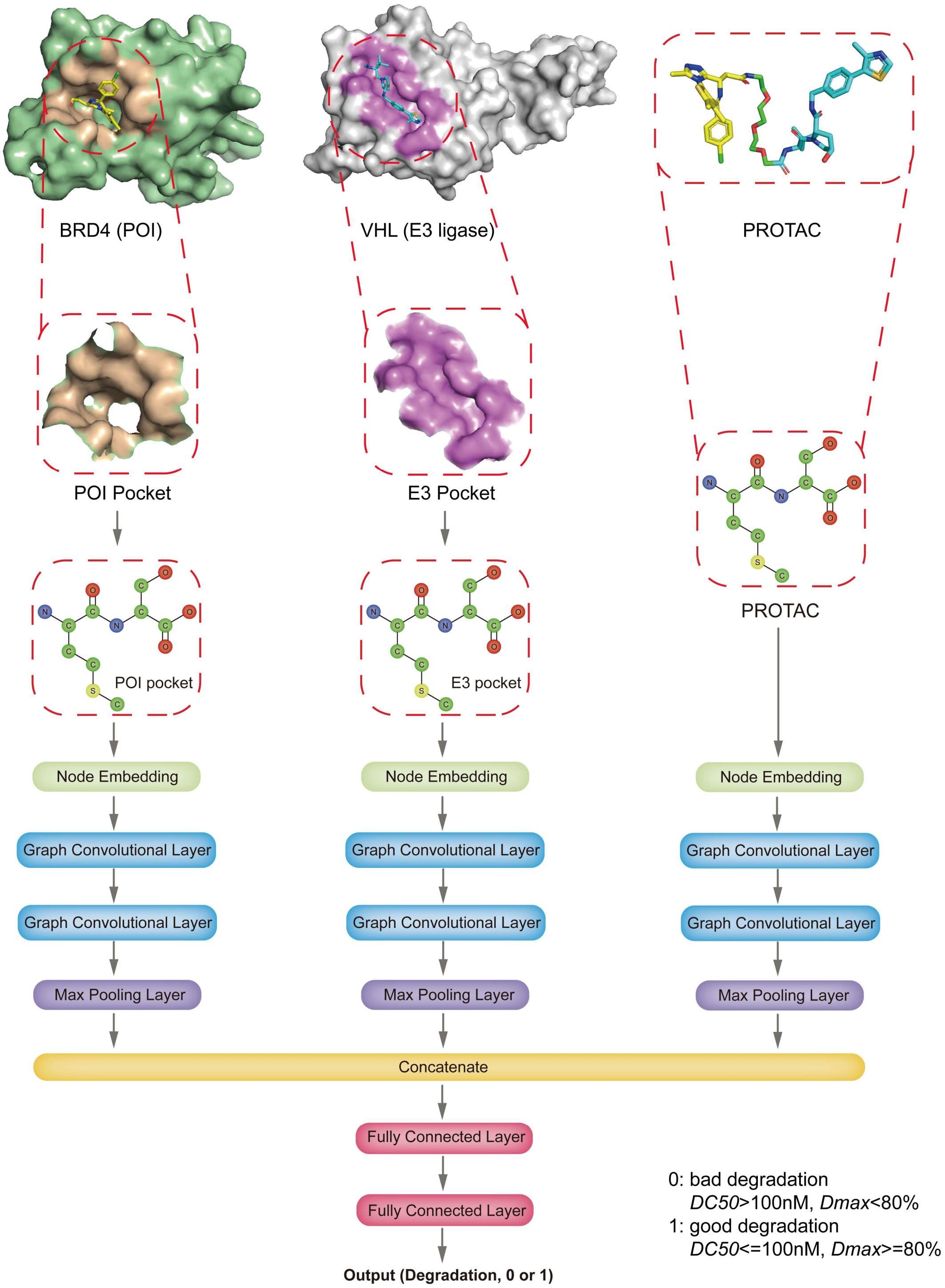

Supplementary Fig.1. The network architecture of an alternative model. The preprocess of POI, E3 ligase and PROTAC molecule: extraction of binding pocket from POI (BRD4) and E3 ligase (VHL) and conversion to graph representations, conversion of PROTAC molecule to graph representation.

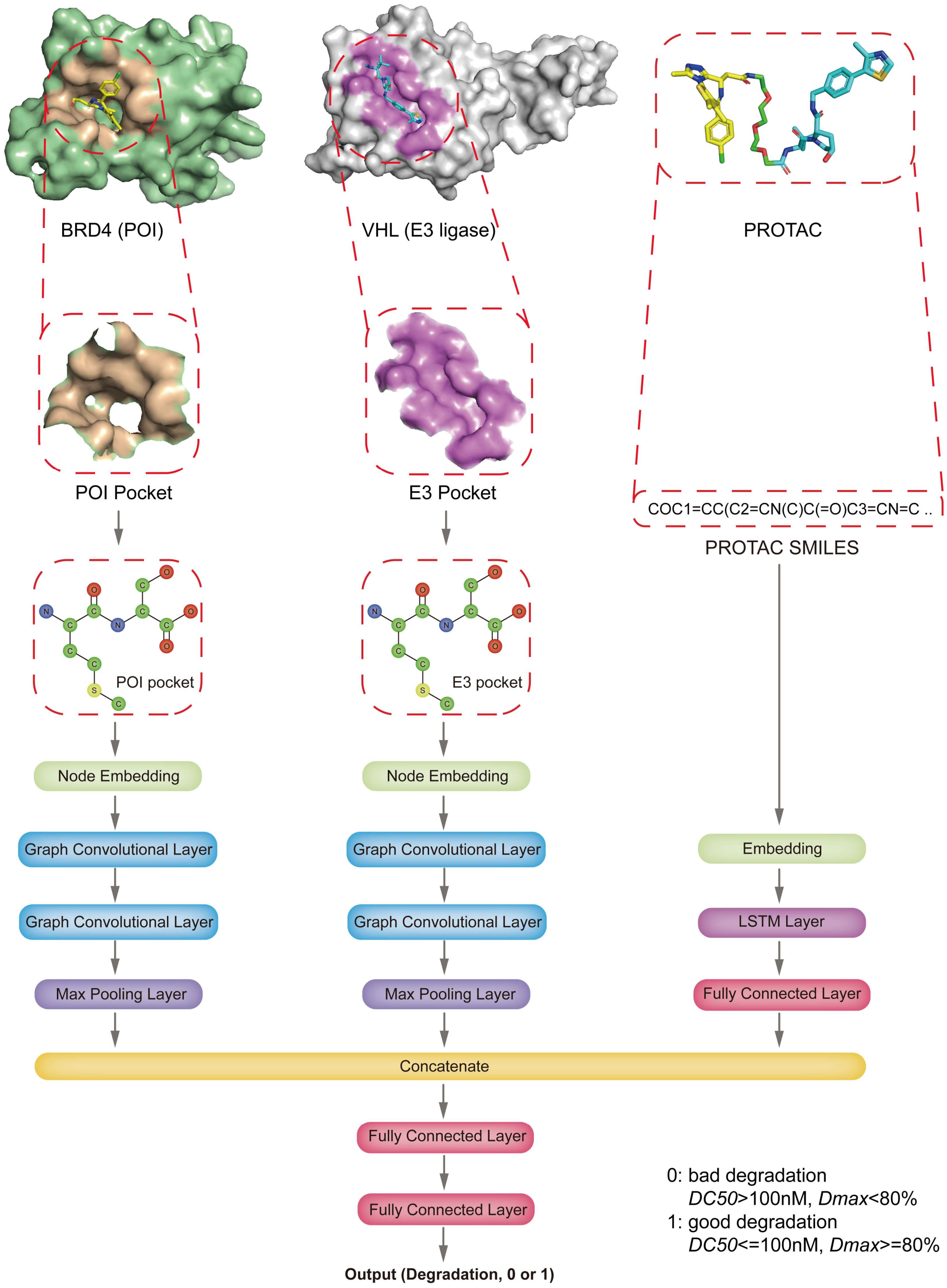

Supplementary Fig.2. The network architecture of an alternative model. The preprocess of POI, E3 ligase and PROTAC molecule: extraction of binding pocket from POI (BRD4) and E3 ligase (VHL) and conversion to graph representations, conversion of PROTAC molecule to SMILES.

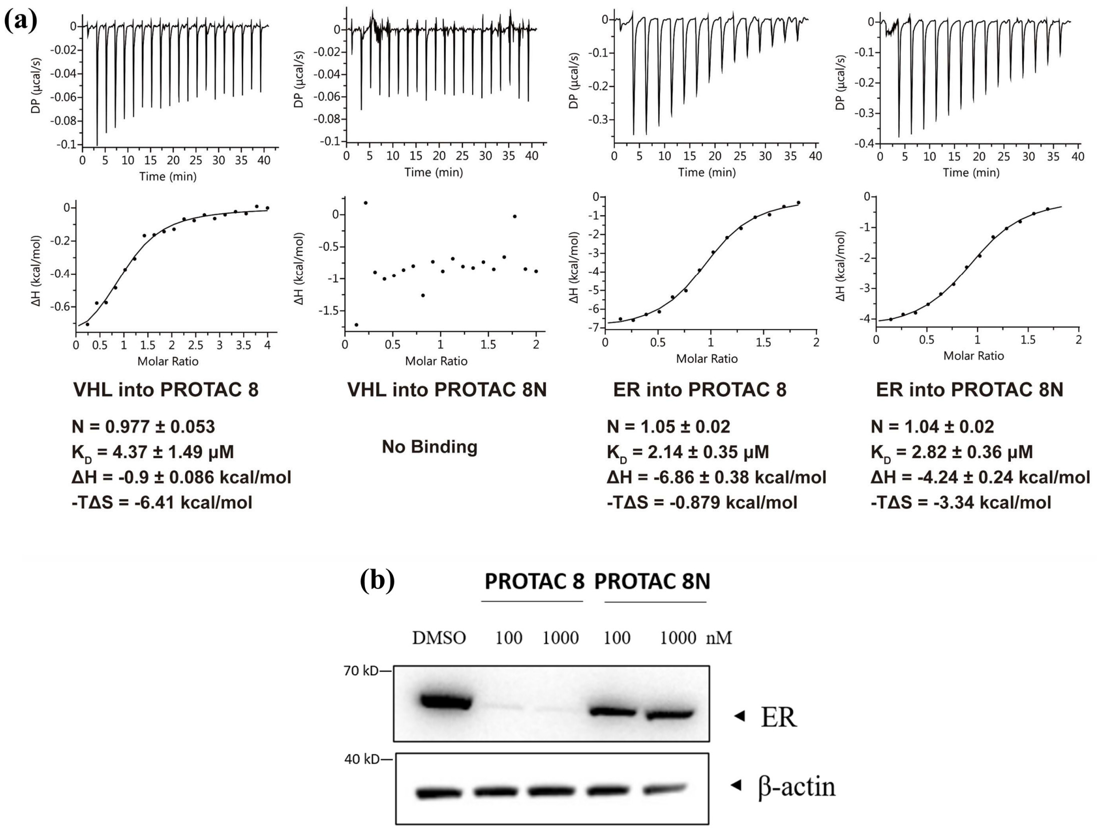

Supplementary Fig.3. Validation of ER PROTACs. (a) Thermodynamic data, representative titration raw data, and fitting curves of VHL and ER titrated into PROTAC 8 and PROTAC 8N. Binding kinetic and thermodynamic data are shown below as mean values ± SD. Source Data are provided as a Source Data file. (b) Western blotting analysis of ER in T-47D cell line by PROTAC 8 and negative compound PROTAC 8N. All western blot data are representative of at least two independent replicates.

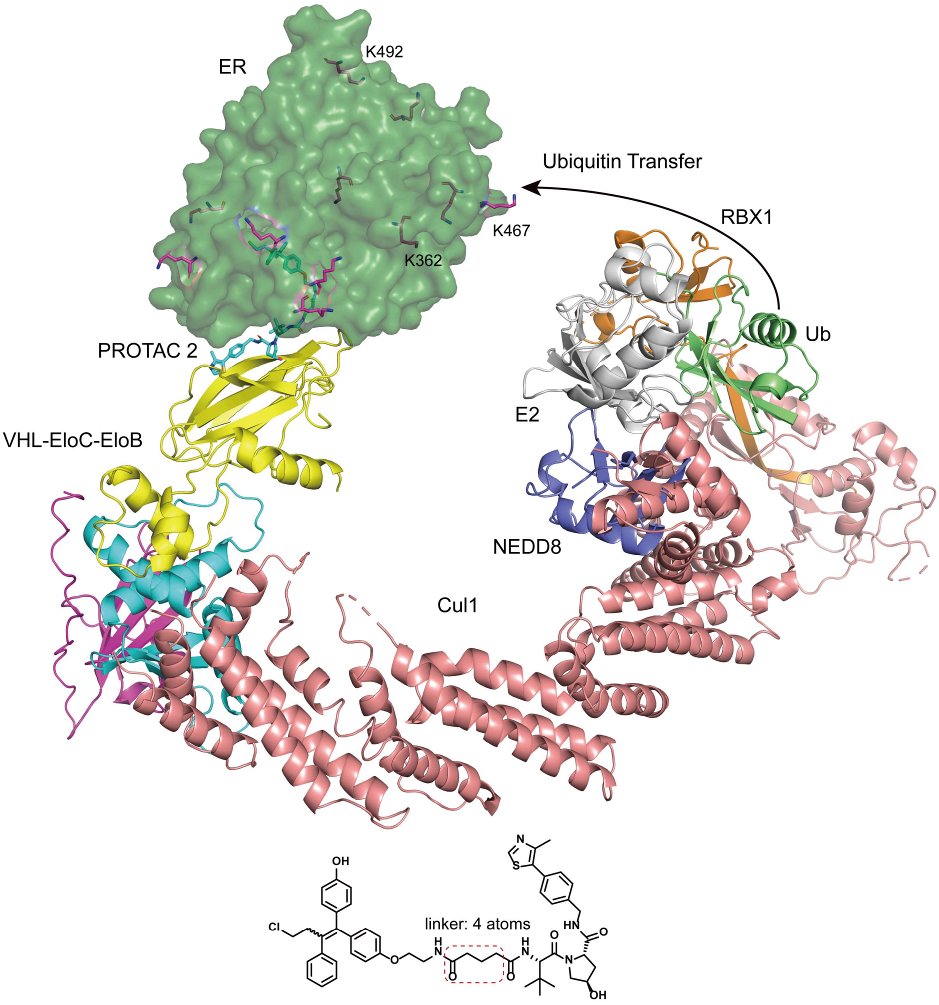

Supplementary Fig.4. Computational model of whole CRLs (Cullin-Ring E3 ubiquitin Ligases) induced by PROTAC 2. The ternary complex (ER, VHL-EloC-EloB and PROTAC 2) is aligned with crystal structure of NEDD8-CUL1-RBX1-UB-E2 (PDB ID: 6TTU) with the help of Cul2-Rbx1-EloBC-VHL structure (PDB ID: 5N4W). The structure of 5N4W was hidden for the sake of clarity.

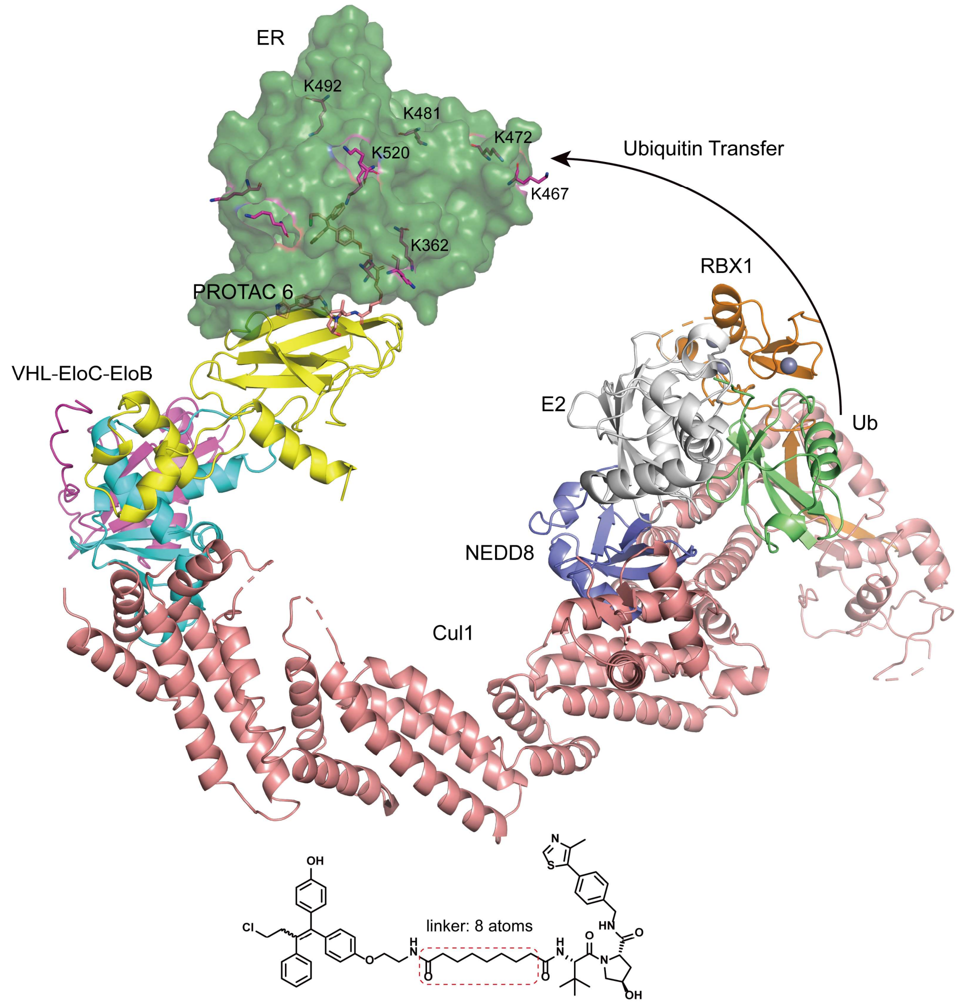

PROTAC 6

Supplementary Fig.5. Computational model of whole CRLs (Cullin-Ring E3 ubiquitin Ligases) induced by PROTAC 6. The ternary complex (ER, VHL-EloC-EloB and PROTAC 6) is aligned with crystal structure of NEDD8-CUL1-RBX1-UB-E2 (PDB ID: 6TTU) with the help of Cul2-Rbx1-EloBC-VHL structure (PDB ID: 5N4W). The structure of 5N4W was hidden for the sake of clarity.

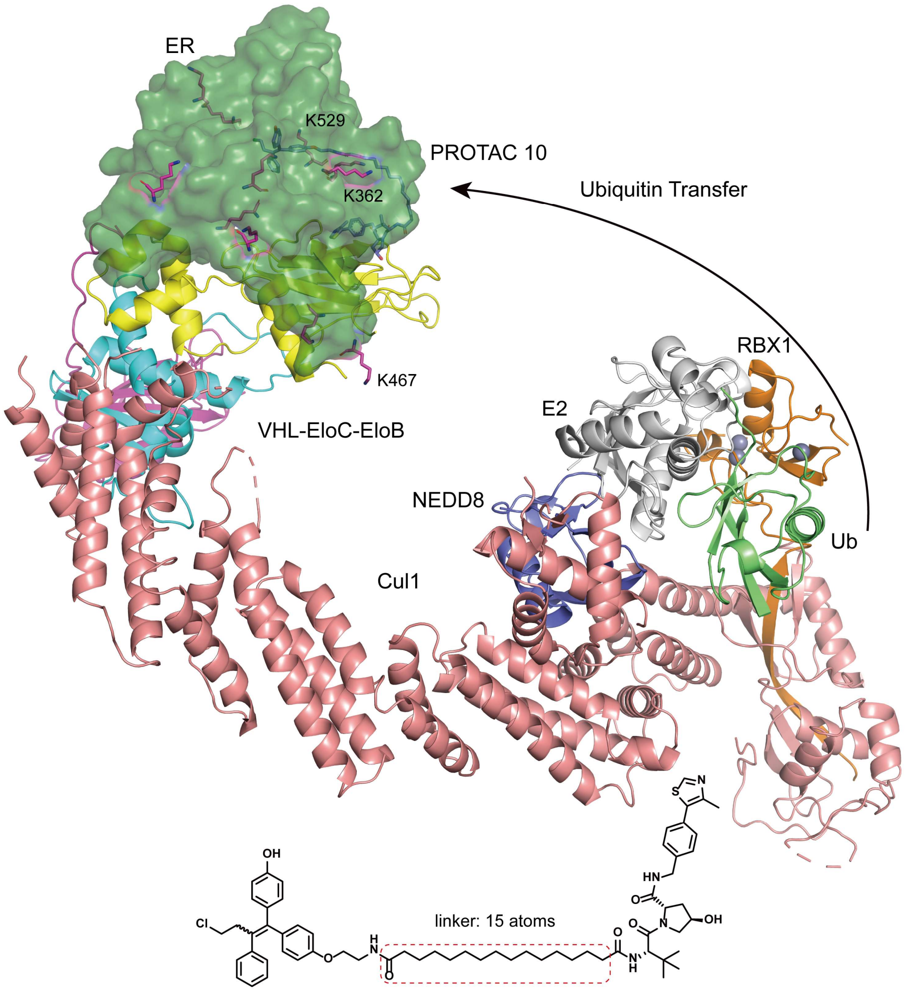

PROTAC 10

Supplementary Fig.6. Computational model of whole CRLs (Cullin-Ring E3 ubiquitin Ligases) induced by PROTAC 10. The ternary complex (ER, VHL-EloC-EloB and PROTAC 10) is aligned with crystal structure of NEDD8-CUL1-RBX1-UB-E2 (PDB ID: 6TTU) with the help of Cul2-Rbx1-EloBC-VHL structure (PDB ID: 5N4W). The structure of 5N4W was hidden for the sake of clarity.

Supplementary Fig.7. Unprocessed blots related to Figure 6. Protein marker (Thermo Scientific™, #26616) was used. Membranes were cropped according to the molecule weight of ER and GAPDH before exposure.

MCF-7 cell line

ER PROTAC 1, PROTAC 2, PROTAC 3

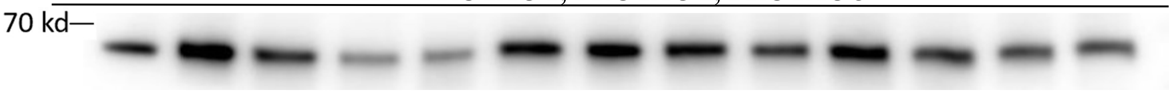

ER PROTAC 4, Fulvstrant, Toremifene

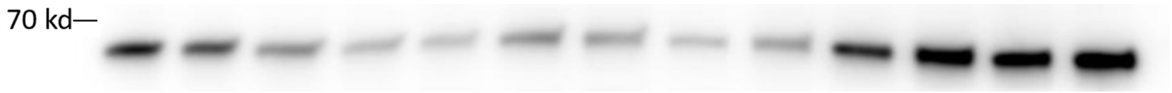

ER PROTAC 5, PROTAC 6, PROTAC 7

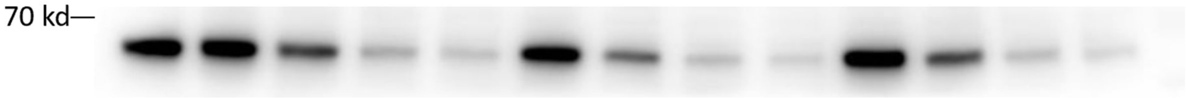

ER PROTAC 8, PROTAC 9, PROTAC 10

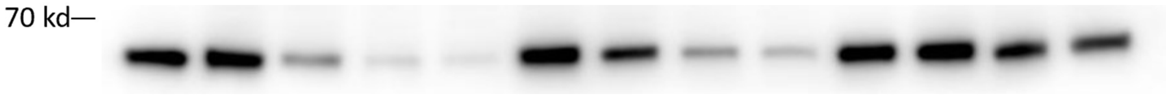

ER PROTAC 11, PROTAC 12, PROTAC 13

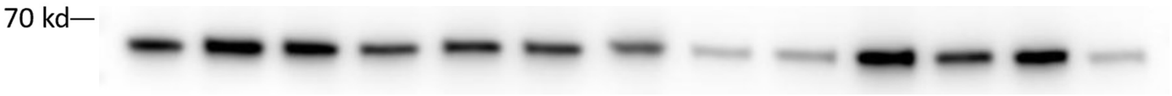

ER PROTAC 14, PROTAC 15, PROTAC 16

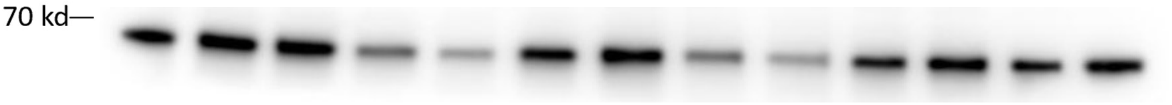

MCF-7 cell line

GAPDH PROTAC 1, PROTAC 2, PROTAC 3

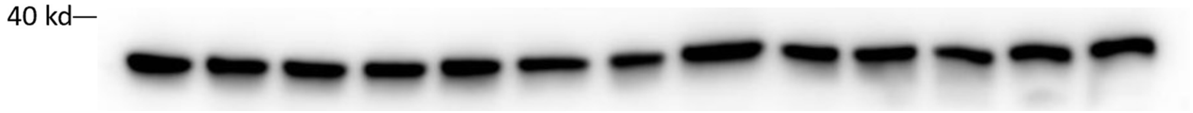

GAPDH PROTAC 4, Fulvstrant, Toremifene

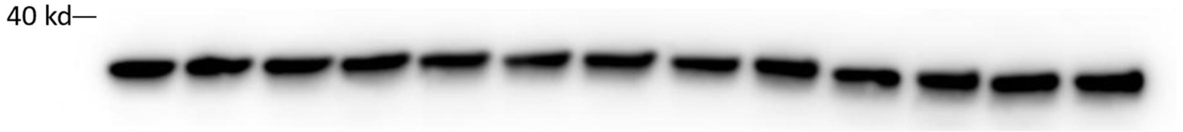

GAPDH PROTAC 5, PROTAC 6, PROTAC 7

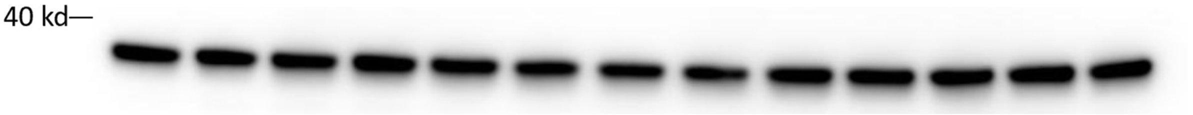

GAPDH PROTAC 8, PROTAC 9, PROTAC 10

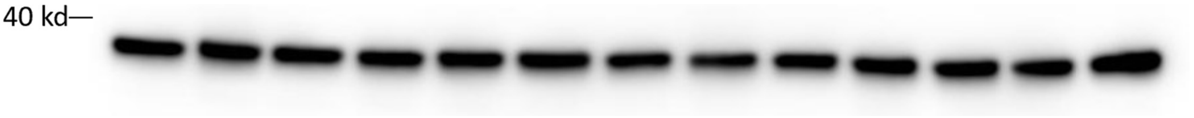

GAPDH PROTAC 11, PROTAC 12, PROTAC 13

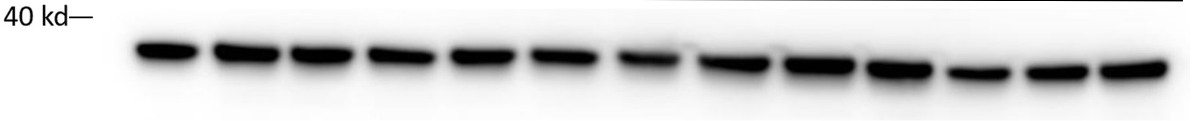

GAPDH PROTAC 14, PROTAC 15, PROTAC 16

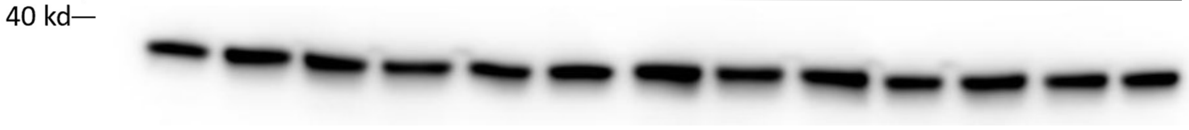

T-47D cell line

ER PROTAC 16, PROTAC 1, PROTAC 2

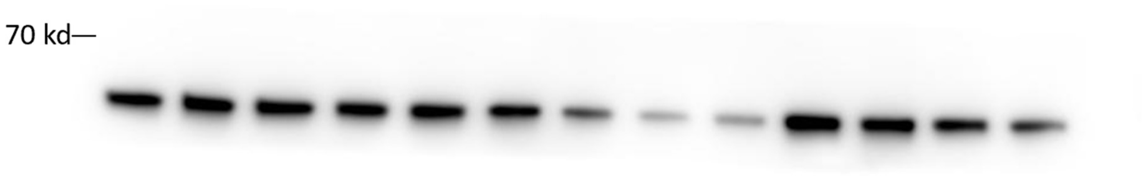

ER PROTAC 5, PROTAC 4, PROTAC 3

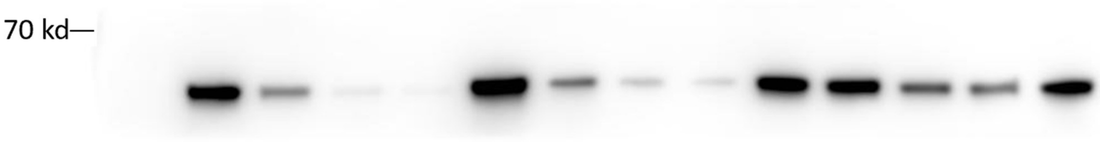

ER PROTAC 8, PROTAC 7, PROTAC 6

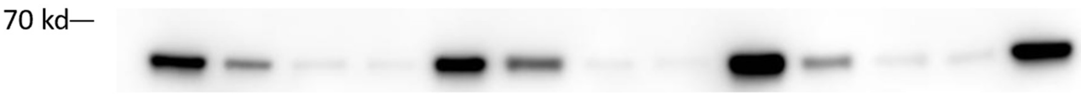

ER PROTAC 9, PROTAC 10, PROTAC 11

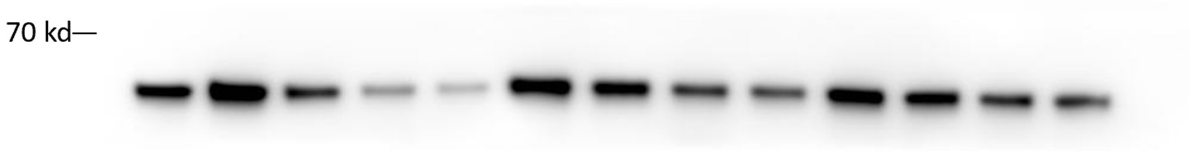

ER PROTAC 13, PROTAC 14, PROTAC 15

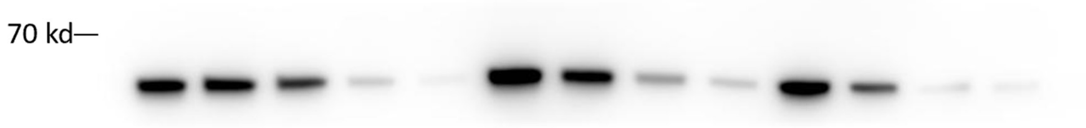

ER   PROTAC 12, Fulvstrant

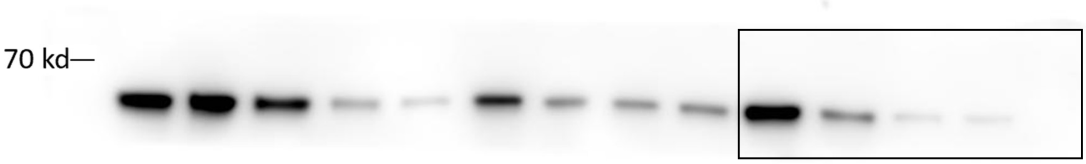

Not related in this article

T-47D cell line

GAPDH   PROTAC 16, PROTAC 1, PROTAC 2

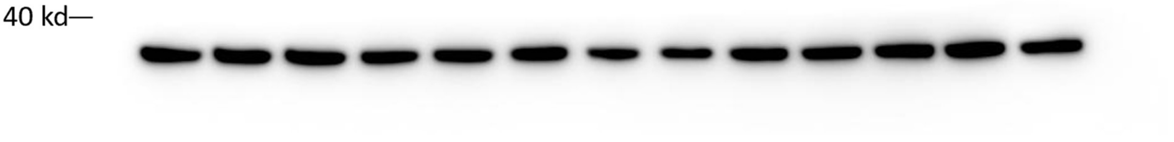

GAPDH   PROTAC 5, PROTAC 4, PROTAC 3

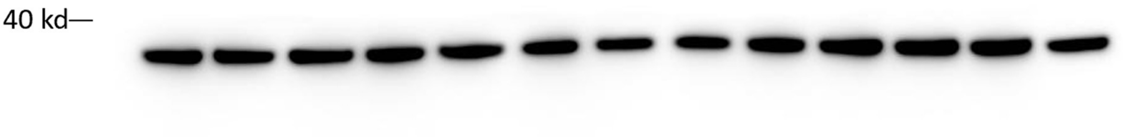

GAPDH   PROTAC 8, PROTAC 7, PROTAC 6

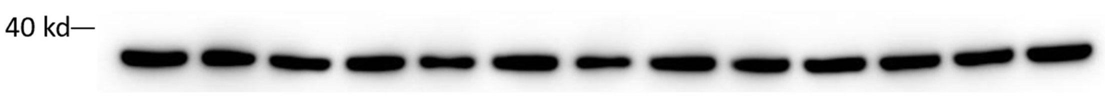

GAPDH PROTAC 9, PROTAC 10, PROTAC 11

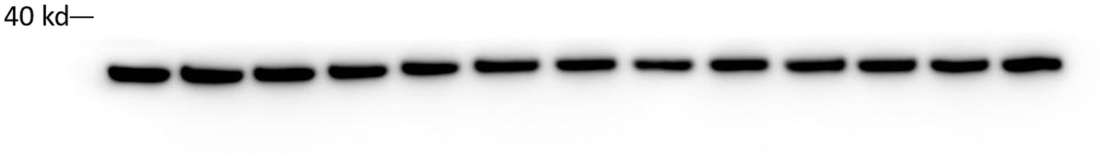

GAPDH PROTAC 13, PROTAC 14, PROTAC 15

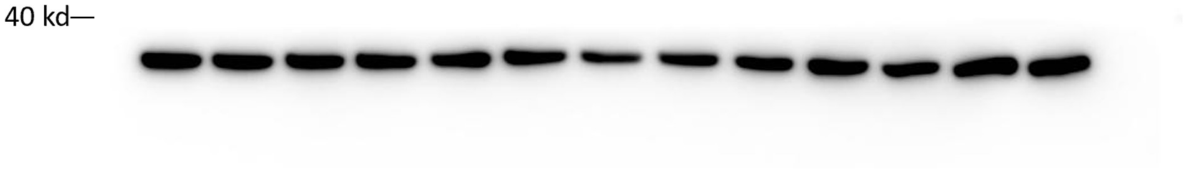

GAPDH PROTAC 12, Fulvstrant

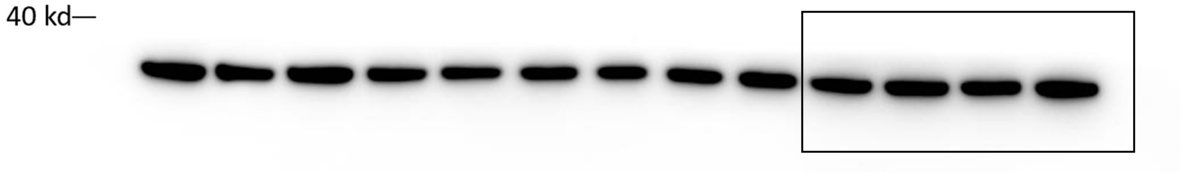

Not related in this article

Supplementary Fig.8. Unprocessed blots related to Supplementary Fig. 3b. Protein marker (Thermo Scientific™, #26616) was used. Membranes were cropped according to the molecule weight of ER and β-actin before exposure.

T-47D cell line

ER

70 kd—

β-actin

40 kd—

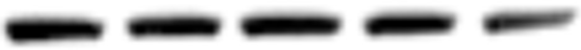

Supplementary Fig.9. HRMS spectrometry data of ER PROTACs.

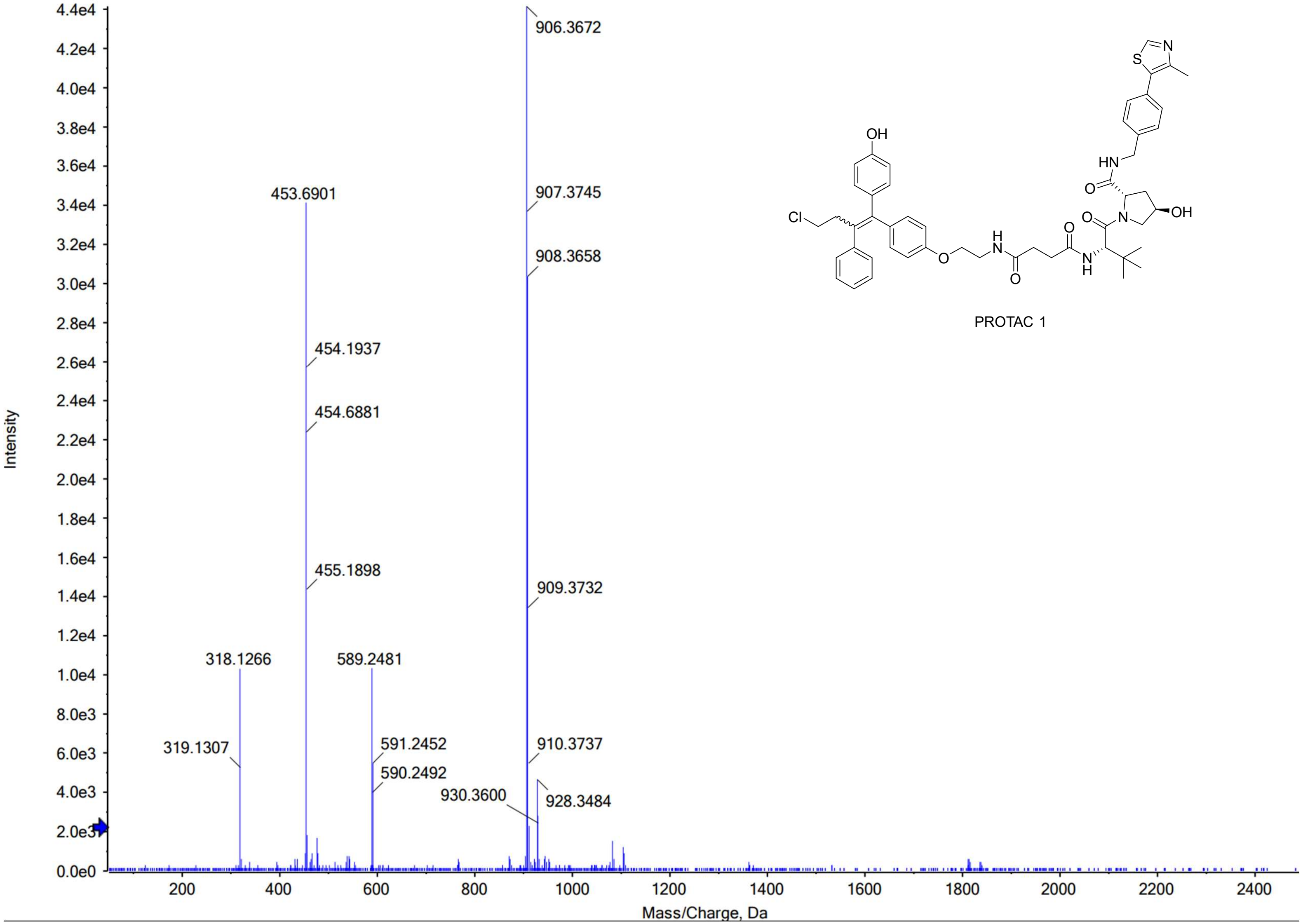

906.3672
907.3745
908.3658
909.3732
910.3737
928.3484
930.3600
453.6901
454.1937
454.6881
455.1898
318.1266
319.1307
589.2481
591.2452
590.2492
Intensity
Mass/Charge, Da

PROTAC 1

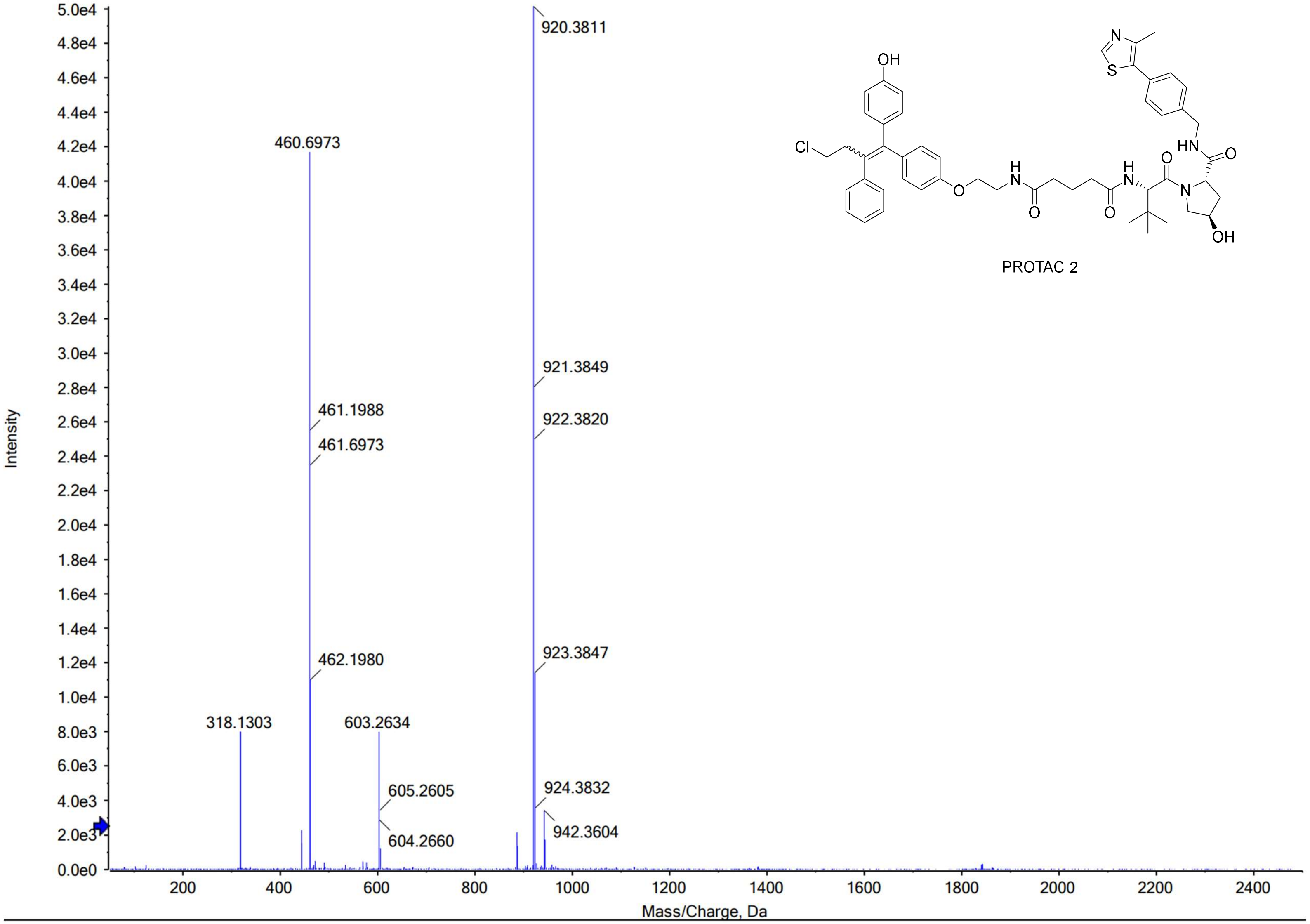
920.3811
921.3849
922.3820
923.3847
924.3832
942.3604
460.6973
461.1988
461.6973
462.1980
318.1303
603.2634
605.2605
604.2660
PROTAC 2
Intensity
Mass/Charge, Da

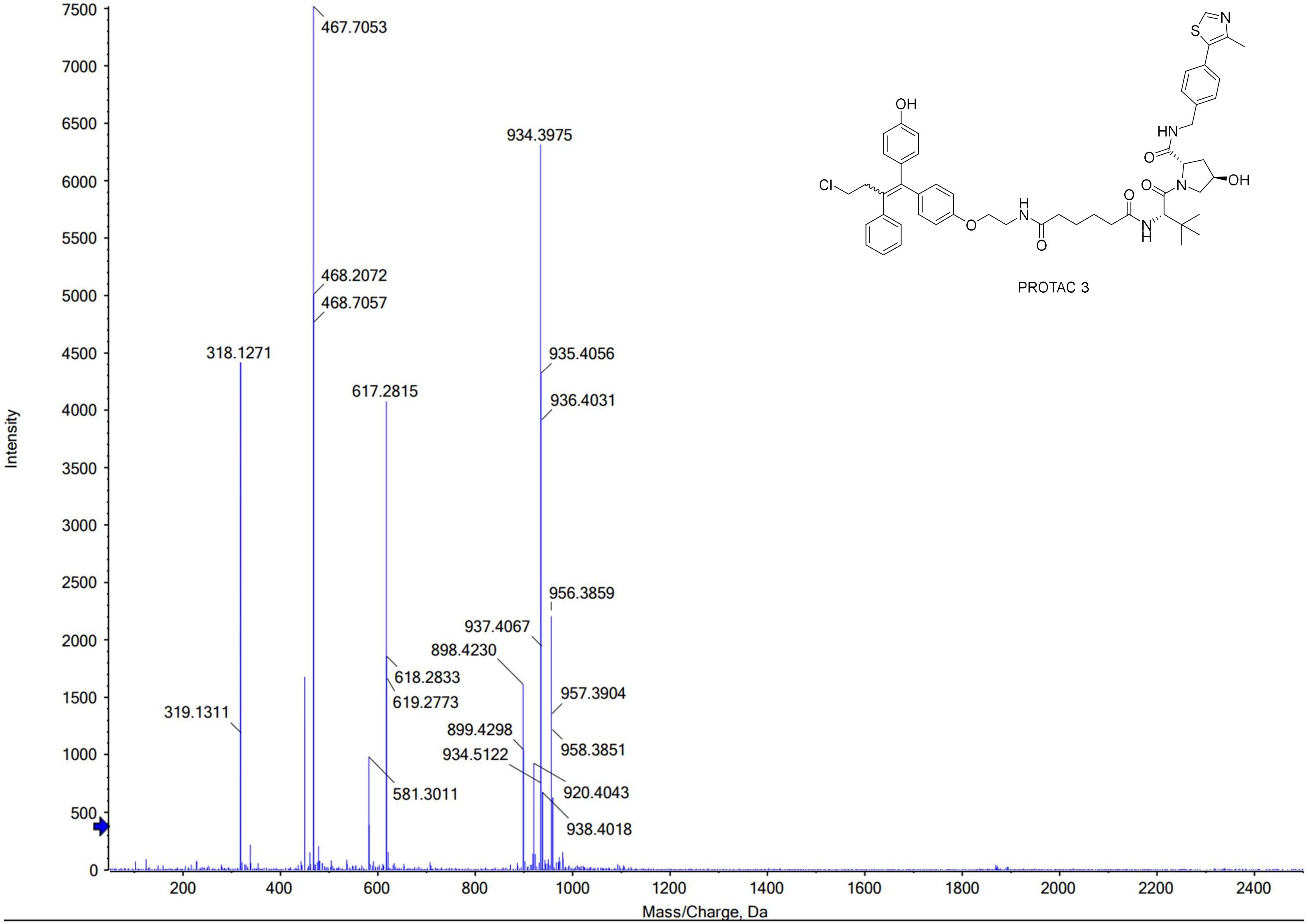
7500
7000
6500
6000
5500
5000
4500
4000
3500
3000
2500
2000
1500
1000
500
0
Intensity
200
400
600
800
1000
1200
1400
1600
1800
2000
2200
2400
Mass/Charge, Da
467.7053
934.3975
468.2072
468.7057
318.1271
935.4056
617.2815
936.4031
956.3859
937.4067
898.4230
618.2833
619.2773
957.3904
319.1311
899.4298
958.3851
934.5122
920.4043
581.3011
938.4018
OH
Cl
O
H
N
O
O
H
N
O
N
OH
O
HN
S
N

PROTAC 3

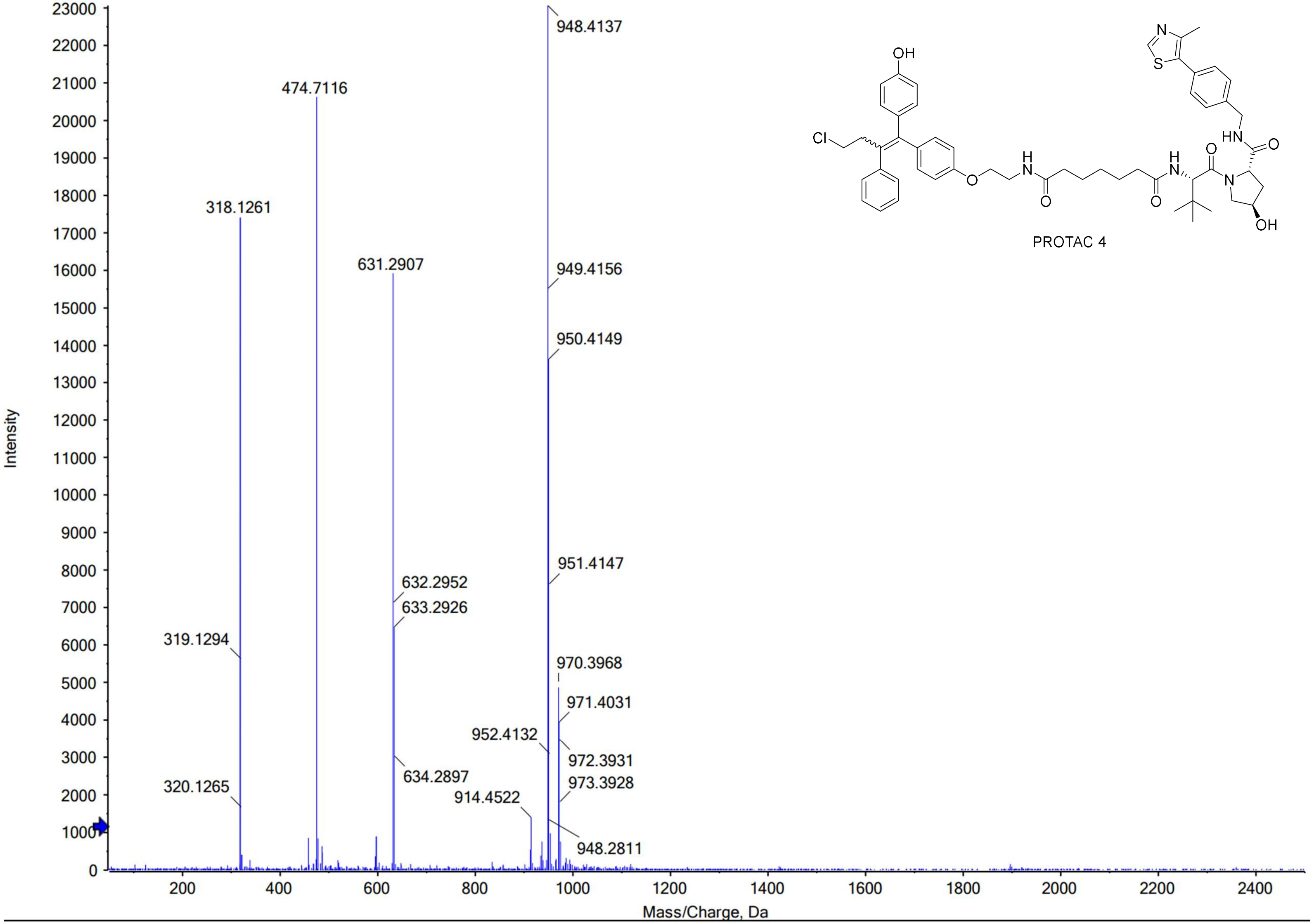

Intensity
Mass/Charge, Da
948.4137
474.7116
318.1261
631.2907
949.4156
950.4149
951.4147
632.2952
633.2926
319.1294
970.3968
971.4031
952.4132
972.3931
973.3928
634.2897
320.1265
914.4522
948.2811

PROTAC 4

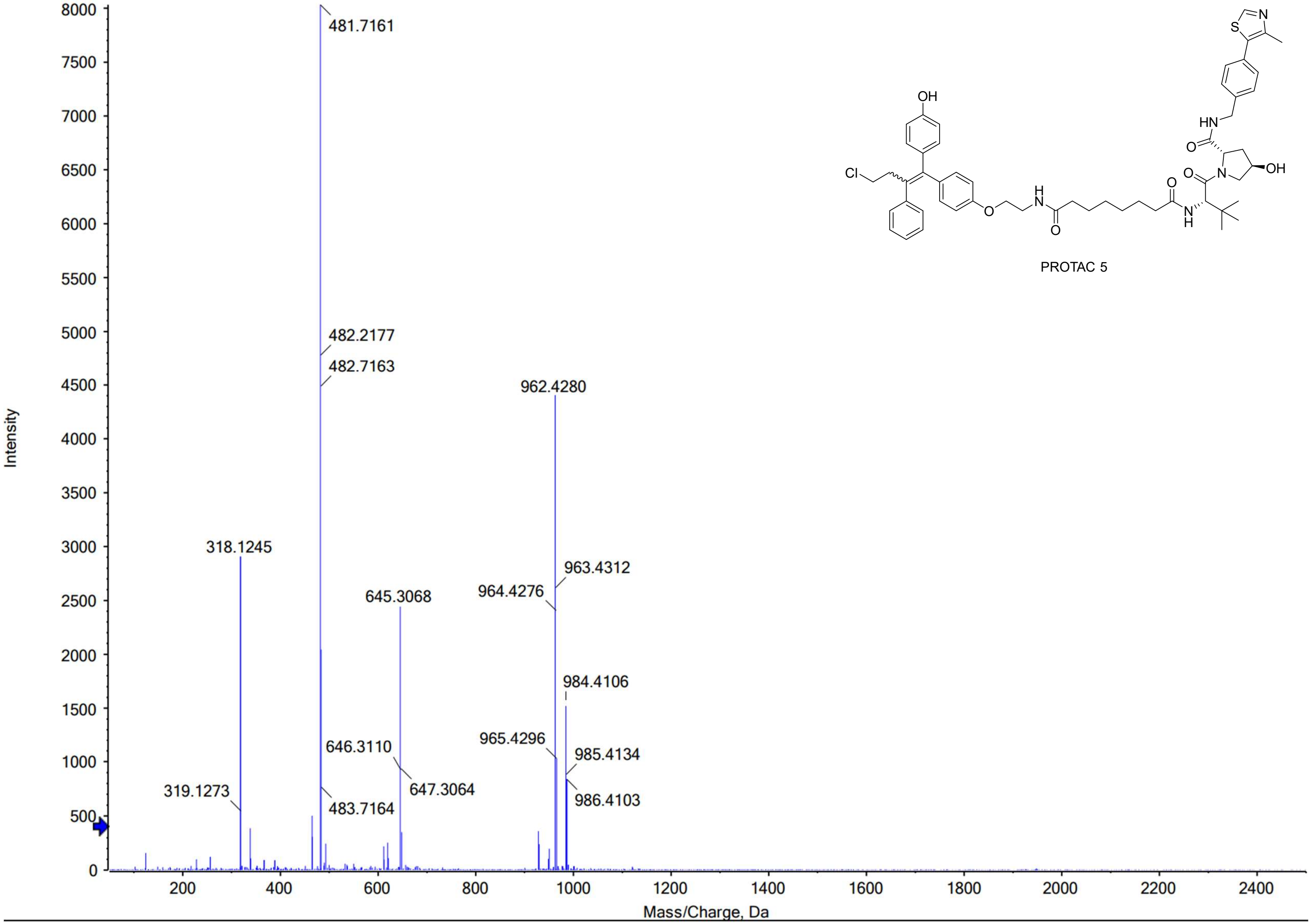

481.7161
482.2177
482.7163
962.4280
318.1245
963.4312
964.4276
645.3068
984.4106
646.3110
965.4296
985.4134
319.1273
647.3064
483.7164
986.4103
Intensity
Mass/Charge, Da
OH
Cl
HN
O
OH
N
S

PROTAC 5

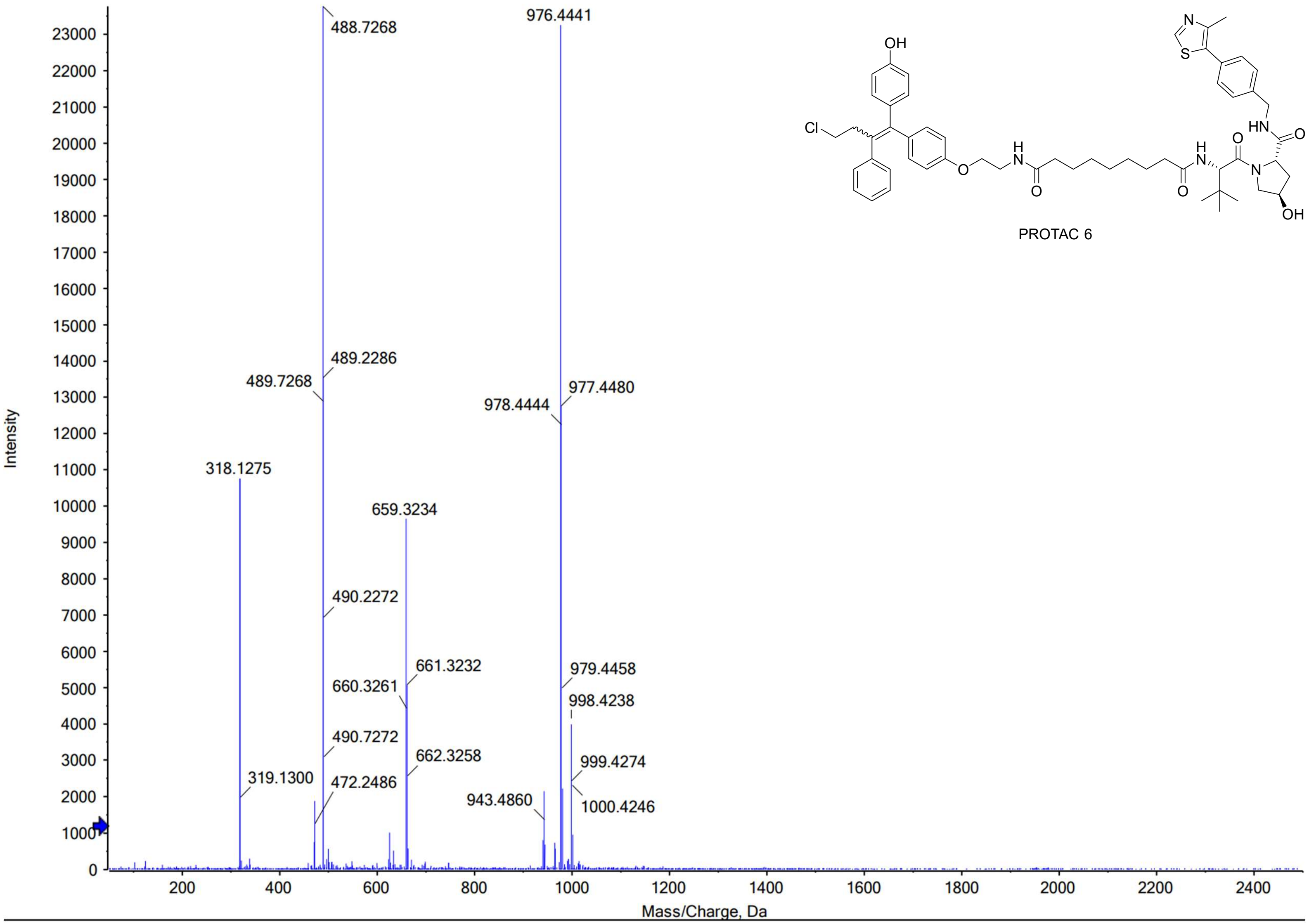

488.7268
976.4441
489.2286
489.7268
977.4480
978.4444
318.1275
659.3234
490.2272
661.3232
979.4458
660.3261
998.4238
490.7272
662.3258
999.4274
319.1300
472.2486
943.4860
1000.4246
Intensity
Mass/Charge, Da
OH
Cl
N
S
HN
O
H
N
O
O
OH

PROTAC 6

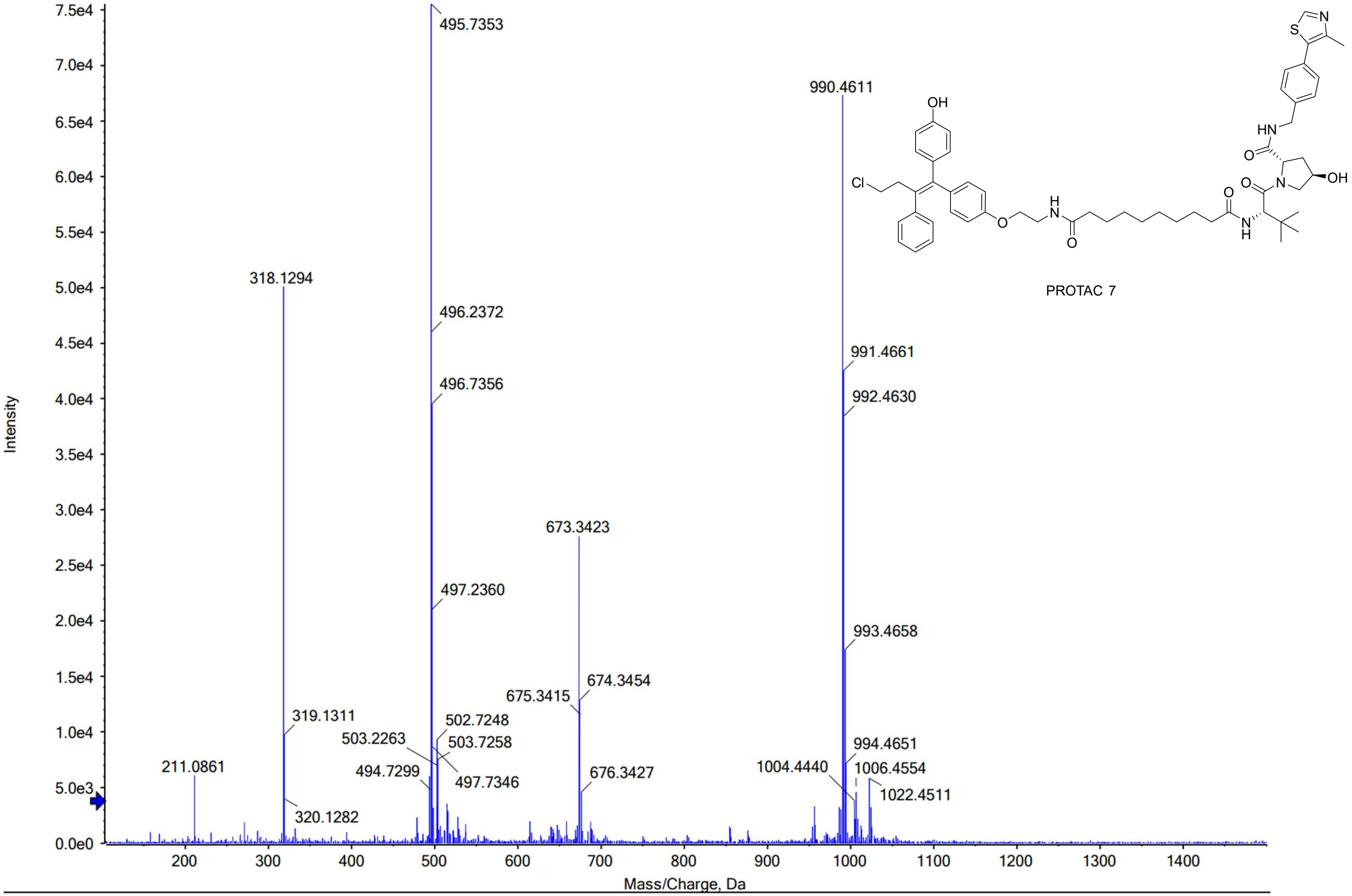

7.5e4
7.0e4
6.5e4
6.0e4
5.5e4
5.0e4
4.5e4
4.0e4
3.5e4
3.0e4
2.5e4
2.0e4
1.5e4
1.0e4
5.0e3
0.0e0
Intensity
495.7353
990.4611
318.1294
496.2372
991.4661
496.7356
992.4630
673.3423
497.2360
993.4658
675.3415
674.3454
319.1311
502.7248
503.2263
503.7258
994.4651
211.0861
494.7299
497.7346
676.3427
1004.4440
1006.4554
1022.4511
320.1282
200
300
400
500
600
700
800
900
1000
1100
1200
1300
1400
Mass/Charge, Da
OH
Cl
O
H
N
O
O
N
H
O
N
OH
HN
O
S
N
PROTAC 7

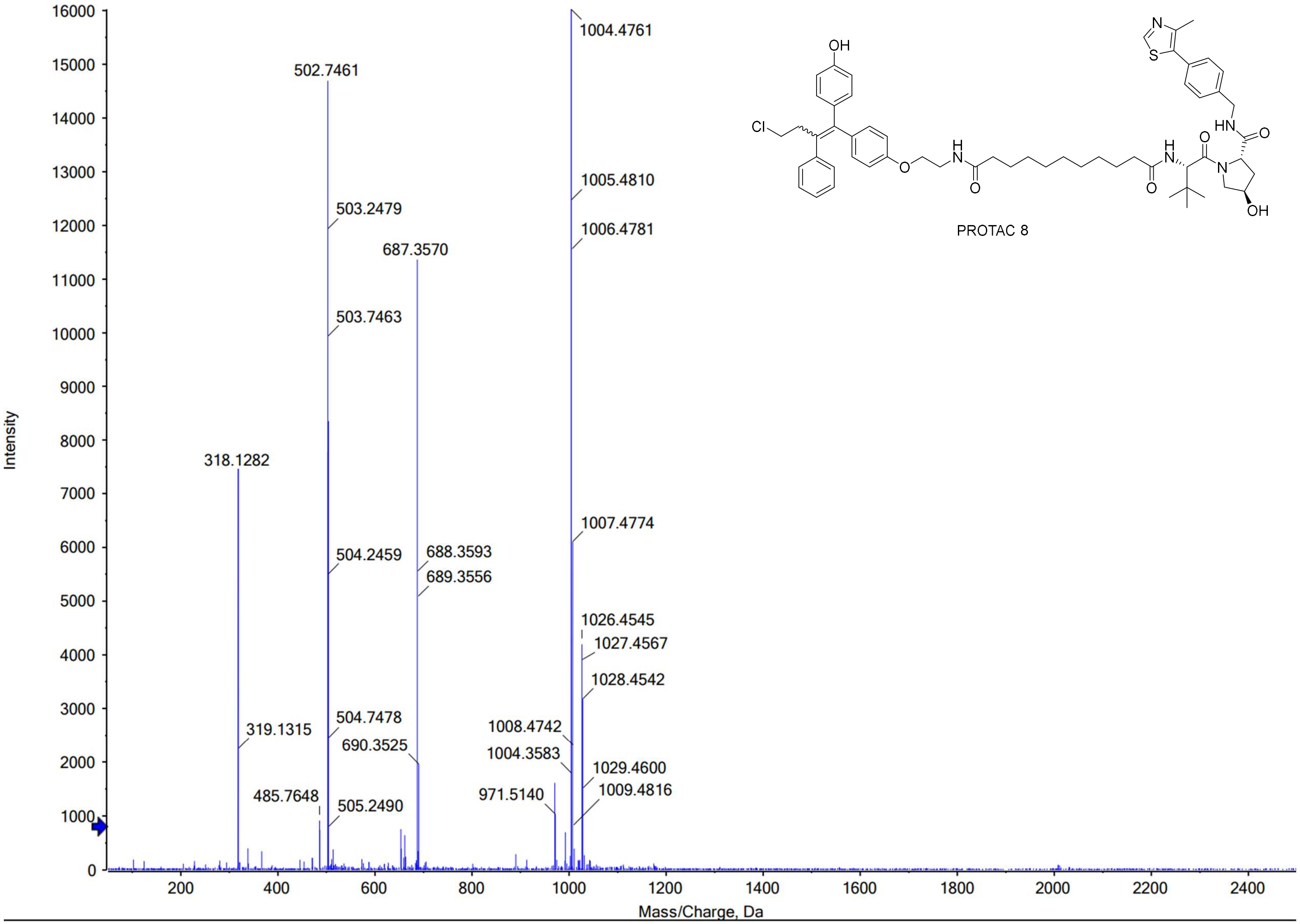

Intensity
Mass/Charge, Da
1004.4761
502.7461
503.2479
1005.4810
1006.4781
687.3570
503.7463
318.1282
1007.4774
504.2459
688.3593
689.3556
1026.4545
1027.4567
1028.4542
319.1315
504.7478
690.3525
1008.4742
1004.3583
1029.4600
1009.4816
485.7648
505.2490
971.5140
OH
Cl
H
N
O
O
H
N
O
O
N
HN
O
OH
N
S

PROTAC 8

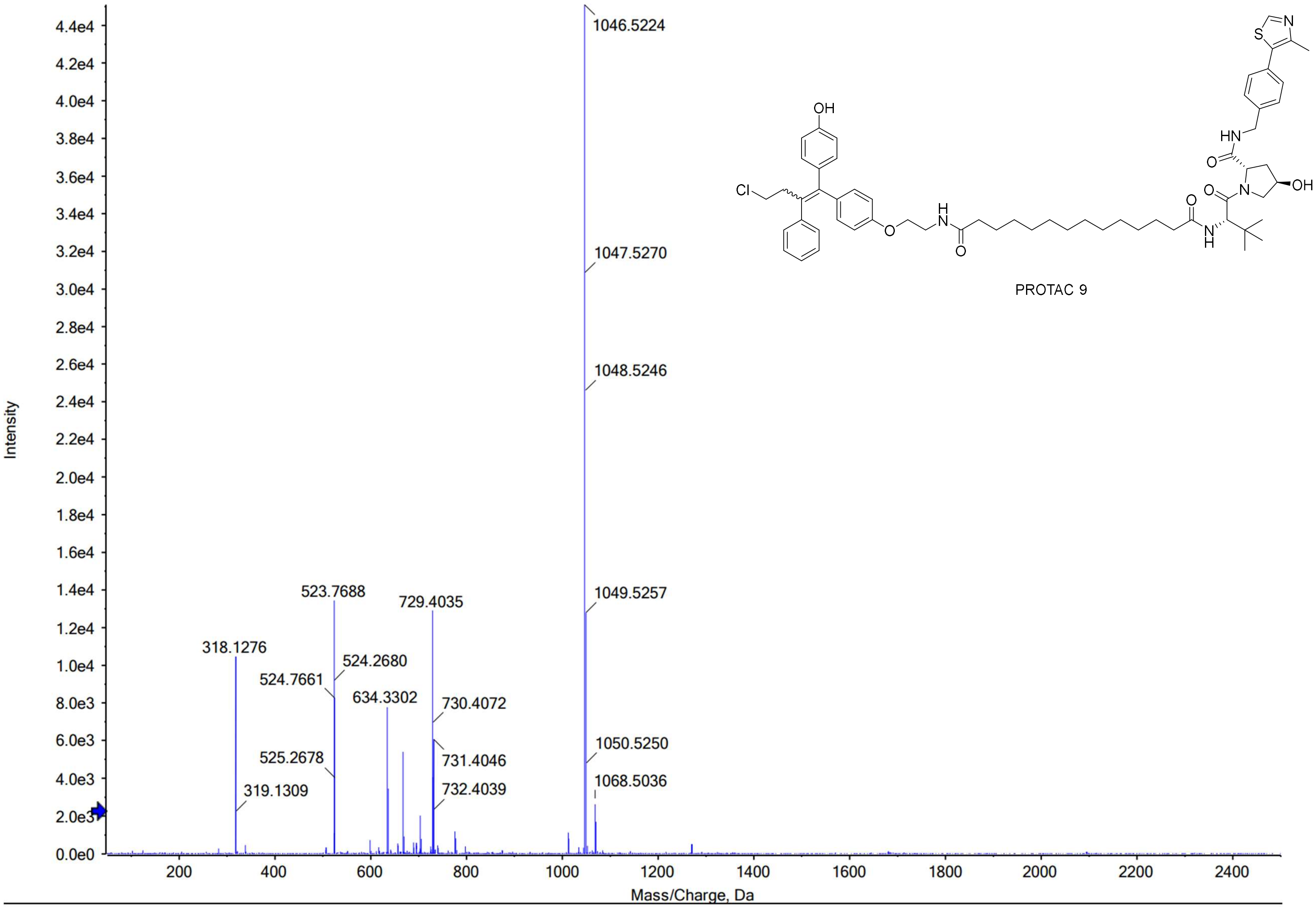

Intensity
Mass/Charge, Da
1046.5224
1047.5270
1048.5246
1049.5257
1050.5250
1068.5036
523.7688
524.2680
524.7661
525.2678
729.4035
730.4072
731.4046
732.4039
634.3302
318.1276
319.1309

PROTAC 9

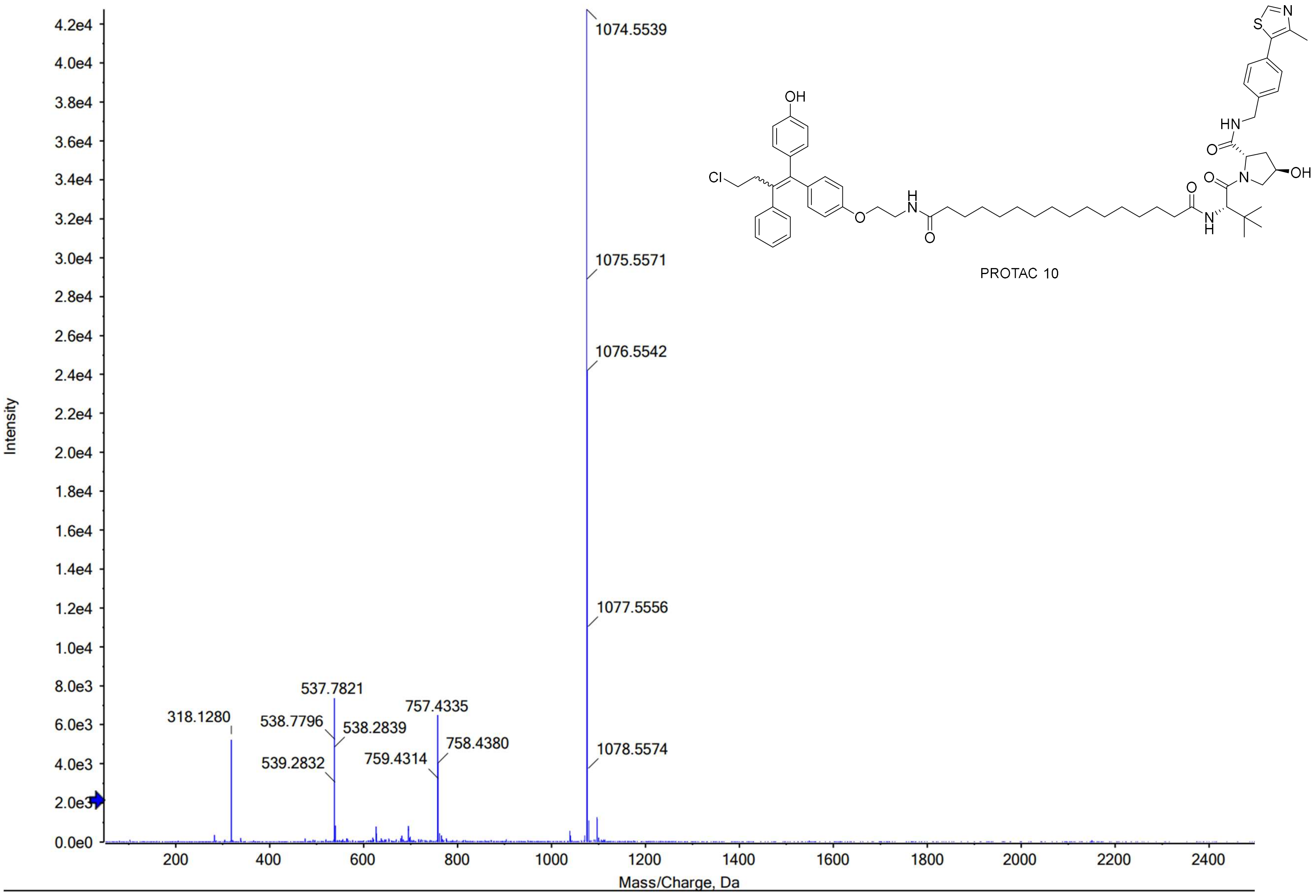

4.2e4
4.0e4
3.8e4
3.6e4
3.4e4
3.2e4
3.0e4
2.8e4
2.6e4
2.4e4
2.2e4
2.0e4
1.8e4
1.6e4
1.4e4
1.2e4
1.0e4
8.0e3
6.0e3
4.0e3
2.0e3
0.0e0
Intensity
1074.5539
1075.5571
1076.5542
1077.5556
1078.5574
318.1280
537.7821
538.7796
538.2839
539.2832
757.4335
758.4380
759.4314
200
400
600
800
1000
1200
1400
1600
1800
2000
2200
2400
Mass/Charge, Da
OH
Cl
O
H
N
O
O
N
H
O
N
OH
O
HN
S
N
PROTAC 10

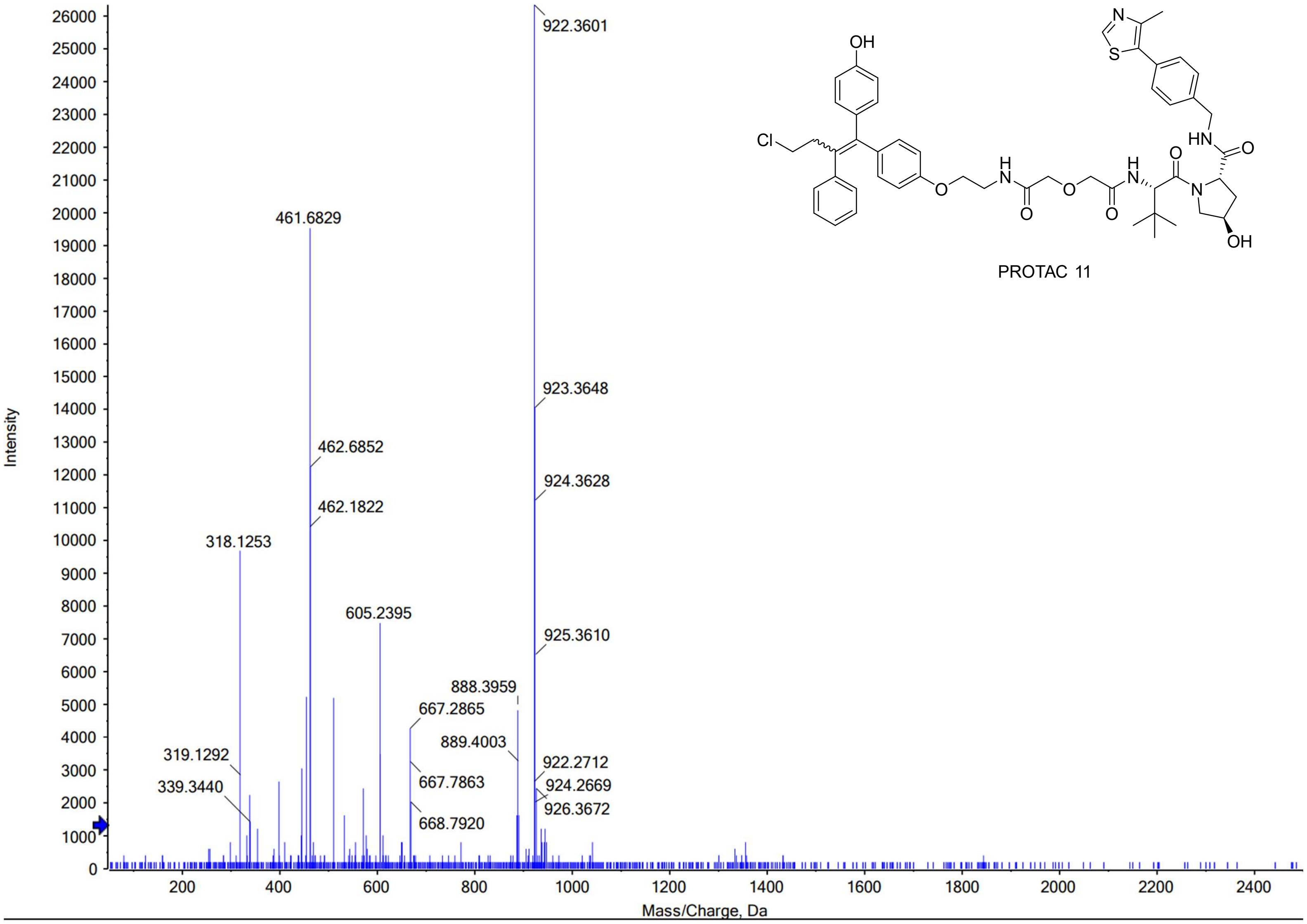
922.3601
461.6829
923.3648
462.6852
924.3628
462.1822
318.1253
605.2395
925.3610
888.3959
667.2865
889.4003
319.1292
922.2712
667.7863
924.2669
339.3440
926.3672
668.7920
Intensity
Mass/Charge, Da
Cl
OH
N
S
H
N
O
HN
PROTAC 11

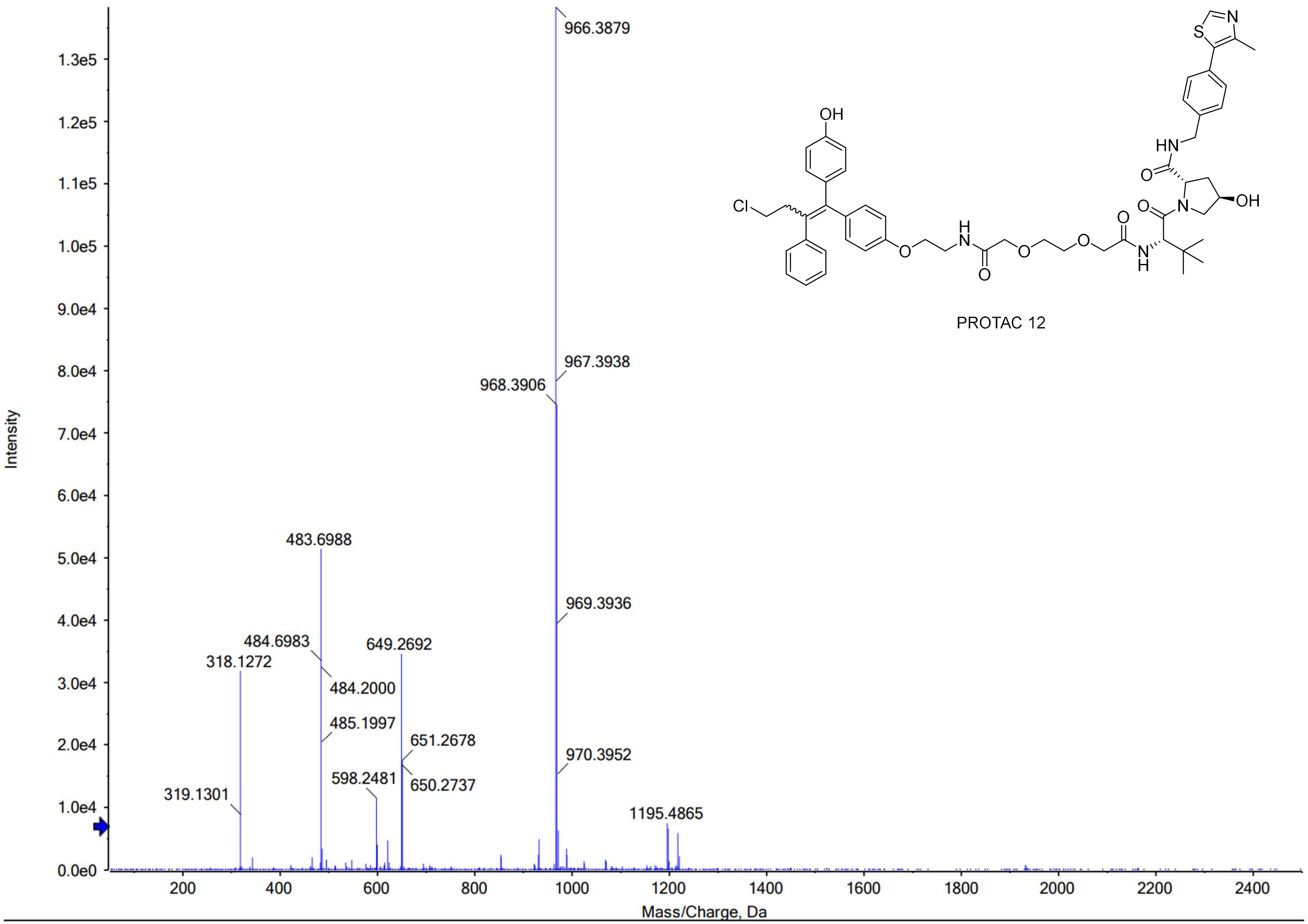

966.3879
967.3938
968.3906
969.3936
970.3952
483.6988
484.6983
484.2000
485.1997
318.1272
319.1301
649.2692
651.2678
650.2737
598.2481
1195.4865
Intensity
Mass/Charge, Da
OH
Cl
HN
O
N
S
PROTAC 12

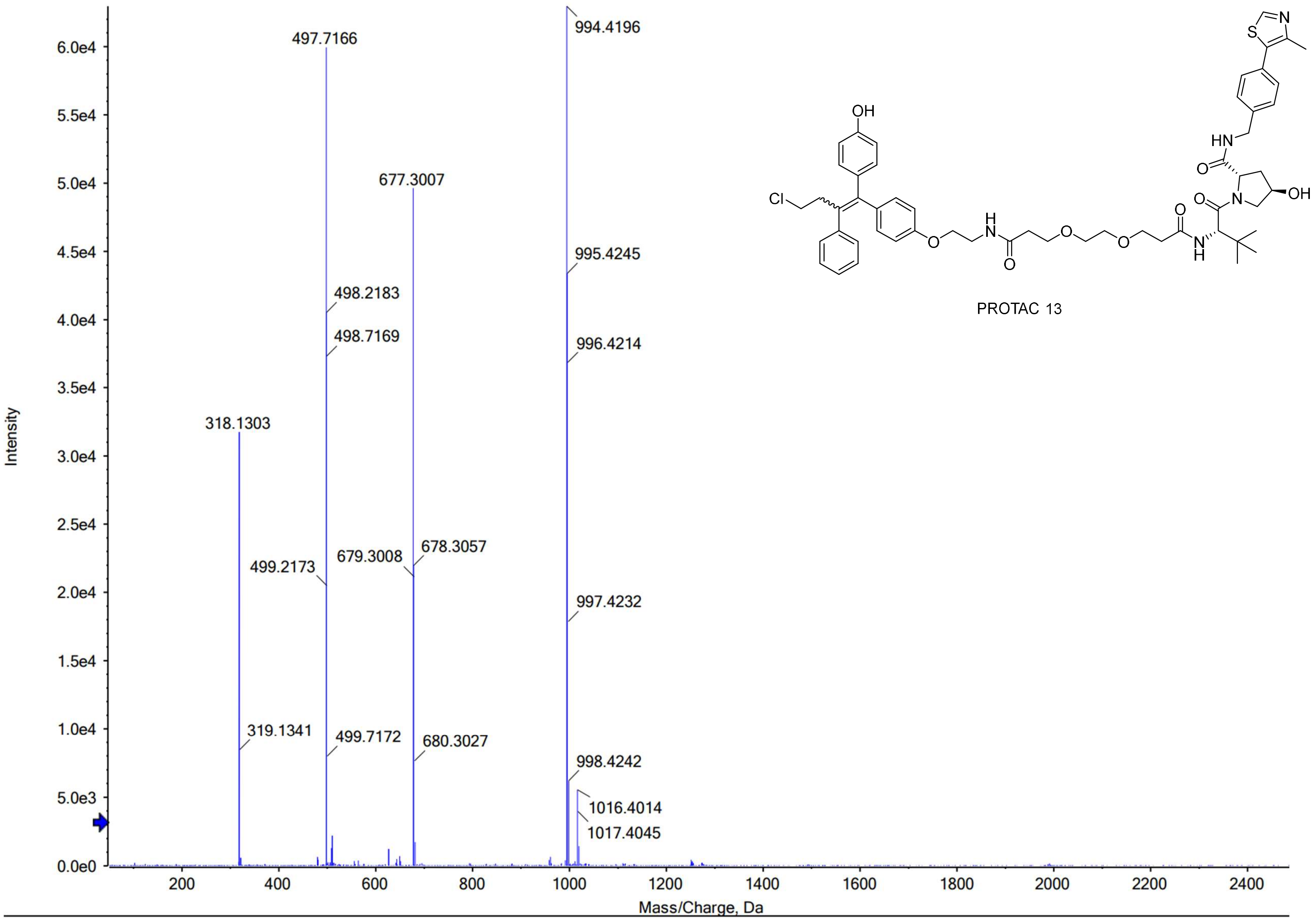

497.7166
994.4196
677.3007
995.4245
498.2183
498.7169
996.4214
318.1303
499.2173
679.3008
678.3057
997.4232
319.1341
499.7172
680.3027
998.4242
1016.4014
1017.4045
Intensity
Mass/Charge, Da

PROTAC 13

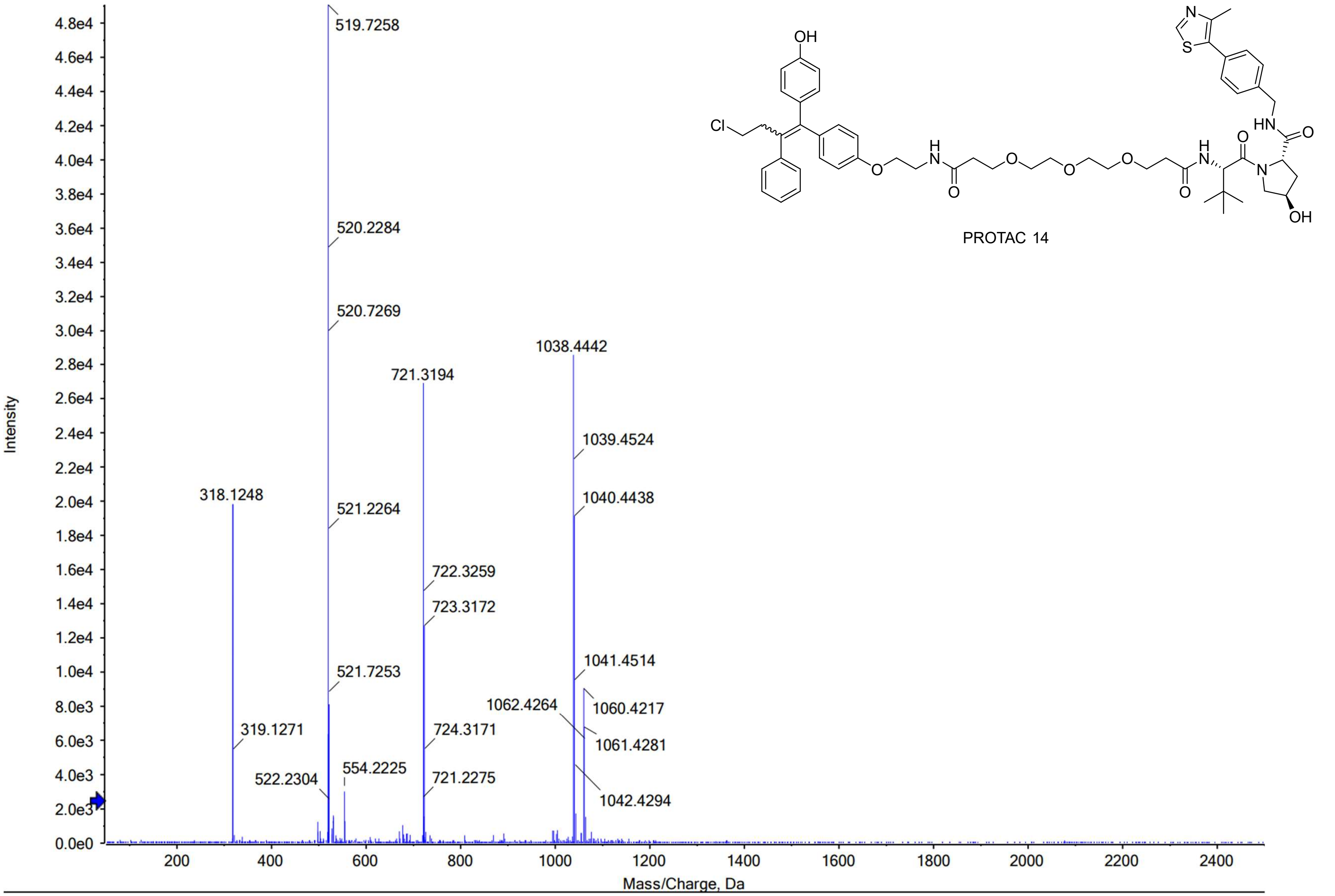
PROTAC 14
519.7258
520.2284
520.7269
1038.4442
721.3194
1039.4524
318.1248
1040.4438
521.2264
722.3259
723.3172
1041.4514
521.7253
1062.4264
1060.4217
319.1271
724.3171
1061.4281
554.2225
522.2304
721.2275
1042.4294
Intensity
Mass/Charge, Da

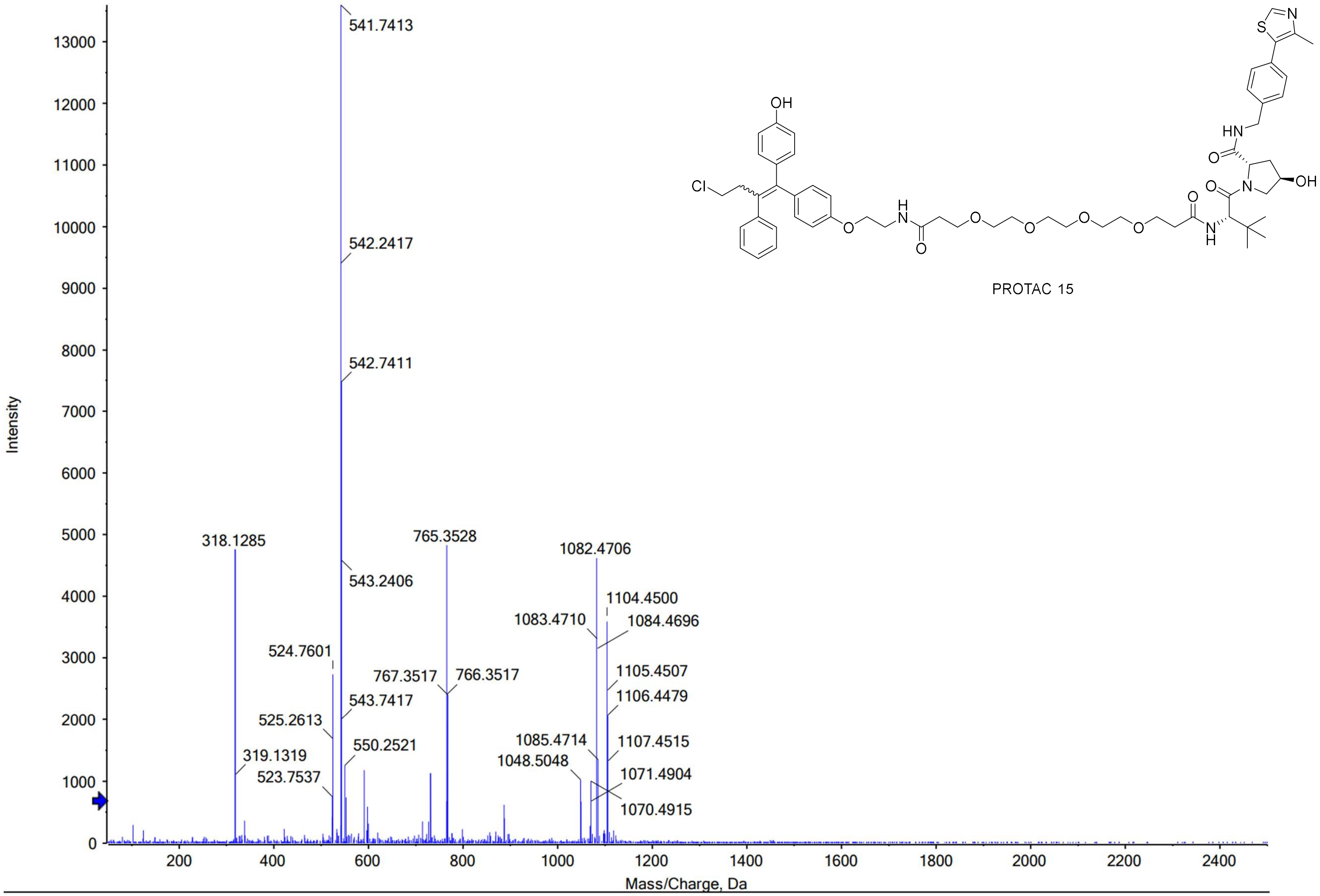
541.7413
542.2417
542.7411
543.2406
543.7417
318.1285
319.1319
765.3528
766.3517
767.3517
1082.4706
1083.4710
1084.4696
1085.4714
1104.4500
1105.4507
1106.4479
1107.4515
1048.5048
1071.4904
1070.4915
524.7601
525.2613
523.7537
550.2521
Intensity
Mass/Charge, Da
OH
Cl
HN
O
N
S
H
PROTAC 15

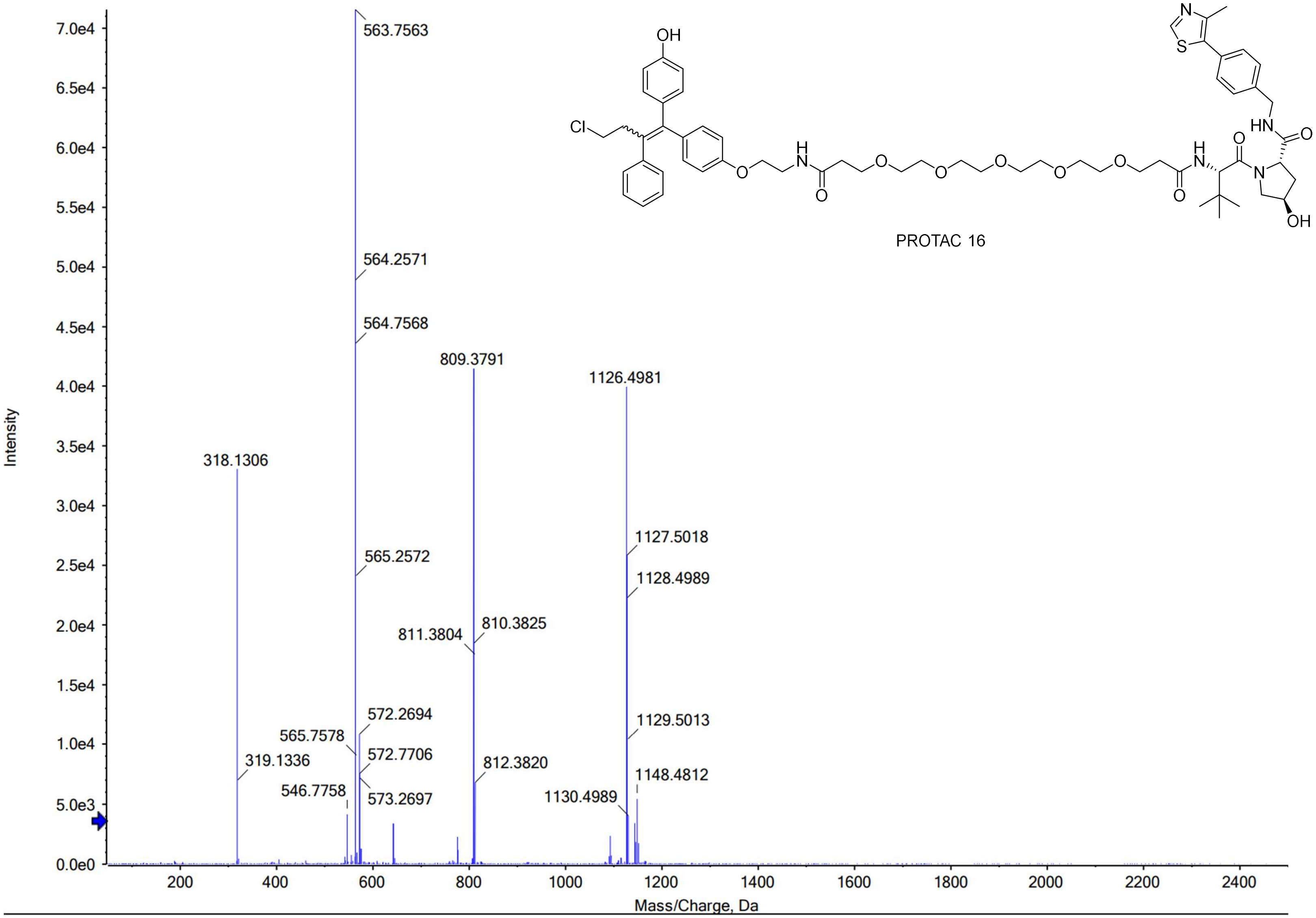
563.7563
564.2571
564.7568
809.3791
1126.4981
318.1306
1127.5018
565.2572
1128.4989
811.3804
810.3825
572.2694
565.7578
1129.5013
319.1336
572.7706
812.3820
546.7758
1148.4812
573.2697
1130.4989
Intensity
Mass/Charge, Da
PROTAC 16

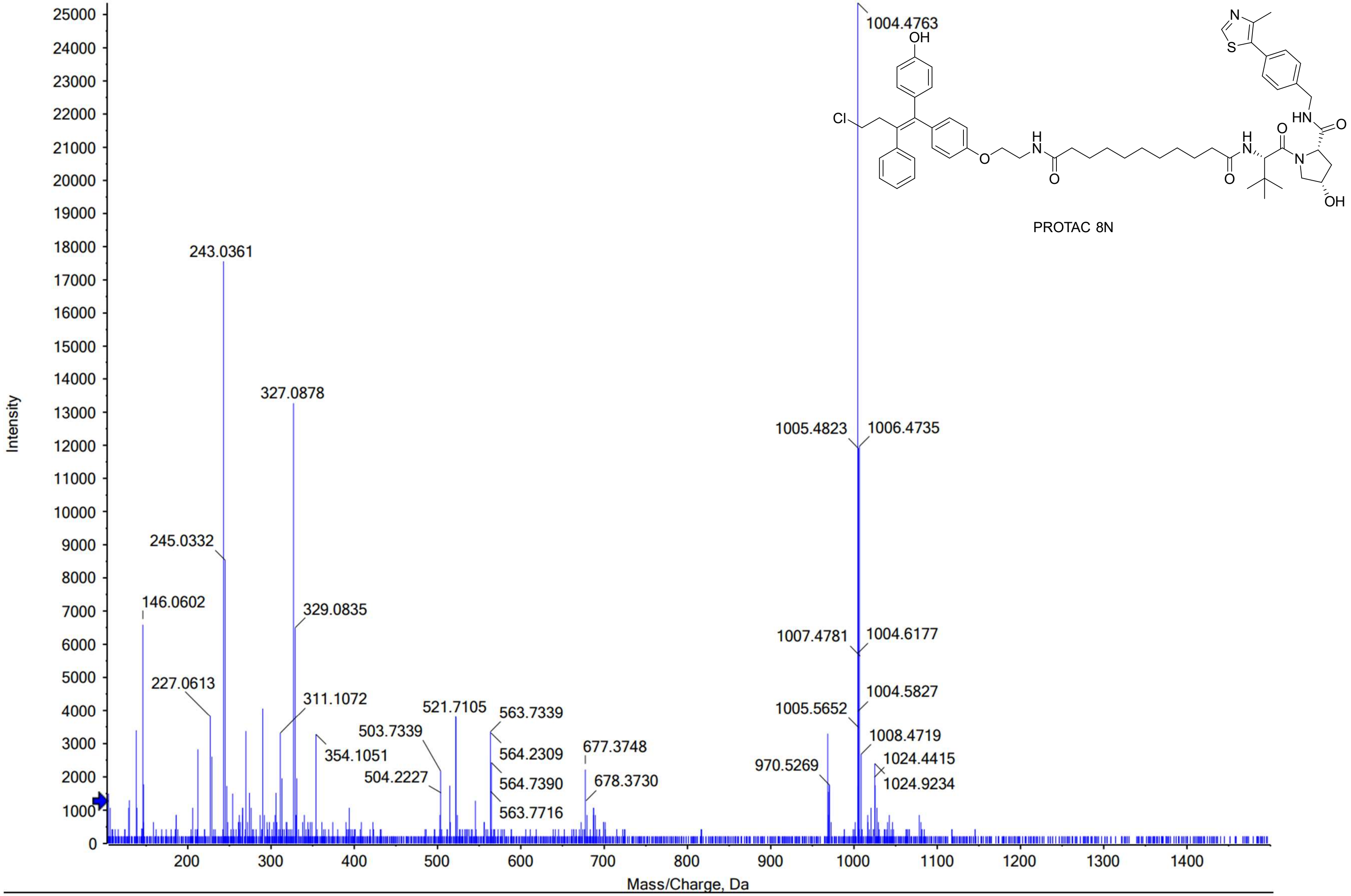

Intensity
Mass/Charge, Da
1004.4763
243.0361
327.0878
1005.4823
1006.4735
245.0332
146.0602
329.0835
1007.4781
1004.6177
227.0613
311.1072
1004.5827
1005.5652
521.7105
563.7339
503.7339
354.1051
564.2309
1008.4719
677.3748
970.5269
1024.4415
504.2227
564.7390
678.3730
1024.9234
563.7716
PROTAC 8N
