# Supplementary material for: DeepPROTACs is a deep learning-based targeted degradation predictor for PROTACs
Source: Nat Commun. 2022 Nov 21;13:7133. doi: 10.1038/s41467-022-34807-3 (PMC9681730; doi:10.1038/s41467-022-34807-3)
Supplement: Supplementary file 2 — Reporting Summary [file 41467_2022_34807_MOESM2_ESM.pdf]

nature portfolio

Corresponding author(s): Xiaobao Yang, Shenghua Gao, Fang Bai

Last updated by author(s): Nov 5, 2022

Reporting Summary

Nature Portfolio wishes to improve the reproducibility of the work that we publish. This form provides structure for consistency and transparency in reporting. For further information on Nature Portfolio policies, see our Editorial Policies and the Editorial Policy Checklist.

Statistics

For all statistical analyses, confirm that the following items are present in the figure legend, table legend, main text, or Methods section.

n/a	Confirmed	
☐	☒	The exact sample size (*n*) for each experimental group/condition, given as a discrete number and unit of measurement
☒	☐	A statement on whether measurements were taken from distinct samples or whether the same sample was measured repeatedly
☐	☒	The statistical test(s) used AND whether they are one- or two-sided<br>*Only common tests should be described solely by name; describe more complex techniques in the Methods section.*
☒	☐	A description of all covariates tested
☒	☐	A description of any assumptions or corrections, such as tests of normality and adjustment for multiple comparisons
☐	☒	A full description of the statistical parameters including central tendency (e.g. means) or other basic estimates (e.g. regression coefficient) AND variation (e.g. standard deviation) or associated estimates of uncertainty (e.g. confidence intervals)
☐	☒	For null hypothesis testing, the test statistic (e.g. *F*, *t*, *r*) with confidence intervals, effect sizes, degrees of freedom and *P* value noted<br>*Give P values as exact values whenever suitable.*
☒	☐	For Bayesian analysis, information on the choice of priors and Markov chain Monte Carlo settings
☒	☐	For hierarchical and complex designs, identification of the appropriate level for tests and full reporting of outcomes
☒	☐	Estimates of effect sizes (e.g. Cohen's *d*, Pearson's *r*), indicating how they were calculated

Our web collection on statistics for biologists contains articles on many of the points above.

Software and code

Policy information about availability of computer code

Data collection	No software was used for data collection.
Data analysis	A couple of softwares such as Yasara, Gromacs 2019 and Pymol 2.0 were used for data processing. By using the input data and corresponding labels, we trained a computational model (software) based on deep learning algorithms using Pytorch and Pytorch Geometric frameworks. The software has been available at https://bailab.siais.shanghaitech.edu.cn/services/deepprotacs/ for public use. The code is also available at github (https://github.com/fenglei104/DeepPROTACs).

For manuscripts utilizing custom algorithms or software that are central to the research but not yet described in published literature, software must be made available to editors and reviewers. We strongly encourage code deposition in a community repository (e.g. GitHub). See the Nature Portfolio guidelines for submitting code & software for further information.

Data

Policy information about availability of data

All manuscripts must include a data availability statement. This statement should provide the following information, where applicable:

- Accession codes, unique identifiers, or web links for publicly available datasets
- A description of any restrictions on data availability
- For clinical datasets or third party data, please ensure that the statement adheres to our policy

The PROTAC-DB database used in this study is available at http://cadd.zju.edu.cn/protacdb/. The ZINC database is available at https://zinc.docking.org/.

Human research participants

Policy information about studies involving human research participants and Sex and Gender in Research.

Reporting on sex and gender	N/A
Population characteristics	There are no human participants in this research.
Recruitment	There are no human participants in this research.
Ethics oversight	N/A

Note that full information on the approval of the study protocol must also be provided in the manuscript.

Field-specific reporting

Please select the one below that is the best fit for your research. If you are not sure, read the appropriate sections before making your selection.

[x] Life sciences [ ] Behavioural & social sciences [ ] Ecological, evolutionary & environmental sciences

For a reference copy of the document with all sections, see nature.com/documents/nr-reporting-summary-flat.pdf

Life sciences study design

All studies must disclose on these points even when the disclosure is negative.

Sample size	In this study, we collected almost all reported PROTACS from the literatures, the sample size is around 4000~, but only ~2800 of them have corresponding experimental data that can be used for developing our method. For experiments other than those including datasets, sample sizes were determined based on the requirements for testing our deep learning model or verifying our conclusion.
Data exclusions	We excluded the PROTACs that do not have experimentally determined degradation capacity. The exclusion criteria were widely accepted for supervised learning.
Replication	Results and corresponding analysis were repeated for three times. Under our test conditions, all repetitive attempts were successful, suggesting that the method has good reproducibility.
Randomization	For tuning the hyperparameters of model, we randomly split the whole dataset into training, validation and test set at a ratio of 8:1:1. For training the model, we randomly split the whole dataset into training and test set at a ratio of 8:2. And the data was shuffled in the process of training. For experiments other than those including datasets, samples were designed by ourself and allocation was not relevant to those experiments because we didn't know the allocation before experiments.
Blinding	In order to test the generalization ability of our model, we performed a single blind test to predict the degradation capability of 16 ER PROTACs which were not reported elsewhere and synthesized by our experimental collaborators. As a result, 68.75% accuracy was achieved.

Reporting for specific materials, systems and methods

We require information from authors about some types of materials, experimental systems and methods used in many studies. Here, indicate whether each material, system or method listed is relevant to your study. If you are not sure if a list item applies to your research, read the appropriate section before selecting a response.

Materials & experimental systems

n/a	Involved in the study	
[ ]	[x]	Antibodies
[ ]	[x]	Eukaryotic cell lines
[x]	[ ]	Palaeontology and archaeology
[x]	[ ]	Animals and other organisms
[x]	[ ]	Clinical data
[x]	[ ]	Dual use research of concern

Methods

n/a	Involved in the study	
[x]	[ ]	ChIP-seq
[x]	[ ]	Flow cytometry
[x]	[ ]	MRI-based neuroimaging

Antibodies

Antibodies used	ERalpha antibody (#8644S, Cell Signaling Technology) and GAPDH antibody (#8884S, Cell Signaling Technology)

Validation	*Describe the validation of each primary antibody for the species and application, noting any validation statements on the manufacturer's website, relevant citations, antibody profiles in online databases, or data provided in the manuscript.*

Eukaryotic cell lines

Policy information about cell lines and Sex and Gender in Research

Cell line source(s)	MCF-7, American Type Culture Collection; T-47D, American Type Culture Collection
Authentication	We did not perform further authentication for these commercial cell lines.
Mycoplasma contamination	We did not perform further Mycoplasma contamination test for these commercial cell lines.
Commonly misidentified lines (See ICLAC register)	No commonly misidentified cell lines were used in this study.
